# Koinetic: a graph-based distance as a new ecological metric for microbial community differential analysis

**DOI:** 10.1093/bib/bbag632

**Published:** 2026-11-15

**Authors:** Zihan Zhu, Yiqian Zhang, Ruitao Liu, Yi Duan, Wenhao Li, Zijing Huang, Xinhua Chen, Liangliang Zhang

**Affiliations:** Department of Population and Quantitative Health Sciences, School of Medicine, Case Western Reserve University, 10900 Euclid Ave., Cleveland, OH 44106, United States; Division of Biostatistics, College of Public Health, Ohio State University, 1841 Neil Ave., Columbus, OH 43210, United States; Department of Statistics, College of Arts and Sciences, The Ohio State University, 1958 Neil Ave., Columbus, OH 43210, United States; Department of Population and Quantitative Health Sciences, School of Medicine, Case Western Reserve University, 10900 Euclid Ave., Cleveland, OH 44106, United States; State Key Laboratory of Immune Response and Immunotherapy, Department of Infectious Diseases, The First Affiliated Hospital of USTC, Center for Advanced Interdisciplinary Science and Biomedicine of IHM, Division of Life Sciences and Medicine, University of Science and Technology of China, 96 Jinzhai Road, Baohe District, Hefei, Anhui 230026, China; Key Laboratory of Anhui Province for Emerging and Reemerging Infectious Diseases, 443 Huangshan Road, Shushan District, Hefei, Anhui 230027, China; Department of Biostatistics, The University of Texas MD Anderson Cancer Center, 7007 Bertner Ave., Houston, TX 77030, United States; State Key Laboratory of Immune Response and Immunotherapy, Department of Infectious Diseases, The First Affiliated Hospital of USTC, Center for Advanced Interdisciplinary Science and Biomedicine of IHM, Division of Life Sciences and Medicine, University of Science and Technology of China, 96 Jinzhai Road, Baohe District, Hefei, Anhui 230026, China; Department of Hepatobiliary-Pancreatic Surgery, The First Affiliated Hospital, Zhejiang University School of Medicine, 79 Qingchun Road, Shangcheng District, Hangzhou 310003, Zhejiang, China; Department of Population and Quantitative Health Sciences, School of Medicine, Case Western Reserve University, 10900 Euclid Ave., Cleveland, OH 44106, United States; Case Comprehensive Cancer Center, 2103 Cornell Road, University Circle, Cleveland, OH 44106, United States

**Keywords:** beta diversity, dissimilarity metric, phylogeny-aware analysis, microbiome analysis, graph Laplacian

## Abstract

Phylogeny-aware beta diversity measures are essential in microbiome research, yet widely used metrics such as UniFrac and its variants rely on independent branch-length contributions and do not satisfy the Euclidean property, leading to distortions in ordination and kernel-based tests. These limitations underscore the need for a distance that is both phylogenetically coherent and geometrically valid. We introduce the Koinos Kinetic Distance (Koinetic), a phylogeny-aware dissimilarity that views microbial communities as interconnected assemblies rather than isolated taxa. ”Koinos” emphasizes collective evaluation, while ”kinetic” reflects how differences propagate dynamically across ecological networks and phylogenetic lineages. Koinetic leverages a graph Laplacian–based kernel to weight community differences by shared phylogeny, capturing ecological connectivity and clade relatedness. This design ensures Euclidean validity and enables seamless integration with kernel-based methods as well as geometry-preserving ordination. Controlled simulations and analyses of eight microbiome datasets spanning diverse body sites, disease contexts, and microbial profiling modalities demonstrate Koinetic’s broad applicability. Compared with classical and phylogenetic metrics, including weighted, unweighted, and generalized UniFrac, unweighted Koinetic can improve power for phylogenetically localized prevalence shifts in the simulated settings, while weighted Koinetic performs comparably with weighted and generalized UniFrac under abundance-driven signals. Koinetic also shows strong performance in PERMANOVA and MiRKAT analyses assessing associations between microbial community composition and host phenotypes. Koinetic thus provides a general foundation for phylogeny-aware analysis, with a consistent geometric basis for visualization and statistical inference.

## Introduction

Quantifying and comparing differences across microbial communities is fundamental for ecological and biomedical studies. Such comparisons inform our understanding of environmental adaptation, host–microbe interactions, and evolutionary dynamics [1, 2]. Dissimilarity metrics lie at the heart of these analyses, providing the numerical basis for ordination, clustering, and hypothesis testing [3].

When a phylogenetic tree is available, dissimilarity measures can incorporate evolutionary relationships among taxa rather than treating them as independent categories. Such tree-based approaches provide a principled extension of prevalence- or abundance-based comparisons by accounting for the distribution of lineages across phylogeny [4, 5]. Before introducing these measures, we first outline the structure and biological significance of the phylogenetic trees.

A phylogenetic tree is a directed, acyclic graph that encodes the inferred evolutionary history of a set of taxa, typically reconstructed from molecular sequence alignments or, less commonly, morphological characters [6]. Inference generally combines multiple-sequence alignment, a model of sequence evolution, and an optimality criterion based on distance, parsimony, likelihood, or Bayesian inference [7–9]. Statistical support for the resulting topology is often assessed through resampling methods such as bootstrapping [10]. The resulting tree carries two complementary layers of information. Topology, defined by the pattern of nodes and bifurcations, reveals clade membership and the order of divergence events [5, 11]. In contrast, branch lengths provide quantitative measures of evolutionary distance, allowing comparisons of the amount or rate of change between taxa [12, 13]. Together, these features form an informational basis for phylogeny-aware biodiversity metrics and community dissimilarities.

Early efforts to incorporate phylogeny into diversity analysis focused on *α *-diversity, most notably Faith’s PD and its generalization within the Hill-number framework [14, 15]. For *β *-diversity, UniFrac and its weighted and generalized variants extended these ideas to between-community comparisons by integrating branch length with taxon presence or abundance [4, 16, 17]. While these metrics establish the value of phylogeny-aware measures, their formulation can be further improved in two aspects. First, they aggregate phylogenetic differences in an edge-wise additive manner, treating branches independently and therefore not explicitly encoding the coupling structure induced by shared internal nodes, clades, or bundles of closely related evolutionary paths. Second, many commonly used phylogeny-aware dissimilarities are not Euclidean [18].

The Euclidean property is important because dissimilarity measures are rarely used for a single analysis. The same dissimilarity matrix is commonly used for ordination, clustering, association testing, and kernel-based analyses. When a dissimilarity is non-Euclidean, the original pairwise relationships cannot be represented exactly in a Euclidean coordinate space. As a result, samples that are far apart according to the original dissimilarity may appear artificially close after ordination, whereas apparently separated clusters may partly reflect geometric distortion rather than biological structure. This phenomenon is referred to as the illusion of similarity [18]. Such distortions can affect neighborhood relationships, sample ranking, cluster interpretation, and the apparent degree of group separation. Their severity is reflected by the magnitude of the negative eigenvalues produced by principal coordinate analysis [18]. Although methods such as PERMANOVA can still be computed from a non-Euclidean dissimilarity matrix, the resulting analysis is based on an indefinite geometry, and *post hoc* corrections may be required. Thus, the concern does not lie with PERMANOVA itself, but with the geometry induced by the dissimilarity supplied to it. A Euclidean dissimilarity is therefore desirable because it preserves a coherent geometric interpretation of group separation without requiring *post hoc* correction. A formal characterization of the Euclidean property, together with a detailed explanation of how negative eigenvalues affect the geometric interpretation of PERMANOVA and related downstream analyses, is provided in Supplementary Section 1.1.

We advance phylogeny-aware *β *-diversity by introducing the Koinos Kinetic Distance (Koinetic) and its presence–absence analog, unweighted koinetic. “Koinos” emphasizes collective evaluation, while “kinetic” reflects how differences propagate dynamically across ecological networks and phylogenetic lineages. By embedding the weighted phylogenetic tree in a positive-definite Laplacian kernel, koinetic preserves both tree topology and branch length information while guaranteeing Euclidean validity. This formulation enables seamless integration with kernel-based statistical methods, PERMANOVA, and geometry-preserving ordination—capabilities that UniFrac and its variants do not provide.

## Materials and methods

In this section, we first revisit UniFrac, the most widely used phylogeny-aware dissimilarity, to clarify its formulation and scope, and then develop koinetic as an alternative. Throughout, we use the toy phylogenetic tree in Fig. 1 as a running example to streamline notation and interpretation. Figure 1 depicts a toy phylogenetic tree comprising five observed operational taxonomic units (OTUs), three internal ancestral nodes, and a root. To illustrate the key components involved in constructing koinetic and their biological interpretations, we consider two microbial communities characterized by the following tip-level abundance table:


\begin{align*} \begin{array}{lccccc} \hline & {\mathrm{OTU1}} & {\mathrm{OTU2}} & {\mathrm{OTU3}} & {\mathrm{OTU4}} & {\mathrm{OTU5}} \\ \hline{\mathrm{Sample\, R}} & 30 & 20 & 10 & 0 & 0 \\{\mathrm{Sample\, S}} & 8 & 8 & 31 & 31 & 23 \\ \hline \end{array} \end{align*}

**Figure 1:**
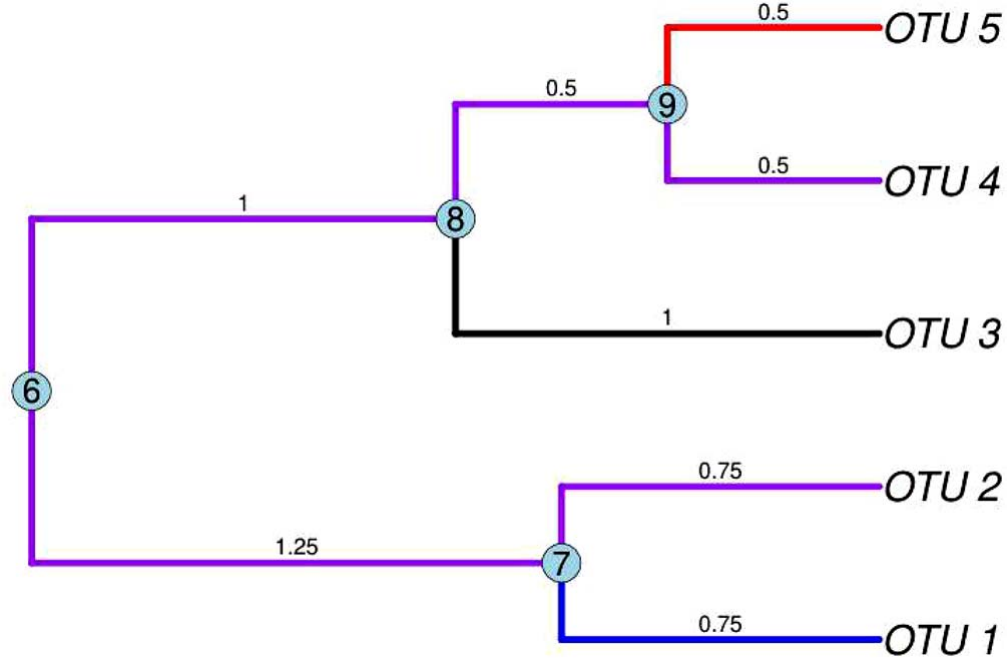
Example phylogenetic tree used throughout the Methods section. The tips correspond to OTUs 1–5; nodes 7–9 are internal bifurcations, and node 6 is the root. The edge labels denote branch lengths (substitutions per site).

This simplified example illustrates the mathematical definitions and highlights the ecological rationale underpinning our distance formulation. The formal definition of Koinetic and the proof of its Euclidean property are provided in Supplementary Section 1.2.

### UniFrac distances

We begin by reviewing UniFrac, the most widely used phylogeny-aware *β *-diversity measure in microbiome studies, which provides a natural baseline for incorporating evolutionary information into between-community comparisons.

Unweighted UniFrac quantifies the dissimilarity between two samples by computing the total branch length in the phylogenetic tree that is uniquely represented in either community, normalized by the total branch length observed in their union [4],


\begin{align*}& \mathrm{UniFrac}(R,S) = \frac{\sum_{j} b_{j}|p^{R}_{j} - p^{S}_{j}|}{\sum_{j} b_{j}I(p^{R}_{j} + p^{S}_{j}>0)}, \end{align*}

where $b_{j}$ denotes the branch length from node *j* to its parent node. Throughout, *R* and *S* denote two samples, $p_{j}^{R}$ and $p_{j}^{S} \in \{0,1\}$ indicate the presence–absence status of taxon *j*, and $r_{j}^{R}$ and $r_{j}^{S}$ denote the corresponding relative abundances. By construction, unweighted UniFrac treats taxa in a binary fashion (presence or absence), emphasizing lineage turnover while ignoring differences in abundance. In the toy example shown in Fig. 1, the branches unique to either sample include $8 \to 9, 9 \to 4,$ and *9 → 5*, with a total length of 1.5. The union includes all branches in the tree, totaling 6.25. Thus, the unweighted UniFrac distance between samples R and S is *1.5 / 6.25 = 0.24*. Unweighted UniFrac is a metric but not Euclidean [18, 19], and distance-based downstream analyses such as principal coordinates analysis therefore rely on standard eigenvalue corrections, including the Cailliez additive-constant transformation [20] or the Lingoes shift [21].

Weighted UniFrac extends the unweighted version by incorporating relative abundance information and measuring the total branch length weighted by the absolute difference in lineage abundances between two communities [16],


\begin{align*}& \mathrm{WUniFrac}(R,S) = \frac{\sum_{j} b_{j}|r^{R}_{j} - r^{S}_{j}|}{\sum_{j} b_{j}|r^{R}_{j} + r^{S}_{j}|}. \end{align*}

This formulation highlights shifts among dominant taxa while still accounting for phylogenetic relationships. In the toy example, the numerator is approximately 2.70 and the denominator is 4.00, yielding a normalized weighted UniFrac distance of *∼ 0.675 *. As with the unweighted version, weighted UniFrac is typically non-Euclidean [18].

Under this formulation, UniFrac distances quantify community differences by aggregating branch-specific contributions along the phylogenetic tree, treating each branch as an independent carrier of dissimilarity. This branch-wise construction has proven effective in capturing lineage turnover and abundance shifts, but it does not explicitly encode how differences propagate through shared internal nodes, clades, or extended evolutionary paths. These observations motivate a complementary perspective in which the phylogenetic tree is treated as a coupled graph structure, allowing evolutionary proximity to mediate global evolutionary covariances among taxa.

### Overview of koinetic

Koinetic is motivated by viewing phylogenetic *β *-diversity as a problem of how compositional differences propagate over an evolutionary structure, rather than as a purely branch-wise comparison. To address this, koinetic represents the phylogenetic tree as a weighted graph and models evolutionary proximity through a graph Laplacian operator. Graph Laplacians are a natural tool for encoding neighborhood structure and connectivity, widely used to describe diffusion, smoothing, and signal propagation on networks [22]. In the phylogenetic context, this formulation allows both local branching patterns and longer shared paths to jointly influence similarity, enabling differences at one taxon to reverberate across closely related lineages through shared internal nodes and clades. This perspective treats the tree as a coupled system rather than a collection of independent branches.

To translate this graph-based representation into a valid dissimilarity measure, koinetic adopts Rao’s quadratic-entropy framework [23], in which between-sample differences are weighted by an evolutionary covariance matrix encoding evolutionary similarity. By deriving this evolutionary covariance matrix from the inverse graph Laplacian, koinetic naturally integrates phylogenetic tree topology and branch lengths while ensuring that the resulting distance is Euclidean. The following sections detail each component of this construction, beginning with the graph representation of the phylogenetic tree.

### Evolutionary proximity matrix

A phylogeny can be represented as a graph whose nodes correspond to the tips and internal ancestors and whose edges correspond to the branches connecting them. Based on this representation, we define an evolutionary proximity matrix $\mathbf{A}$, analogous to a weighted adjacency matrix, where the entry $A_{i,j}$ in row *i*, column *j* equals the length of the branch joining nodes *i* and *j* (and is zero if no branch exists). For the toy tree shown in Fig. 1, the evolutionary proximity matrix can be quantified as


\begin{align*}& \mathbf{A}= \begin{array}{@{}c@{}} \\ OTU 1 \\ OTU 2 \\ OTU 3 \\ OTU 4 \\ OTU 5 \\ Node 6\\ Node 7\\ Node 8\\ Node 9 \end{array} \begin{array}{@{}c@{}} OTU 1 \ OTU 2 \ OTU 3 \ OTU 4 \ OTU 5 \ Node 6 \ Node 7 \ Node 8 \ Node 9 \\ \left(\begin{array}{@{}ccccccccc@{}} 0 & 0 & 0 & 0 & 0 & 0 & 0.75 & 0 & 0 \nonumber\\ 0 & 0 & 0 & 0 & 0 & 0 & 0.75 & 0 & 0 \nonumber\\ 0 & 0 & 0 & 0 & 0 & 0 & 0 & 1 & 0 \nonumber\\ 0 & 0 & 0 & 0 & 0 & 0 & 0 & 0 & 0.5 \nonumber\\ 0 & 0 & 0 & 0 & 0 & 0 & 0 & 0 & 0.5 \nonumber\\ 0 & 0 & 0 & 0 & 0 & 0 & 1.25 & 1 & 0 \nonumber\\ 0.75 & 0.75 & 0 & 0 & 0 & 1.25 & 0 & 0 & 0 \nonumber\\ 0 & 0 & 1 & 0 & 0 & 1 & 0 & 0 & 0.5 \nonumber\\ 0 & 0 & 0 & 0.5 & 0.5 & 0 & 0 & 0.5 & 0 \nonumber\\ \end{array}\right)\end{array} \end{align*}

The matrix encodes the phylogenetic tree topology and branch lengths without loss of information, so the tree can be reconstructed from it. For example, $A_{1,7} = 0.75$ indicates that OTU 1 and internal node 7 are directly connected by a branch of length 0.75, while $A_{1,2} = 0$ indicates that OTUs 1 and 2 are not directly connected. This matrix serves as the foundation for constructing graph Laplacians that encode evolutionary proximity, enabling phylogeny-aware dissimilarity metrics. Throughout the main text, we assume that all branch lengths are strictly positive. Cases involving zero-length branches, which may arise from unresolved polytomies or lack of evolutionary divergence [24, 25], are discussed in detail in Supplementary Section 1.2 (Definition of GLaD).

### Degree matrix

The degree of a node in the phylogenetic tree, defined as the sum of the branch lengths of all edges incident to that node, quantifies the total evolutionary distance linking that node to its immediate neighbors. It captures the cumulative extent of divergence (for internal ancestral nodes) and phylogenetic isolation (for terminal OTUs). Let $\mathbf{D}$ denote the diagonal degree matrix, with entry $D_{j,j}$ representing the degree of node *j*. This value can be computed by summing row *j* of the evolutionary proximity matrix $\mathbf{A}$, which is the sum of the lengths of the branches leading from node *j* to other nodes. For example, node 7 is connected with OTU 1, OTU 2, and node 6, so $D_{7,7} = A_{7,1} + A_{7,2} + A_{7,6} = 2.75$. As a result, the diagonal degree matrix of the toy example is


\begin{align*} & \mathbf{D}=\mathrm{diag}(0.75,0.75,1,0.5,0.5,2.25,2.75,2.5,1.5). \end{align*}

### Graph Laplacian

To formally integrate both the topology and evolutionary information, we construct a graph-theoretic Laplacian matrix from the phylogenetic tree $\mathbf{L}=\mathbf{D}-\rho \mathbf{A}$ [22, 26]. The parameter $\rho \in [0, 1)$ modulates the evolutionary autocorrelation strength—interpreted as how closely related taxa are expected to resemble one another. For example, if *ρ = 0.8*, Fig. 1 yields the Laplacian matrix:


\begin{align*} & \mathbf{L} = \begin{bmatrix} 0.75 & 0 & 0 & 0 & 0 & 0 & -0.60 & 0 & 0 \\ 0 & 0.75 & 0 & 0 & 0 & 0 & -0.60 & 0 & 0 \\ 0 & 0 & 1.00 & 0 & 0 & 0 & 0 & -0.80 & 0 \\ 0 & 0 & 0 & 0.50 & 0 & 0 & 0 & 0 & -0.40 \\ 0 & 0 & 0 & 0 & 0.50 & 0 & 0 & 0 & -0.40 \\ 0 & 0 & 0 & 0 & 0 & 2.25 & -1.00 & -0.80 & 0 \\ -0.60 & -0.60& 0 & 0 & 0 & -1.00& 2.75 & 0 & 0 \\ 0 & 0 & -0.80& 0 & 0 & -0.80& 0 & 2.50 & -0.40 \\ 0 & 0 & 0 & -0.40& -0.40& 0 & 0 & -0.40 & 1.50 \end{bmatrix} \end{align*}

In graph theory, the Laplacian matrix serves as a fundamental operator that encodes the diffusion of a signal over the graph structure. For koinetic, this formulation reflects the extent to which evolutionary proximity translates into ecological similarity. If *ρ = 1*, we assume perfect trait conservation along the tree: nearby nodes (e.g. sibling OTUs) are expected to share ecological roles. By contrast, *ρ = 0* discards all phylogenetic information and treats each node as isolated. Intermediate values strike a balance between these extremes, accommodating partial phylogenetic signal while mitigating the undue influence of deep branches with sparse descendants.

When *ρ = 1*, the classical graph Laplacian is recovered, which is inherently singular. From a computational biology perspective, this singularity arises because the root of a phylogenetic tree does not contribute additional degrees of freedom. In modern workflows for processing 16S rRNA or metagenomic sequence data, trees are often inferred as unrooted using methods such as FastTree or RAxML, and then artificially rooted for interpretability and visualization [9]. Whether the root is specified *post hoc* (as QIIME1) [27] or integrated into phylogenetic placement algorithms (as in QIIME2 or SEPP-based workflows) [28, 29], it functions solely as a reference point, not as an independently inferred node. Consequently, the effective dimension of the tree structure is constrained by the upper bound *n-1*, rendering $\mathbf{L}$ singular whenever *ρ = 1*. We discuss the construction of evolutionary covariance and distance under *ρ = 1* in Supplementary Section 1.3 (GLaD with the Classical Graph Laplacian).

### Evolutionary covariance matrix

While the evolutionary proximity matrix $ \mathbf{A} $ encodes only direct evolutionary connections, i.e. whether two nodes are immediately joined by a branch, phylogenetic similarity is rarely so localized. Most microbial taxa diverged millions of years ago and now reside on distant branches; however, their ecological similarity often reflects shared ancestry along entire paths in the tree, not just immediate proximity. To capture this, we turn to the inverse graph Laplacian $ \mathbf{B} = \mathbf{L}^{-1} $, which acts as an evolutionary covariance matrix; the matrix construction and computational details are provided in Supplementary Section 1.4. Mathematically, it encodes how strongly fluctuations at one node ”propagate” to another, through all available paths weighted by branch length and topology. In contrast to $ \mathbf{A} $, which is sparse and local, $ \mathbf{B} $ is dense and integrated. Formally, when $\mathbf{L} = \mathbf{D} - \rho \mathbf{A}$ is invertible with $\rho \in [0,1)$, the inverse admits the Neumann series expansion


(1) \begin{align*}& \mathbf{B} = (\mathbf{D} - \rho \mathbf{A})^{-1} = \mathbf{D}^{-1/2} \left( \sum_{k=0}^{\infty} \rho^{k} \mathbf{\Omega}^{k} \right) \mathbf{D}^{-1/2}, \qquad \mathbf{\Omega} = \mathbf{D}^{-1/2} \mathbf{A} \mathbf{D}^{-1/2}.\end{align*}

Equivalently, at the entry level,


(2) \begin{align*}& B_{ij} = \sum_{k=0}^{\infty} \rho^{k} \sum_{\mathrm{walk} i\to j} \frac{\prod_{(u,v)\in \mathrm{walk}} \ell_{uv}} {\prod_{u\in \text{walk nodes}} d_{u}},\end{align*}

where $\ell _{uv}$ denotes the branch length on edge *(u,v)* and $d_{u} = \sum _{v} \ell _{uv}$ is the degree of node *u*. Because a phylogenetic tree contains a unique simple path between any two nodes, the dominant contribution arises from that path. Denoting this path by $\mathcal{P}(i,j)$ with *k(i,j)* steps, we obtain the approximation


(3) \begin{align*}& B_{ij} \;\approx\; \rho^{\,k(i,j)} \, \frac{\prod_{(u,v)\in \mathcal{P}(i,j)} \ell_{uv}} {\prod_{u\in \mathcal{P}(i,j)} d_{u}}.\end{align*}

It shows that the evolutionary covariance between nodes *i* and *j* arises from the product of branch lengths along their unique connecting path, normalized by the degrees of all intermediate nodes. In other words, $B_{ij}$ measures the cumulative evolutionary coupling along the shared ancestral backbone, with each branch weighted by its length and each internal node contributing a dilution factor proportional to its total incident branch length.

Two primary structural factors determine the magnitude of this evolutionary covariance.

(a) Hierarchical proximity. The shorter the path between *i* and *j* (i.e. the fewer intermediate steps *k(i,j)*), the larger $B_{ij}$ becomes, due to reduced attenuation from the factor $\rho ^{k(i,j)}$ and fewer degree-normalization terms.
(b) Relative branch dominance. Along the connecting path, evolutionary covariance strengthens when each branch length $\ell _{uv}$ constitutes a large proportion of the corresponding node degree $d_{u}$. That is, evolutionary links that account for a substantial share of a node’s total divergence contribute more strongly to cross-lineage coupling.

These observations suggest the following phylogenetic assumption under our formulation: phylogenetic similarity is strong between taxa that (i) diverge recently through a small number of ancestral splits and (ii) are connected by evolutionary branches that dominate the divergence structure of their ancestral nodes, rather than being minor offshoots within highly branched clades.

For the toy tree in Fig. 1, Fig. 2 presents the evolutionary covariance matrix $\mathbf{B}$ numerically and as a heatmap.

**Figure 2:**
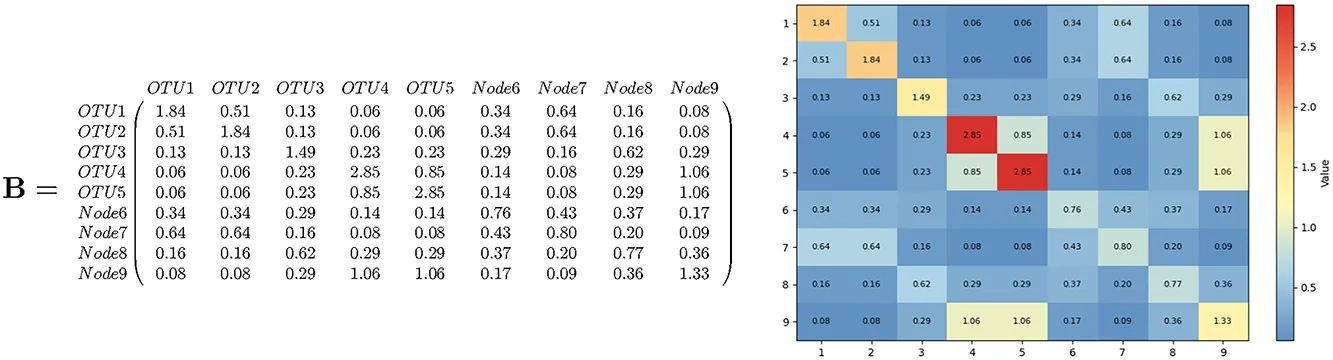
Matrix $\mathbf{B}$ (left) and its heatmap visualization (right).

It integrates the shared evolutionary history of the OTUs and local branching geometry. We interpret the evolutionary covariance matrix $\mathbf{B} = \mathbf{L}^{-1}$ by separately examining its diagonal and off-diagonal entries:

(1) Diagonal elements ($B_{ii}$) reflect the uncertainty associated with node *i*’s placement in the tree. This uncertainty arises from both the evolutionary depth and local branching complexity. Greater depth implies longer evolutionary divergence and hence larger uncertainty in ecological traits, while richer local branching (e.g. many closely related clades) increases evolutionary uncertainty. For instance, OTU 4 and OTU 5 have the highest diagonal values, indicating both deep phylogenetic placement and rich sibling diversification in their evolutionary history. In contrast, the root node has the smallest diagonal value, because it represents the most conserved ancestral state.
(2) Off-diagonal elements ($B_{ij}$) capture the evolutionary covariance between OTUs *i* and *j*. Its magnitude is determined by hierarchical proximity and relative branch dominance as discussed above. For example, although the direct branch between Node 8 and Node 9 is shorter than that between Node 8 and OTU 3 in the evolutionary proximity matrix, the corresponding evolutionary covariance in $\mathbf{B}$ is larger for the pair (8,3) than for (8,9). This occurs because Node 9 is itself a branching point with multiple outgoing edges, so the edge between Nodes 8 and 9 represents only a fraction of the total divergence at Node 9. In contrast, OTU 3 connects to Node 8 through a branch that constitutes the dominant share of its evolutionary divergence. After degree normalization, this dominant evolutionary link results in a stronger global association in $\mathbf{B}$. Consequently, the off-diagonal entries of $\mathbf{B}$ reflect not only raw branch lengths but also their relative importance within the local branching structure of the tree.

### Weighted and unweighted koinetic

Quantifying the dissimilarity between samples based on an evolutionary covariance matrix among OTUs has a foundation in numerical ecology. Rao [23] introduced the framework of quadratic entropy, from which a general class of dissimilarities incorporating inter-species distances was developed [30]:


\begin{align*}& D_{\mathrm{Rao}}(R,S) = \sqrt{(\mathbf{r}^{R} - \mathbf{r}^{S})^{\top} \mathbf{\Delta} (\mathbf{r}^{R} - \mathbf{r}^{S})}. \end{align*}

Here, $\mathbf{\Delta }$ denotes the Gram matrix derived from inter-species distances, which can be interpreted as an evolutionary covariance matrix among species [31, 32], and $\mathbf{r}^{R}, \mathbf{r}^{S}$ are the relative abundance vectors of samples *R* and *S*. To simultaneously account for the phylogenetic topology and branch lengths, a natural extension is to replace $\mathbf{\Delta }$ with the inverse graph Laplacian $\mathbf{B}$. As shown in [31, 33, 34], the resulting dissimilarity remains Euclidean provided that $\mathbf{B}$ is positive semidefinite. Building on this foundation, we formally introduce the following distance measure.

We first propagate the observed OTU abundances upward along the phylogenetic tree, summing values at each internal node and the root based on all descendant tips. This results in a full abundance profile over the entire tree structure. For example, the aggregated absolute abundance table for our toy example is:


\begin{align*} \begin{array}{l@{\ \ \ }c@{\ \ \ }c@{\ \ \ }c@{\ \ \ }c@{\ \ \ }c@{\ \ \ }c@{\ \ \ }c@{\ \ \ }c@{\ \ \ }c} \hline & {\mathrm{OTU1}} & {\mathrm{OTU2}} & {\mathrm{OTU3}} & {\mathrm{OTU4}} & {\mathrm{OTU5}} & {\mathrm{Node\, 7}} & {\mathrm{Node\, 9}} & {\mathrm{Node\, 8}} & {\mathrm{Root\, 6}} \\ \hline{\mathrm{Sample\, R}} & 30 & 20 & 10 & 0 & 0 & 50 & 0 & 10 & 60 \\{\mathrm{Sample\, S}} & 8 & 8 & 31 & 31 & 23 & 16 & 54 & 85 & 101 \\ \hline \end{array} \end{align*}

The corresponding aggregated relative abundance table is:


\begin{align*} \begin{array}{l@{\ \ \ }c@{\ \ \ }c@{\ \ \ }c@{\ \ \ }c@{\ \ \ }c@{\ \ \ }c@{\ \ \ }c@{\ \ \ }c@{\ \ \ }c} \hline & {\mathrm{OTU1}} & {\mathrm{OTU2}} & {\mathrm{OTU3}} & {\mathrm{OTU4}} & {\mathrm{OTU5}} & {\mathrm{Node\, 7}} & {\mathrm{Node\, 9}} & {\mathrm{Node\, 8}} & {\mathrm{Root\, 6}} \\ \hline{\mathrm{Sample\, R}} & 1/2 & 1/3 & 1/6 & 0 & 0 & 5/6 & 0 & 1/6 & 1 \\{\mathrm{Sample\, S}} & 8/101 & 8/101 & 31/101 & 31/101 & 23/101 & 16/101 & 54/101 & 85/101 & 1 \\ \hline \end{array} \end{align*}

In the weighted version of koinetic, we normalize each sample vector by its total sequencing depth to obtain relative abundances. The dissimilarity between two samples *R* and *S* is then computed using the relative abundance difference $\mathbf{r}^{R} - \mathbf{r}^{S}$ and the evolutionary covariance matrix $\mathbf{B}$, following Rao’s quadratic form:


(4) \begin{align*}& d_{S}^{W}(R,S) = \sqrt{ (\mathbf{r}^{R} - \mathbf{r}^{S})^{\top} \mathbf{B} (\mathbf{r}^{R} - \mathbf{r}^{S})}.\end{align*}

For the unweighted version, we use binarized presence–absence vectors $\mathbf{p}^{R}$ and $\mathbf{p}^{S}$, which equal 1 if any descendant taxon is observed and 0 otherwise. The unweighted koinetic is computed as follows:


(5) \begin{align*}& d_{S}^{U}(R,S) = \sqrt{ (\mathbf{p}^{R} - \mathbf{p}^{S})^{\top} \mathbf{B} (\mathbf{p}^{R} - \mathbf{p}^{S})}.\end{align*}

The distinction from UniFrac is particularly clear from the quadratic-form representation. UniFrac accumulates branch-length-weighted differences branch by branch, whereas koinetic encodes the full weighted phylogenetic graph in the evolutionary covariance matrix $\mathbf{B}$ and evaluates the complete community-difference vector jointly. Let $\delta _{i}^{U}=p_{i}^{R}-p_{i}^{S}$ denote the occurrence difference at node *i*. Then


\begin{align*} & \left[d_{S}^{U}(R,S)\right]^{2} = \sum_{i} B_{ii}(\delta_{i}^{U})^{2} + 2\sum_{i<j}B_{ij}\delta_{i}^{U}\delta_{j}^{U}. \end{align*}

The diagonal terms quantify node-specific occurrence differences under the phylogenetic leverage encoded by $\mathbf{B}$, whereas the off-diagonal terms allow differences at evolutionarily coupled nodes to contribute jointly through the Laplacian diffusion kernel. Consequently, concordant occurrence differences among related lineages can reinforce one another through shared phylogenetic diffusion paths instead of entering only as separate branch-wise turnover contributions. The same decomposition applies to weighted koinetic with $\delta _{i}^{W}=r_{i}^{R}-r_{i}^{S}$, yielding an analogous phylogenetic-diffusion interpretation for abundance differences. A more detailed interpretation of the koinos and kinetic components is provided in Supplementary Section 1.5.

In the toy example, the weighted koinetic distance between samples *R* and *S* is 1.86, whereas the unweighted koinetic distance is 3.60. They capture community-wide differences as lineage diffusion across the phylogenetic tree, extending beyond the lineage turnover measured by UniFrac.

### Leverage analysis

Phylogenetic leverage quantifies how strongly a node contributes to the overall pairwise dissimilarity between microbial communities, conditional on its position within the phylogenetic tree. It is defined as the contribution of a node to pairwise dissimilarity under a unit standardized abundance or prevalence difference at that node, with all other nodes held fixed.

For UniFrac-type metrics, node leverage is inherently local. Each taxon contributes to dissimilarity only through the branch connecting it to its immediate ancestor, and the magnitude of its influence is determined solely by the corresponding branch length. As a result, UniFrac leverage reflects branch-wise turnover and does not account for the broader clade structure or extended evolutionary context surrounding a node. In contrast, koinetic defines dissimilarity through a real, symmetric kernel matrix $\mathbf{B}=\mathbf{L}^{-1}$, where $\mathbf{L}$ is the graph Laplacian constructed from the phylogenetic tree. This formulation couples all nodes through shared ancestry and enables differences at one node to propagate along multiple evolutionary paths. From a leverage perspective, this implies that a node’s contribution to dissimilarity depends not only on its immediate branch length, but also on how it is embedded within the surrounding phylogenetic structure.

Under the quadratic-form representation of koinetic, this contribution corresponds to the node’s self-influence within the kernel matrix $\mathbf{B}$. In practice, leverage is computed via the eigendecomposition of the Laplacian matrix, with each node’s leverage determined by its associated spectral weights in the inverse Laplacian. This spectral implementation directly reflects how strongly perturbations at a given node propagate through the phylogenetic network. This perspective yields two key insights. First, nodes located in regions of the phylogenetic tree with many nearby evolutionary paths and closely related lineages—such as dense clades or extended shared ancestry—exert greater leverage, as differences at these nodes propagate through multiple connected routes. Conversely, nodes situated in sparsely connected or isolated regions of the tree tend to have limited leverage, contributing primarily through a small number of propagation pathways. Second, the distribution of leverage across spectral components provides a diagnostic view of how phylogenetic information is utilized by koinetic: leverage that is broadly distributed reflects integration of multiple evolutionary paths, whereas highly concentrated leverage indicates reliance on a limited subset of phylogenetic structure.

Therefore, leverage analysis offers a principled and interpretable quantification of how phylogenetic structure reweights node-level contributions to community dissimilarity under koinetic, while highlighting fundamental differences from branch-wise metrics such as UniFrac.

## Results

### Implementation

We developed and implemented koinetic in an open-source R package, GLaD (Graph Laplacian Distance), which provides efficient routines for distance computation and benchmarking. This package accepts phyloseq objects [35] as input, allowing direct integration with widely used microbiome analysis workflows. Users can specify the parameter *ρ * to customize the Laplacian kernel, controlling the strength of phylogenetic coupling encoded in the distance. By default, we recommend *ρ = 1*, which corresponds to fully leveraging the phylogenetic tree structure and, in our experience, performs well for unsupervised tasks such as ordination and exploratory community comparison. For supervised tasks, *ρ * may alternatively be treated as a tuning parameter to optimize sensitivity under specific signal structures. The resulting distance object is returned in the standard dist format, enabling seamless compatibility with vegan and other downstream packages. In addition to distance computation, GLaD implements the evaluation metrics used in the Dissimilarity Recommendation Score (DRS) framework, supporting reproducible benchmarking and method selection. The evaluation criteria and Borda rank aggregation used in the tournament plots are described in Supplementary Section 1.6. All computations of koinetic presented in the Results section were carried out using GLaD, and both the package source code and data used in this study are openly available at https://github.com/bioscinema/GLaD. This implementation ensures that koinetic is accessible, reproducible, and immediately applicable to microbiome research.

### Simulation study

We conducted a simulation study to characterize the group-separating signal captured by GLaD under controlled perturbations of phylogeny-guided OTU clusters. Rather than aiming to show that one dissimilarity is uniformly superior, the simulation was designed as an illustrative experiment in which a cluster of phylogenetically related OTUs was perturbed through changes in abundance, prevalence, or both, thereby inducing separation between groups. This setting reflects a common form of structured microbial variation for which tree-based distances are motivated. By comparing GLaD with UniFrac-based and non-phylogenetic alternatives under the same perturbation schemes, we examined whether incorporating both branch-length and local adjacency information improves the detection of phylogenetically localized community differences.

We implemented a realistic simulation framework using the throat microbiome dataset included in the GUniFrac package [17] as the reference data. We first fitted a SparseDOSSA2 template [36] to the empirical OTU table, so that the simulated communities retained key distributional features of real microbiome data, including sparsity, overdispersion, and taxon–taxon dependence. In the fitted SparseDOSSA2 model, let $A_{ij}$ denote the latent absolute abundance of OTU *j* in simulated sample *i*. For each OTU *j*, the baseline parameters $\pi _{j}$, $\mu _{j}$, and $\sigma _{j}^{2}$ are estimated by fitting SparseDOSSA2 to the empirical throat-microbiome reference OTU table. Here, $\pi _{j}$ denotes the fitted probability that OTU *j* is absent, whereas $\mu _{j}$ and $\sigma _{j}^{2}$ denote the fitted mean and variance, respectively, of the log absolute-abundance distribution conditional on that OTU being present. Thus, under the fitted baseline model,


(6) \begin{align*}& A_{ij}=0 \text{ with probability}\ \pi_{j}, \qquad \log A_{ij}\mid A_{ij}>0 \sim N(\mu_{j},\sigma_{j}^{2}).\end{align*}

Cross-OTU dependence is represented through the Gaussian-copula dependence structure learned from the same reference dataset. The latent abundances are then normalized to relative abundances as


(7) \begin{align*}& X_{ij} = \frac{A_{ij}}{\sum_{\ell=1}^{p}A_{i\ell}},\end{align*}

where *p* is the total number of OTUs. Conditional on the sample-specific sequencing depth $D_{i}$, the observed OTU counts are generated according to


(8) \begin{align*}& (C_{i1},\ldots,C_{ip}) \sim \mathrm{Multinomial}(D_{i};X_{i1},\ldots,X_{ip}).\end{align*}

Here, $D_{i}=\sum _{j=1}^{p} C_{ij}$ denotes the total sequencing depth (library size) of simulated sample *i*. We did not externally prespecify sequencing depths in our simulations. Instead, sample-specific values of $D_{i}$ were generated from the sequencing-depth distribution estimated by the fitted SparseDOSSA2 template from the empirical throat-microbiome reference dataset. Hence, the fitted reference template supplies the baseline feature-specific parameters $(\pi _{j},\mu _{j},\sigma _{j}^{2})$, the cross-feature dependence structure, and the sequencing-depth distribution used to generate the simulated communities. Following prior microbiome association-test simulations [37–39], to construct phylogenetically localized signals, we computed the patristic distance matrix from the reference phylogenetic tree and applied partitioning around medoids to cluster OTUs according to their evolutionary dissimilarity, with *K = 2, 4, 6, 8, 10,* or *12* clusters. OTUs within the same cluster were therefore more phylogenetically similar to each other than to OTUs assigned to other clusters. For each value of *K*, we iteratively selected the *k*th cluster, *k = 1,… ,K*, as the signal cluster driving group separation, and treated all OTUs in that cluster as spike features in SparseDOSSA2. This clustering step defines a phylogenetically localized support for the subsequent perturbations. Although SparseDOSSA2 modifies feature-specific marginal abundance and/or occurrence parameters, all spike features within a given setting belong to the same phylogenetic cluster. The resulting abundance or occurrence differences are therefore aligned with the fixed phylogenetic structure, allowing the simulation to assess whether different dissimilarities exploit that structure differently. The binary group indicator was generated as $G_{i}\sim \mathrm{Bernoulli}(0.5)$, defining a reference group ($G_{i}=0$) and a perturbed group ($G_{i}=1$). Importantly, although $G_{i}$ was generated marginally from this Bernoulli distribution, the microbial profiles were subsequently generated conditionally on $G_{i}$: for OTUs in the selected signal cluster, $G_{i}$ entered the SparseDOSSA2 spike-in model as the sample-level covariate governing the perturbation, whereas nonsignal OTUs retained their fitted baseline parameters.

Specifically, for a signal OTU *j*, an abundance perturbation modifies its fitted baseline mean $\mu _{j}$ while leaving the fitted conditional variance $\sigma _{j}^{2}$ unchanged, according to


(9) \begin{align*}& \mu_{ij}=\mu_{j}+G_{i}\beta_{\mathrm{abund}}, \qquad \log A_{ij}\mid A_{ij}>0,G_{i}\sim N(\mu_{j}+G_{i}\beta_{\mathrm{abund}},\sigma_{j}^{2}),\end{align*}

so that $\beta _{\mathrm{abund}}$ is the log fold change in positive latent absolute abundance between $G_{i}=1$ and $G_{i}=0$, and $\exp (\beta _{\mathrm{abund}})$ is the corresponding fold change. A prevalence perturbation modifies its fitted baseline absence probability $\pi _{j}$ through


(10) \begin{align*}& \log\left(\frac{1-\pi_{ij}}{\pi_{ij}}\right) = \log\left(\frac{1-\pi_{j}}{\pi_{j}}\right) +G_{i}\beta_{\mathrm{prev}},\end{align*}

where $\pi _{ij}$ is the absence probability, so that $\beta _{\mathrm{prev}}$ is the log odds ratio for OTU presence and $\exp (\beta _{\mathrm{prev}})$ is the corresponding odds ratio. Thus, the effect-size parameters quantify group-dependent changes in the microbial features conditional on $G_{i}$; they do not parameterize the marginal Bernoulli distribution of $G_{i}$.

We considered a single nominal effect size $\beta \in \{0,0.6,0.8,1.2,1.6,2.0\}$ and mapped it to the component-specific coefficients according to the signal mode:


(11) \begin{align*}& (\beta_{\mathrm{abund}},\beta_{\mathrm{prev}}) = \begin{cases} (\beta,0), & \text{abundance only},\\ (0,\beta), & \text{prevalence only},\\ (\beta,\beta), & \text{combined abundance and prevalence}. \end{cases}\end{align*}

Accordingly, under the abundance-only mode, signal OTUs differ between groups only in their positive abundance distributions; under the prevalence-only mode, they differ only in their occurrence probabilities; and under the combined mode, the same nominal coefficient is applied on the two distinct link scales (log fold change for abundance and log odds ratio for prevalence). The common numerical value in the combined mode is therefore a simulation-control parameter rather than a claim that the two component effects are directly comparable on a common biological scale. When *β =0*, both component-specific coefficients are zero and no group-dependent perturbation is introduced; this setting was used to estimate empirical type I error. Positive values of *β * induce progressively stronger group separation and were used to evaluate power. The fitted simulation model and perturbation mechanisms are described in Supplementary Section 2.1; the complementary cluster-specific, abundance-weighted, and largest-cluster summaries are explained in Supplementary Section 2.2.

For each simulation setting, we generated 100 independent replicates. For each simulated OTU table with the corresponding group variable, we computed four variants of GLaD, including weighted and unweighted versions with *ρ = 0.5* or *ρ = 1*, three UniFrac-based distances, including weighted UniFrac, unweighted UniFrac, and generalized UniFrac, and four commonly used non-phylogenetic dissimilarities: Euclidean distance, Bray–Curtis dissimilarity, Aitchison distance, and Jaccard distance. These non-phylogenetic benchmarks were included to represent complementary distance properties: Euclidean distance as a standard Euclidean metric, Bray–Curtis as a widely used ecological dissimilarity, Aitchison distance as a Euclidean distance that accounts for compositionality, and Jaccard distance as a presence–absence measure. For each distance matrix, we used adonis2() to test its association with the binary group variable, with a significance level of *0.05*. The rejection proportion across 100 replicates was interpreted as the empirical type I error when the effect size was *0*, and as empirical power when the effect size was positive. In the main text, we present the power curves for the largest signal cluster, defined as the cluster containing the greatest number of OTUs, under *K = 2, 4, 6,* and *8*. For each of these settings, we show how empirical power changed with increasing effect size under all three signal modes: abundance, prevalence, and both abundance and prevalence. Across these displayed scenarios, the empirical type I error of all dissimilarities fluctuated around the nominal significance level and did not show a systematic pattern of concern; these results are therefore reported in Supplementary Section 2.3 (Table S1). Additional simulation results are provided in the same section: Fig. S1 summarizes abundance-weighted power, Figs S2–S5 show cluster-specific power, and Tables S3–S6 describe the corresponding signal clusters.

The empirical power curves in Fig. 3 revealed distinct signal profiles across the three perturbation modes. When group separation was driven by abundance changes, weighted GLaD, generalized UniFrac, and weighted UniFrac were consistently among the most powerful methods across different levels of phylogenetic clustering. This pattern is expected because, under this signal mode, the phylogenetic branch-length information and the associated abundance variation jointly provide sufficient information to separate the two groups. In contrast, unweighted GLaD and unweighted UniFrac had little power, because the data-generating mechanism did not induce group differences in presence–absence patterns along edges or clades. When the signal was introduced through prevalence changes, the advantage of unweighted GLaD with *ρ = 0.5* became increasingly apparent as the signal cluster became more localized. Under this signal mode, each selected OTU differs between groups only in its occurrence probability, but the selected OTUs belong to the same phylogenetic cluster, so the induced occurrence-difference vector has phylogenetically localized support. Unweighted UniFrac summarizes these differences through branch-length-weighted turnover accumulated over individual branches. In contrast, unweighted GLaD evaluates the complete occurrence-difference vector jointly through $\mathbf{B}$: its diagonal terms capture node-specific differences, while its off-diagonal terms couple differences at evolutionarily related nodes through shared phylogenetic diffusion paths. When related taxa exhibit concordant occurrence shifts, these cross-lineage terms reinforce the resulting between-community distance. This difference in how the fixed phylogeny is used provides a mechanism for the higher power of unweighted GLaD in these settings. Unweighted GLaD with *ρ = 1* achieved the highest power for coarse clusters, but its advantage diminished for finer clusters, eventually falling below unweighted GLaD with *ρ = 0.5*, unweighted UniFrac, and Aitchison distance. This behavior is consistent with the role of *ρ *: *ρ = 1* places stronger emphasis on global tree connectivity and therefore benefits from more complete topological signals, whereas *ρ = 0.5* provides a more balanced weighting when the signal occupies only a restricted portion of the tree. Finally, under the combined abundance-and-prevalence mode, weighted GLaD and generalized UniFrac again showed the strongest performance, resembling the abundance-only setting. This is likely because SparseDOSSA2 applied the same effect size to both abundance and prevalence perturbations, and in these simulated communities the abundance component contributed substantially more variation than the prevalence component.

**Figure 3:**
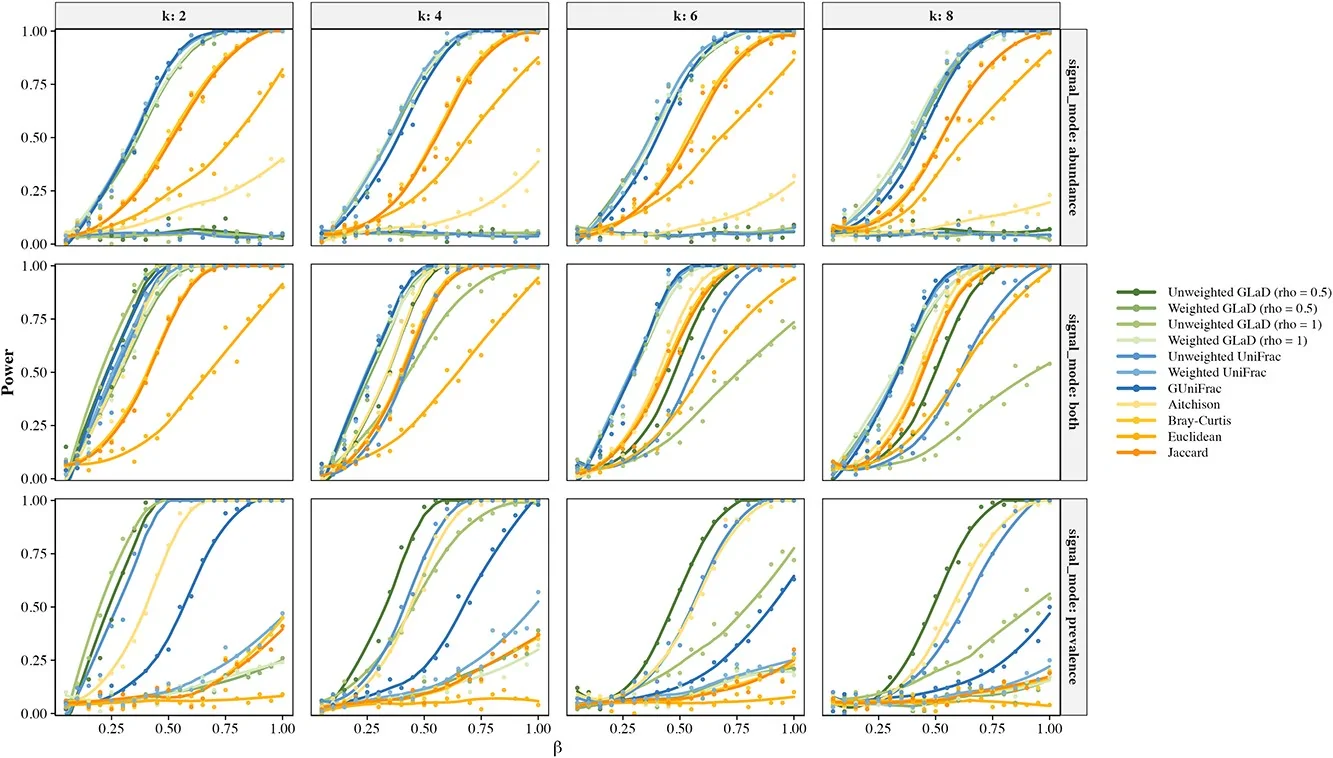
Empirical power based on the largest signal cluster under each simulation setting. For each value of *k*, signal mode, and effect size *β *, the signal cluster with the largest number of OTUs was selected, and power was computed as the rejection rate within that selected cluster. Points represent empirical power values, while solid curves show smoothed trends across *β *. Panels correspond to different combinations of *k* and signal mode.

Overall, the simulation study supports two main findings. First, when group separation was driven by abundance perturbations, either alone or together with prevalence perturbations, the relevant information was largely reflected in abundance variation and further strengthened by phylogenetic structure. In these settings, weighted GLaD, weighted UniFrac, and generalized UniFrac showed comparable and consistently strong performance, suggesting that branch-length-weighted abundance information is sufficient to capture this form of group separation. Second, under phylogenetically localized prevalence perturbations, unweighted GLaD frequently achieved higher power because it evaluates the full occurrence-difference vector jointly through the Laplacian-derived evolutionary covariance matrix, rather than accumulating only branch-wise turnover. The off-diagonal terms in this quadratic form allow concordant occurrence differences among evolutionarily related taxa to reinforce one another through phylogenetic diffusion. The observed gain therefore reflects how GLaD uses the fixed phylogenetic structure when the prevalence signal is localized on the tree, rather than a general advantage for prevalence perturbations *per se*. The optimal choice of *ρ * still depended on the granularity of the signal cluster: larger values of *ρ * were advantageous when the signal involved coarser phylogenetic clusters, whereas *ρ = 0.5* was more robust when the signal was localized within finer clusters.

### Real data analysis

To further assess the theoretical and practical advantages of koinetic, we conducted a three-part comparative analysis against phylogeny-aware dissimilarities such as unweighted, weighted, and generalized UniFrac and other commonly used dissimilarities such as Euclidean, Aitchison, Bray–Curtis, and Jaccard. Each component isolates a key criterion in dissimilarity assessment: (i) the leverage each OTU exerts on community distances; (ii) the Euclidean property [31, 33]; and (iii) the performance in downstream inference tasks such as PERMANOVA [40] and MiRKAT [41]. This sequence—from pairwise definition to statistical utility—allows for a systematic evaluation of the strengths and weaknesses of each measure and for quantifying the improvements achieved by the koinetic formulation across diverse analytical settings. We carried out the comparative analyses on eight real-world microbiome datasets, spanning pancreatic cancer, IBD, and metabolic disease–associated oral microbiomes, with dataset descriptions in Supplementary Section 3.1 and bioinformatics processing details in Supplementary Section 3.2.

We first examine the leverage exerted by each OTU on pairwise dissimilarities under UniFrac and koinetic with *ρ = 1* using pancreatic tumor microbiome data (Fig. 4, left). While koinetic exhibits a broad spectrum of leverage values across taxa, UniFrac yields a sharply concentrated distribution. This contrast reflects a fundamental difference in how phylogenetic information shapes OTU contributions: dissimilarities like Euclidean assign uniform weights to all features, whereas both koinetic and UniFrac encode phylogeny-dependent weights. However, UniFrac’s reliance on edge-specific uniqueness localizes leverage to OTUs with long terminal branches, thus rendering its leverage values narrowly distributed. In contrast, koinetic incorporates both branch length and topological context through the graph Laplacian, allowing nearby OTUs to share and diffuse signal along the tree. As a result, OTUs with complex neighborhood structures or long paths to their most recent ancestors exert higher leverage, producing a more balanced and structurally expressive weighting scheme. The right panel of Fig. 4 illustrates how parameter *ρ * modulates leverage structure in koinetic. Conceptually, *ρ = 1* fully incorporates hierarchical relationships, whereas *ρ = 0* ignores phylogenetic information. As shown, when *ρ = 0.1*, koinetic recovers a leverage profile nearly identical to that of UniFrac, narrow and concentrated, indicating limited diffusion of phylogenetic context. As *ρ * increases to 0.5, leverage begins to diversify, reflecting moderate engagement of the tree structure. At *ρ = 0.9*, the leverage curve closely aligns with the *ρ = 1* baseline, capturing extensive long-range interactions shaped by the full topology. These patterns confirm that phylogenetic information meaningfully distributes influence across taxa and, when properly incorporated, offers an additional resolution beyond local branch uniqueness.

**Figure 4:**
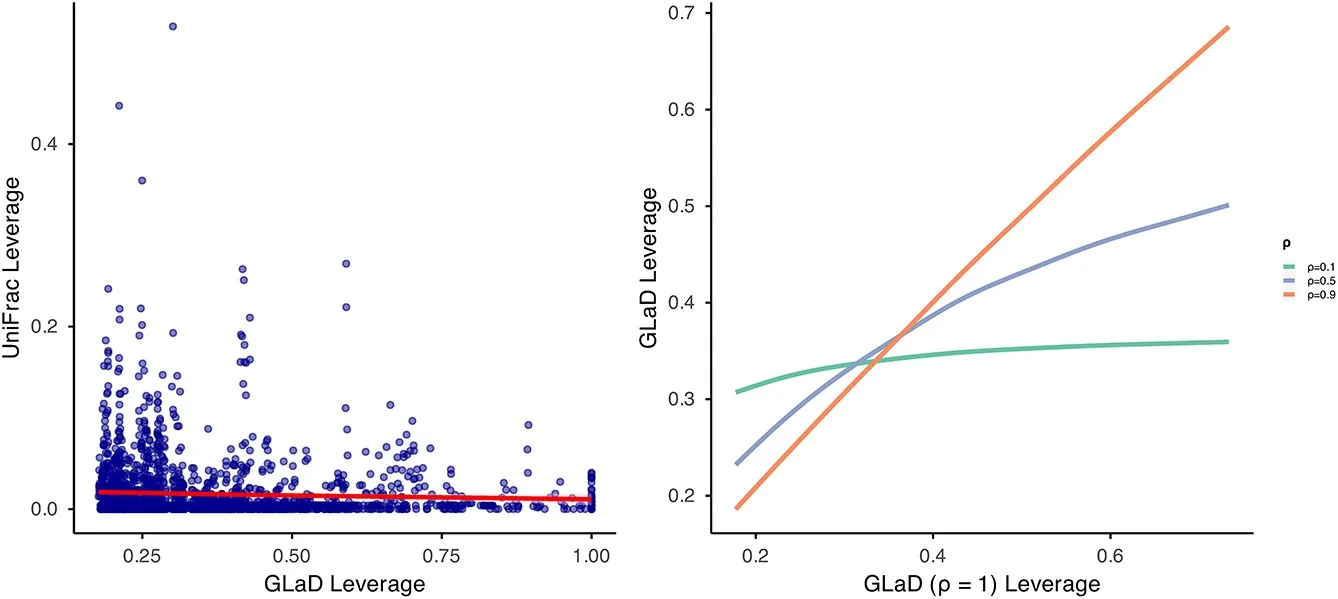
Left: comparison between UniFrac leverage and GLaD leverage (*ρ = 1*) across taxa. Right: smoothed relationships between GLaD leverage at *ρ = 1* and corresponding leverage values under varying phylogenetic autocorrelation (*ρ = 0.1, 0.5, 0.9*), illustrating monotonic distortion under decreasing phylogenetic resolution.

Table 1 summarizes how consistently each dissimilarity measure satisfies the Euclidean property across eight microbiome datasets. Consistent with the theoretical construction of GLaD, all four GLaD variants were Euclidean in every dataset considered. The standard Euclidean, Jaccard, and Aitchison distances also satisfied the Euclidean property across all datasets, whereas Bray–Curtis dissimilarity failed to do so consistently, in line with its known behavior as a semi-metric that may violate metric properties such as the triangle inequality. Among the UniFrac-based distances, weighted UniFrac and generalized UniFrac failed to satisfy the Euclidean property in several datasets, indicating that their Euclidean behavior is data-dependent. In contrast, unweighted UniFrac was Euclidean for all eight datasets examined here, although this should be viewed as a dataset-specific empirical result rather than a general theoretical property; unweighted UniFrac is not universally Euclidean, and counterexamples have been reported previously [18].

**Table 1:** Euclidean-property assessment of dissimilarity matrices across benchmark data sets. Note that ✓means the dissimilarity satisfies the Euclidean property for the specific dataset while *× * means not.

Method	COVID gut	HIV gut	IBD 16S	IBD WGS	Oral 16S	Oral WGS	Tumor	Pig gut
Weighted GLAD (*ρ = 0.5*)	✓	✓	✓	✓	✓	✓	✓	✓
Unweighted GLAD (*ρ = 0.5*)	✓	✓	✓	✓	✓	✓	✓	✓
Weighted GLAD (*ρ = 1*)	✓	✓	✓	✓	✓	✓	✓	✓
Unweighted GLAD (*ρ = 1*)	✓	✓	✓	✓	✓	✓	✓	✓
Weighted UniFrac	*× *	*× *	*× *	*× *	*× *	*× *	*× *	*× *
Unweighted UniFrac	✓	✓	✓	✓	✓	✓	✓	✓
Generalized UniFrac	✓	*× *	*× *	*× *	*× *	*× *	✓	✓
Euclidean	✓	✓	✓	✓	✓	✓	✓	✓
Jaccard	✓	✓	✓	✓	✓	✓	✓	✓
Bray–Curtis	*× *	*× *	*× *	*× *	*× *	*× *	✓	*× *
Aitchison	✓	✓	✓	✓	✓	✓	✓	✓

Supplementary Section 3.3 (Tables S7–S9) reports the numerical embedding and clustering diagnostics, and Supplementary Section 3.4 provides two graphical views of these benchmarks. The radar plot (Supplementary Fig. S6) summarizes the same eight-dataset Euclidean-property assessment as Table 1, showing the number of datasets for which each dissimilarity matrix satisfies the Euclidean property. The tournament plots (Supplementary Figs S7–S9) display the ranks of the candidate dissimilarities across six evaluation criteria and the aggregate Borda score for the IBD 16S and WGS cohorts (Fig. S7), COVID–Gut and HIV–Gut (Fig. S8), and pancreatic tumor and Pig–Gut datasets (Fig. S9). These plots make the criterion-specific rankings and variation across datasets visible alongside the tabulated Euclidean-property results.

Figure 5 displays the downstream evolutionary covariance strength of each *β *-diversity measure with host phenotypes across eight datasets, evaluated using two complementary statistical frameworks: MiRKAT $R^{2}$ (left panel) and PERMANOVA *F*-statistic (right panel). Supplementary Section 3.5 (Tables S10–S11) provides additional numerical results from these analyses. Bubble size and color intensity encode effect magnitude, with larger, warmer-toned circles indicating stronger evolutionary covariances. Across nearly all datasets and both tests, koinetic variants consistently achieve the top ranks. In particular, Unweighted koinetic (*ρ = 1*) achieves the highest test statistics in most settings, closely followed by Weighted koinetic (*ρ = 1*). Interestingly, the weighted and unweighted versions of koinetic appear to be complementary: in datasets where unweighted koinetic performs slightly less well such as the Pig Gut or IBD-WGS cohorts, its weighted counterpart compensates, suggesting that incorporating abundance differences can help in datasets with denser or more complex compositional structure. This flexibility highlights koinetic’s adaptability across biological contexts and sequencing platforms. Generalized UniFrac shows moderate, stable performance across all datasets but never reaches the top in either metric. This is consistent with its design as a compromise between presence–absence and abundance-based weighting. However, its geometry remains partially non-Euclidean, which may explain its limited power compared with koinetic [42]. In contrast, standard choices like unweighted UniFrac and weighted UniFrac generally yield weaker test statistics, particularly in the HIV and IBD datasets. These methods struggle to capture subtle compositional shifts, especially when the distance structure is distorted or ecological gradients are nonlinear [43, 44]. Performance also varies by dataset. In the ORIGINS Oral Microbiome, effect sizes are generally lower across all distances, likely due to the limited number of OTUs and taxonomic breadth, which constrains ecological variation. In contrast, the HIV Gut Microbiome yields strong evolutionary covariances, especially for koinetic and Bray–Curtis. This reflects the substantial microbial restructuring that can occur due to antiretroviral therapy, inflammation, and immune dysregulation—processes that fundamentally reshape community composition [16, 45]. Taken together, Fig. 5 highlights the statistical power and robustness of koinetic in detecting microbiome–trait evolutionary covariances. Its consistently high performance across disease contexts, sequencing types, and study populations demonstrates its generalizability, while its complementarity between weighted and unweighted forms provides flexibility in adapting to dataset-specific sparsity and signal characteristics.

**Figure 5:**
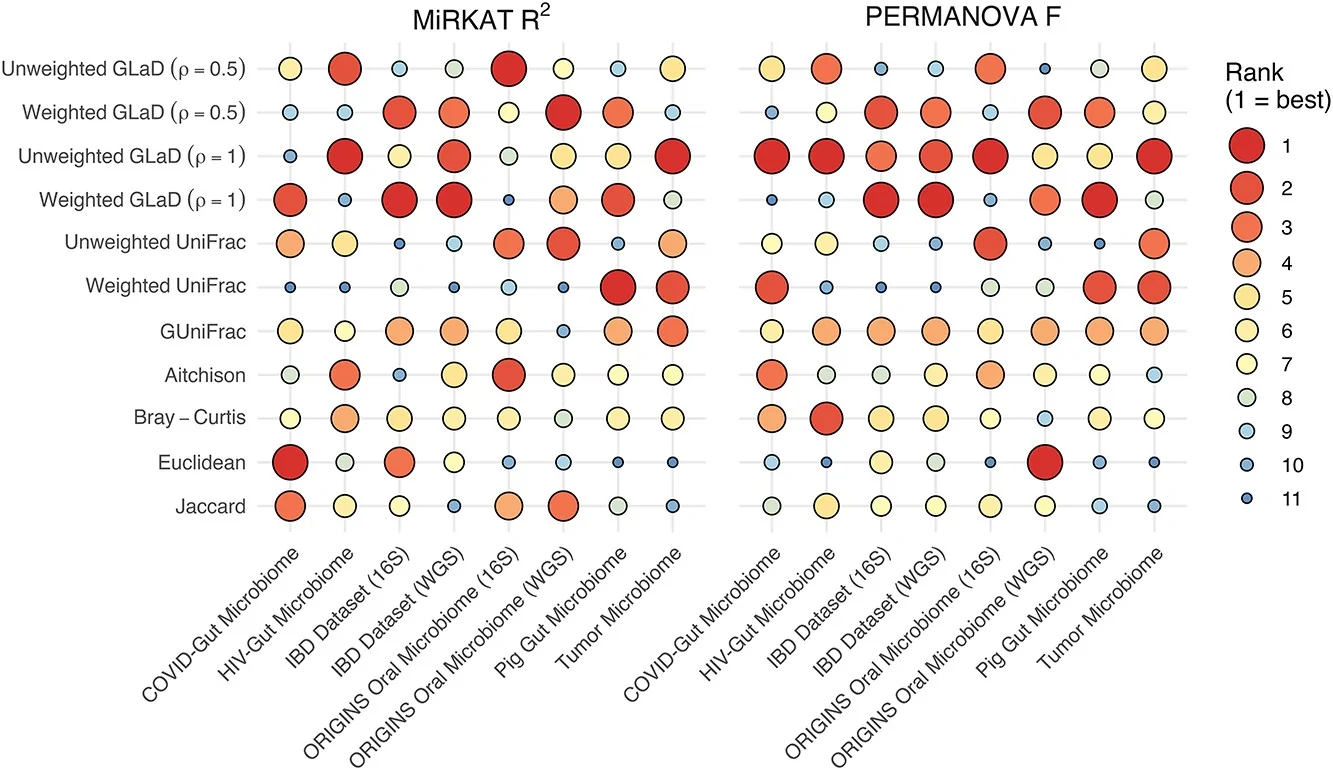
Evolutionary covariance strength of distance measures with host covariates. Bubble sizes and colors encode within-dataset ranks of the test statistics from two complementary analyses: MiRKAT $R^{2}$ (left grid) and PERMANOVA *F* (right grid). The rows correspond to *β *-diversity metrics; columns to the eight microbiome datasets. The warmer hues and larger circles mark higher-ranked test statistics. The four koinetic variants (*ρ =0.5* or 1, weighted or unweighted) yield the highest PERMANOVA *F* values in every dataset and rank first for MiRKAT $R^{2}$ in four of the eight datasets, highlighting their overall power to detect microbiome–trait relationships.

Above all, these empirical evaluations demonstrate that koinetic not only satisfies critical geometric properties across diverse microbiome datasets, but also achieves superior performance in both structure preservation and evolutionary covariance testing. Unlike traditional metrics such as UniFrac, Jaccard, or Bray–Curtis, koinetic maintains Euclidean embeddability, adapts flexibly to data sparsity and sequencing modality, and consistently captures biologically meaningful variation. The complementarity of its weighted and unweighted forms further enhances its robustness across ecological and clinical contexts. These findings establish koinetic as a general-purpose, statistically reliable dissimilarity measure for phylogeny-aware microbiome analysis.

## Discussion

In this paper, we introduced koinetic, a graph Laplacian-based framework for phylogeny-aware dissimilarity analysis that jointly incorporates tree topology and branch lengths while ensuring Euclidean compatibility for downstream statistical modeling [18]. Our simulation study further showed that koinetic captures phylogenetically localized group separation as intended: weighted koinetic performs comparably with abundance-sensitive UniFrac variants under abundance-driven signals, whereas unweighted GLaD can gain sensitivity for phylogenetically localized prevalence signals by evaluating occurrence differences jointly through the fixed phylogenetic diffusion structure.

Koinetic has also been validated across diverse microbiome contexts, including infection-related human gut cohorts (IBD and HIV), cancer-associated microbiomes (pancreatic tumor), metabolic disease–associated oral microbiomes, and animal models (pig gut). This breadth of validation provides empirical evidence that the method performs consistently across the datasets examined in this study, encompassing multiple host species, body sites, and sequencing platforms (16S and WGS). Such generality makes koinetic a practical choice for investigators seeking a phylogeny-aware distance that can adapt to varied ecological and biomedical settings.

The innovation of koinetic lies in its ability to move beyond individual-taxon testing and classical *β *-diversity by measuring differences at the whole-community level. The term “koinos” emphasizes collective evaluation, while “kinetic” highlights how differences propagate dynamically through ecological and phylogenetic relationships. Unlike presence–absence or abundance-based distances that treat taxa independently, koinetic leverages a Laplacian propagation kernel to weight differences according to shared phylogeny, capturing how changes in one lineage resonate through its neighbors. This community-wide, graph-aware view aligns with the biological reality of microbial systems, where taxa act not in isolation but as interconnected, evolutionary assemblies. This distinction is particularly relevant when occurrence differences are concentrated among phylogenetically related taxa. UniFrac primarily accumulates the resulting lineage turnover branch by branch, whereas koinetic applies the Laplacian-derived covariance to the complete occurrence-difference vector, allowing related lineage differences to contribute jointly through shared diffusion paths. Thus, even taxon-specific marginal prevalence shifts can form a stronger community-level signal when they are aligned with the phylogenetic coupling encoded by koinetic. While UniFrac remains a cornerstone for quantifying the fraction of unique branch lengths between communities, it fundamentally treats differences as static and branch-constrained. In practice, UniFrac identifies where two communities diverge in their tree coverage, whereas koinetic reveals how those differences propagate across related clades. These perspectives are complementary: UniFrac offers a parsimonious view of divergence, while koinetic provides a dynamic and biologically realistic picture of community-wide differentiation.

Like any tree-based method, koinetic depends on the accuracy of the input phylogeny and can incur computational cost in large-scale studies. Errors in sequence alignment or tree construction may propagate through the Laplacian kernel, and dense datasets may require efficient numerical solvers or low-rank approximations. Therefore, routine assessment of tree quality and sensitivity analysis across alternative topologies is recommended. Future work may also integrate ecological networks or temporal structure to further extend the scope of Laplacian-based community analysis.

From a broader perspective, Koinetic enhances our ability to detect fine-scale community differences, interpret ecological gradients, and evaluate group separation in a phylogenetically coherent manner. Importantly, the underlying spectral formulation is not confined to microbiome studies but may extend naturally to other domains where data are structured by evolutionary trees, such as molecular evolution, trait genomics, and functional phylogenomics. By aligning methodological rigor with ecological interpretability, Koinetic provides a unified and extensible approach for analyzing tree-structured biological data, offering a practical tool for linking microbial community variation to host, environment, and evolutionary processes.

**Key Points**

- Koinetic advances microbial community comparison by jointly evaluating differences among evolutionarily related lineages through a graph Laplacian kernel integrating tree topology and branch lengths.
- The framework guarantees Euclidean validity, providing a consistent geometric basis for visualization and kernel-based inference without *post hoc* geometric corrections.
- Simulations demonstrate power gains for unweighted Koinetic under phylogenetically localized prevalence shifts and competitive performance for weighted Koinetic against weighted and generalized UniFrac under abundance-driven signals.
- Eight microbiome datasets spanning diverse body sites, disease contexts, and microbial profiling modalities demonstrate broad applicability and strong performance in PERMANOVA and MiRKAT analyses of microbiome–host associations.

## Supplementary Material

Koinetic_Supplementary_swift

## Data Availability

The “GLaD” R package, data, and R scripts used for the analyses are available at https://github.com/bioscinema/GLaD.
