# Supplementary material for: Koinetic: a graph-based distance as a new ecological metric for microbial community differential analysis
Source: Brief Bioinform. 2026 Nov 15;27(6):bbag632. doi: 10.1093/bib/bbag632 (PMC13770251; doi:10.1093/bib/bbag632)
Supplement: Supplementary file 1 [file koinetic_supplementary_swift.pdf]

Supplementary Materials of "Koinetic: A Graph-based Distance as a New Ecological Metric for Microbial Community Differential Analysis"

Zihan Zhu[1], Yiqian Zhang[2,3], Ruitao Liu[1], Yi Duan[4,5], Wenhao Li[6], Zijing Huang[4], Xinhua Chen[7], Liangliang Zhang[1,8*]

[1]Department of Population and Quantitative Health Sciences, School of Medicine, Case Western Reserve University, 10900 Euclid Ave., Cleveland, 44106, OH, USA.
[2]Division of Biostatistics, College of Public Health, Ohio State University, 1841 Neil Ave., Columbus, 43210, OH, USA.
[3]Department of Statistics, College of Arts and Sciences, The Ohio State University, 1958 Neil Ave., Columbus, 43210, OH, USA.
[4]State Key Laboratory of Immune Response and Immunotherapy, Department of Infectious Diseases, The First Affiliated Hospital of USTC, Center for Advanced Interdisciplinary Science and Biomedicine of IHM, Division of Life Sciences and Medicine, University of Science and Technology of China, Hefei, Anhui, China.
[5]Key Laboratory of Anhui Province for Emerging and Reemerging Infectious Diseases, Hefei, Anhui, China.
[6]Department of Biostatistics, The University of Texas MD Anderson Cancer Center, 7007 Bertner Ave., Houston, 77030, TX, USA.
[7]Department of Hepatobiliary-Pancreatic Surgery, The First Affiliated Hospital, Zhejiang University School of Medicine, Hangzhou, 310000, Zhejiang, China.
[8]Case Comprehensive Cancer Center, 2103 Cornell Road, Cleveland, 44106, OH, USA.

1 Mathematical and Computational Details

1.1 A Brief Review of the Euclidean Property

Let $\boldsymbol{D} = (D_{ij})_{i,j=1}^{N}$ be a symmetric dissimilarity matrix for N microbial communities, with $D_{ii} = 0$. The dissimilarity is said to possess the Euclidean property if there exist points $\boldsymbol{x}_1, \ldots, \boldsymbol{x}_N \in \mathbb{R}^m$, for some $m \le N - 1$, such that

$$D_{ij}^2 = \|\boldsymbol{x}_i - \boldsymbol{x}_j\|_2^2, \qquad i, j = 1, \ldots, N. \tag{1}$$

Thus, a Euclidean dissimilarity can be represented exactly as an ordinary Euclidean distance without geometric distortion.

The Euclidean property can be characterized through the Gower-centered matrix (1). Let

$$\boldsymbol{J} = \boldsymbol{I}_N - \frac{1}{N}\boldsymbol{1}_N\boldsymbol{1}_N^\top \tag{2}$$

be the centering matrix and let $\boldsymbol{D}^{(2)} = (D_{ij}^2)_{i,j=1}^{N}$. The corresponding centered Gram matrix is

$$\boldsymbol{G} = -\frac{1}{2}\boldsymbol{J}\boldsymbol{D}^{(2)}\boldsymbol{J}. \tag{3}$$

The dissimilarity matrix $\boldsymbol{D}$ is Euclidean if and only if $\boldsymbol{G}$ is positive semidefinite. Equivalently, the squared dissimilarity matrix is conditionally negative definite, meaning that

$$\boldsymbol{c}^\top \boldsymbol{D}^{(2)}\boldsymbol{c} \le 0 \tag{4}$$

for every $\boldsymbol{c} \in \mathbb{R}^N$ satisfying $\boldsymbol{1}_N^\top \boldsymbol{c} = 0$ (1, 2). When this condition holds, the eigendecomposition

$$\boldsymbol{G} = \boldsymbol{Q}\boldsymbol{\Lambda}\boldsymbol{Q}^\top \tag{5}$$

contains only nonnegative eigenvalues, and the rows of $\boldsymbol{Q}\boldsymbol{\Lambda}^{1/2}$ provide Euclidean coordinates whose pairwise distances reproduce $\boldsymbol{D}$.

This property is relevant to PERMANOVA even though PERMANOVA can be computed for a non-Euclidean dissimilarity matrix (3). Let $\boldsymbol{H}$ denote the projection matrix associated with the covariates included in the model, after separating the intercept contribution, and let r denote the corresponding model degrees of freedom. A distance-based pseudo-F statistic can be written as

$$F = \frac{\operatorname{tr}(\boldsymbol{H}\boldsymbol{G}\boldsymbol{H})/r}{\operatorname{tr}\{(\boldsymbol{I}_N - \boldsymbol{H})\boldsymbol{G}(\boldsymbol{I}_N - \boldsymbol{H})\}/(N - r - 1)}. \tag{6}$$

If $\boldsymbol{G}$ is positive semidefinite, it is the Gram matrix of an ordinary Euclidean configuration. The numerator and denominator of (6) can then be interpreted as explained and residual variation in that Euclidean coordinate space.

By contrast, a non-Euclidean dissimilarity produces one or more negative eigenvalues. Writing $\boldsymbol{G} = \sum_{k=1}^{N} \lambda_k \boldsymbol{q}_k \boldsymbol{q}_k^\top$, the model sum of squares satisfies

$$\operatorname{tr}(\boldsymbol{H}\boldsymbol{G}\boldsymbol{H}) = \sum_{k=1}^{N} \lambda_k \|\boldsymbol{H}\boldsymbol{q}_k\|_2^2. \tag{7}$$

Hence, eigencomponents with $\lambda_k < 0$ make negative contributions to the quantity interpreted as explained variation. Analogous negative contributions can also occur in the residual component. The resulting pseudo-F statistic remains numerically computable and its permutation distribution can still be evaluated, but it no longer corresponds to a conventional nonnegative decomposition of Euclidean variation. Moreover, when negative eigencomponents are aligned with the model space, they can attenuate the estimated between-group separation and reduce power.

This distinction explains why implementations such as `adonis2()` allow non-Euclidean dissimilarities but also provide the Lingoes and Cailliez corrections. These procedures add constants to the dissimilarities, or to their squared values, so that the corrected Gower-centered matrix is positive semidefinite. Such corrections make a subsequent Euclidean representation possible, but they also alter the original dissimilarities. A distance that is Euclidean by construction does not require this additional modification.

The practical advantage of the Euclidean property in the present study is therefore not that it is required for PERMANOVA to run. Rather, it ensures that the PERMANOVA sums of squares admit a valid Euclidean variance interpretation, avoids distortions caused by negative eigenvalues, and permits the same dissimilarity to be used directly in downstream procedures such as principal coordinate analysis and positive semidefinite kernel methods. GLaD was designed to retain these geometric properties while incorporating phylogenetic information.

1.2 Definition of GLaD

In this subsection, we formally define GLaD. We represent the phylogeny as a weighted binary tree whose leaf nodes represent taxa and whose edge weights represent branch lengths. Let $G = (V, E)$ denote this tree, where $V = \{v_1, \cdots, v_N\}$ is the set of all leaf and internal nodes and $E = \{e_1, \cdots, e_M\}$ is the set of edges. Let $\mathbf{A} = [a_{ij}]$ be the evolutionary proximity matrix,

$$a_{ij} = \begin{cases} 0, (i,j) \notin E \\ w_{ij}, (i,j) \in E \end{cases} \tag{8}$$

where w_{ij} is the weight of edge (i, j). We assume $w_{ij} > 0$. However, due to unresolved polytomies or lack of evolutionary divergence (4, 5), there might be zero-length branches. While such branches are biologically interpretable, they introduce technical complications in our graph-based formulation. If a branch has length zero, the corresponding edge weight is zero. Retaining such edges preserves additional nodes but contributes no weight to the graph structure. This further induces rank deficiency in $\mathbf{A}$. For theoretical clarity and numerical stability, zero-length branches are removed when constructing $\mathbf{A}$. This operation is justified by the theoretical foundation of phylogenetically weighted distances such as UniFrac. From the perspective of any branch-length–weighted integral, a zero-length edge is equivalent to an edge contraction, meaning that the child node can be merged into its parent without altering any phylogenetically weighted quantity (6, 7). In matrix terms, this corresponds to removing rows and columns in $\mathbf{A}$ that contain only zeros, thereby refining the tree to

its non-degenerate backbone. Importantly, this operation does not discard abundance information, because the abundance of each tip is propagated along its entire ancestral path toward the root. Therefore, even if a terminal branch has zero length, its abundance is still accumulated to the nearest ancestor with positive branch length.

We treat the phylogenetic tree as a weighted undirected graph, with nodes representing taxa and internal ancestors and edge weights representing branch lengths. Its graph Laplacian is $\mathbf{L} = \mathbf{D} - \mathbf{A}$. For a connected tree, $\mathbf{L}$ is symmetric and positive semidefinite (PSD), with one zero eigenvalue and all remaining eigenvalues positive. We introduce $\rho \in [0, 1)$ as an evolutionary autocorrelation coefficient that controls the strength of phylogenetic coupling. Higher ρ values indicate stronger coupling between nearby nodes on the tree. We then define the phylogenetic graph Laplacian as

$$\mathbf{L}_P = \mathbf{D} - \rho\mathbf{A}. \tag{9}$$

For $0 \leq \rho < 1$ and strictly positive node degrees, this matrix is real, symmetric, and positive definite.

For $0 \leq \rho < 1$, let $\mathbf{B} = \mathbf{L}_P^{-1}$ be the positive definite evolutionary covariance matrix among the OTUs. The case $\rho = 1$ requires the separate spectral construction in Section 1.3. Following (8), the distance between two samples can be expressed as

$$d_S(r, s) = \sqrt{(\mathbf{p}_r - \mathbf{p}_s)'\mathbf{B}(\mathbf{p}_r - \mathbf{p}_s)} \tag{10}$$

where $\mathbf{p}_r$ is the relative-abundance or presence–absence vector used to calculate the weighted or unweighted distance, respectively. We refer to this dissimilarity between microbial communities, constructed via the Laplacian-informed evolutionary covariance matrix $\mathbf{B}$, as the Koinos Kinetic Distance (koinetic).

Based on the construction, if $\mathbf{B}$ is PSD, then d_S is Euclidean. The theoretical guarantee of d_S being Euclidean is as follows.

Theorem 1 *GLaD is Euclidean if and only if* $\rho \in [0, 1)$.

Proof Assume that the tree is connected and all retained edge weights are strictly positive, so every diagonal entry of $\mathbf{D}$ is positive. Define the normalized adjacency matrix

$$\mathbf{\Omega} = \mathbf{D}^{-1/2}\mathbf{A}\mathbf{D}^{-1/2}.$$

Although $\mathbf{D}^{-1}\mathbf{A}$ is generally not symmetric, it is similar to the real symmetric matrix $\mathbf{\Omega}$ and therefore has the same real eigenvalues. Moreover, $\mathbf{D}^{-1}\mathbf{A}$ is row-stochastic: its entries are nonnegative and each row sums to one. Gershgorin's theorem places all of its eigenvalues in the closed unit disk. Because the eigenvalues are real, they lie in $[-1, 1]$. The row-stochastic matrix also has eigenvalue one, with eigenvector $\mathbf{1}$, so the largest eigenvalue of $\mathbf{\Omega}$ is exactly one.

We may write

$$\mathbf{L}_P = \mathbf{D}^{1/2}(\mathbf{I} - \rho\mathbf{\Omega})\mathbf{D}^{1/2}.$$

For $0 \leq \rho < 1$, every eigenvalue of $\mathbf{I} - \rho\mathbf{\Omega}$ is at least $1 - \rho > 0$. Hence $\mathbf{L}_P$ and its inverse $\mathbf{B} = \mathbf{L}_P^{-1}$ are positive definite. The quadratic-form distance is therefore Euclidean, since it

equals the ordinary Euclidean distance between the transformed vectors $\mathbf{B}^{1/2}\mathbf{p}_r$ and $\mathbf{B}^{1/2}\mathbf{p}_s$. At $\rho = 1$, the eigenvalue one of $\boldsymbol{\Omega}$ makes $\mathbf{L}_P$ singular, so this ordinary-inverse construction is undefined; the separate spectral construction for that case is given in Section 1.3. □

This Euclidean guarantee ensures compatibility with a wide range of statistical and ordination methods, such as PCoA, PERMANOVA, and kernel-based regression or association tests.

1.3 GLaD with the Classical Graph Laplacian

The case of $\rho = 1$ corresponds to the classical graph Laplacian, in which the full phylogenetic structure is incorporated without attenuation. However, under this setting, the Laplacian matrix $\mathbf{L}_P = \mathbf{D} - \mathbf{A}$ becomes singular, and its inverse $\mathbf{L}_P^{-1}$ is undefined. This poses a computational challenge for deriving the evolutionary covariance matrix $\mathbf{B}$ and subsequently the GLaD dissimilarity. To address this issue, we develop an alternative formulation that bypasses the direct inversion of $\mathbf{L}_P$ while preserving the interpretability and topological sensitivity of GLaD. This variant enables stable distance computation even when $\rho = 1$, allowing practitioners to leverage the complete phylogenetic signal in settings where strong evolutionary correlation is presumed.

The alternative computation of GLaD is based on truncated eigendecomposition of the graph Laplacian. This approach avoids the ill-posed inversion of $\mathbf{L}_P$ while preserving the embedding-based interpretation of phylogenetic similarity. To stabilize the eigenspectrum, we use the regularized Laplacian $\mathbf{L}_P^{\text{reg}} = \mathbf{L}_P + \varepsilon\mathbf{I}$ and its truncated eigendecomposition $\mathbf{L}_P^{\text{reg}} \approx \mathbf{U}_k\boldsymbol{\Lambda}_k\mathbf{U}_k^\top$, retaining eigenvalues greater than ε. Here, $\mathbf{U}_k$ contains the retained eigenvectors and $\boldsymbol{\Lambda}_k$ is the diagonal matrix of their eigenvalues. The matrix construction and computational steps are detailed in Section 1.4. Let $\mathbf{P}$ be the relative abundance matrix of all samples (samples as rows). We project $\mathbf{P}$ into the Laplacian eigenbasis:

$$\mathbf{Z} = \mathbf{P}\mathbf{U}_k\boldsymbol{\Lambda}_k^{-1/2}.$$

The distance between samples r and s in this transformed space is given by the Euclidean distance between their projections:

$$d_{\text{GLaD}}(r, s) = \|\mathbf{z}_r - \mathbf{z}_s\|_2\,,$$

where $\mathbf{z}_r$ and $\mathbf{z}_s$ are the corresponding rows of $\mathbf{Z}$. This formulation yields a valid Euclidean dissimilarity matrix and remains fully compatible with kernel-based inference, ordination, and statistical testing. Weighted and unweighted versions of GLaD are obtained by projecting relative-abundance or presence–absence data, respectively.

1.4 Calculation of the Evolutionary Covariance Matrix

A key step in computing GLaD is the construction of the evolutionary covariance matrix. In this subsection, we describe how this matrix is obtained from a phylogenetic tree. In practice, the evolutionary covariance matrix was derived directly from the phylogenetic tree through three steps. First, the tree was converted into a branch-length-weighted adjacency matrix $\mathbf{A}$, in which nonzero entries correspond to directly

connected nodes and are set equal to the respective branch lengths. Second, the diagonal degree matrix $\mathbf{D}$ was constructed, with each diagonal entry equal to the total branch length incident to the corresponding node. Third, the graph Laplacian

$$\mathbf{L}_P = \mathbf{D} - \rho\mathbf{A}$$

was formed, and the evolutionary covariance matrix was defined as $\mathbf{B} = \mathbf{L}_P^{-1}$ for $0 \le \rho < 1$. Larger entries of $\mathbf{B}$ therefore reflect stronger evolutionary coupling between nodes, after accounting for both path length and local branching structure. The construction is detailed below.

Given a `phyloseq` object with a phylogenetic tree stored in `phyloseq@tree`, the matrix `phyloseq@tree$edge` provides the edge list of the tree, with each row corresponding to a parent–child branch, and `phyloseq@tree$edge.length` provides the associated branch lengths. We construct the evolutionary proximity matrix $\mathbf{A}$ over all tip and internal nodes by assigning each branch length to the corresponding edge. Specifically, if nodes u and v are connected by a branch with length ℓ_{uv}, we set

$$A_{uv} = A_{vu} = \ell_{uv},$$

and set $A_{uv} = 0$ otherwise. Thus, the sparsity pattern of $\mathbf{A}$ encodes the tree topology, whereas its nonzero entries preserve the branch-length information. The degree matrix $\mathbf{D}$ is defined as the diagonal matrix whose diagonal entries are the row sums of $\mathbf{A}$.

When the user-specified parameter satisfies $\rho < 1$, the matrix $\mathbf{L}_P = \mathbf{D} - \rho\mathbf{A}$ is nonsingular, and we compute

$$\mathbf{B} = \mathbf{L}_P^{-1}$$

directly using the `solve()` function. When $\rho = 1$, the graph Laplacian may be singular. In this case, we follow the regularization strategy introduced by (9). We apply a small diagonal shift $\varepsilon\mathbf{I}$, yielding the regularized Laplacian

$$\mathbf{L}_P^{\text{reg}} = \mathbf{L}_P + \varepsilon\mathbf{I}.$$

We then compute its truncated eigendecomposition,

$$\mathbf{L}_P^{\text{reg}} \approx \mathbf{U}_k\mathbf{\Lambda}_k\mathbf{U}_k^\top,$$

where $\mathbf{U}_k \in \mathbb{R}^{n\times k}$ contains the eigenvectors corresponding to the retained eigenvalues, and

$$\mathbf{\Lambda}_k = \text{diag}(\lambda_1, \dots, \lambda_k)$$

contains eigenvalues satisfying $\lambda_i > \varepsilon$. The evolutionary covariance matrix is then approximated by

$$\mathbf{B} = \mathbf{U}_k\mathbf{\Lambda}_k^{-1}\mathbf{U}_k^\top.$$

1.5 Interpretation of Koinetic

The interpretation of koinetic is clearest by contrasting its construction with branch-wise phylogenetic distances such as UniFrac. UniFrac accumulates branch-length-weighted differences through branch-specific contributions, whereas koinetic first encodes the full weighted phylogenetic graph in the evolutionary covariance matrix $\mathbf{B}$ and then evaluates the complete community-difference vector jointly through a single quadratic form. This quadratic form makes explicit the two ideas represented by the name *koinos kinetic*. The *koinos* component refers to the collective evaluation of community differences across phylogenetically connected nodes, whereas the *kinetic* component refers to the diffusion of these differences through the phylogenetic graph. To see the first component, let

$$\boldsymbol{\delta}_{rs} = \mathbf{p}_r - \mathbf{p}_s = (\delta_{rs,1}, \ldots, \delta_{rs,N})^\top.$$

Then the squared koinetic distance can be written elementwise as

$$\begin{aligned} d_S^2(r,s) &= \boldsymbol{\delta}_{rs}^\top \mathbf{B} \boldsymbol{\delta}_{rs} \\ &= \sum_{i=1}^{N} B_{ii}\delta_{rs,i}^2 + 2 \sum_{1 \le i < j \le N} B_{ij}\delta_{rs,i}\delta_{rs,j}. \end{aligned} \tag{11}$$

The first term, $B_{ii}\delta_{rs,i}^2$, is the node-specific contribution of the difference at node i, weighted by its self-influence under the phylogenetic diffusion kernel. Thus, a difference occurring at a node with stronger self-influence in $\mathbf{B}$ contributes more strongly to the overall distance. The second term, $2B_{ij}\delta_{rs,i}\delta_{rs,j}$, is a cross-lineage diffusion contribution. The entry B_{ij} determines how strongly differences at nodes i and j are coupled through the phylogenetic graph. When the two node-level differences have the same sign, $\delta_{rs,i}\delta_{rs,j} > 0$, their contributions reinforce one another through phylogenetic diffusion; when they have opposite signs, $\delta_{rs,i}\delta_{rs,j} < 0$, they partially offset one another. Consequently, the contribution of a node is evaluated jointly with differences occurring at phylogenetically connected nodes rather than being accumulated only as an independent branch-wise contribution. In particular, concordant differences at related nodes can reinforce one another through the off-diagonal terms of $\mathbf{B}$. This collective accumulation of both node-specific and cross-lineage diffusion contributions is the *koinos* aspect of koinetic.

The *kinetic* component is encoded by the evolutionary covariance matrix $\mathbf{B} = (\mathbf{D} - \rho\mathbf{A})^{-1}$. As shown in Equations (1)–(3) of the main text, for $0 \le \rho < 1$ it admits the diffusion expansion

$$\mathbf{B} = \mathbf{D}^{-1/2}\left(\sum_{k=0}^{\infty} \rho^k \boldsymbol{\Omega}^k\right)\mathbf{D}^{-1/2}, \qquad \boldsymbol{\Omega} = \mathbf{D}^{-1/2}\mathbf{A}\mathbf{D}^{-1/2}. \tag{12}$$

Each power $\boldsymbol{\Omega}^k$ collects propagation through walks of length k on the phylogenetic graph, while ρ^k attenuates longer-range propagation and the degree normalization

accounts for the local branching structure encountered along the walk. At the entry level, this gives

$$B_{ij} = \sum_{k=0}^{\infty} \rho^k \sum_{\text{walk } i \to j} \frac{\prod_{(u,v)\in \text{walk}} \ell_{uv}}{\prod_{u \in \text{walk nodes}} d_u}. \tag{13}$$

Here, ℓ_{uv} denotes the branch length of edge (u, v) and $d_u = \sum_v \ell_{uv}$ is the degree of node u. Because a phylogenetic tree contains a unique simple path between any two nodes, the dominant contribution is associated with that path. Denoting it by $\mathcal{P}(i, j)$, with $k(i, j)$ edges, yields the approximation

$$B_{ij} \approx \rho^{k(i,j)} \frac{\prod_{(u,v)\in \mathcal{P}(i,j)} \ell_{uv}}{\prod_{u \in \mathcal{P}(i,j)} d_u}. \tag{14}$$

Thus, B_{ij} describes how strongly a difference at one node can diffuse to another through their shared evolutionary structure. As discussed in the main text, this diffusion is stronger when the two nodes are hierarchically close, because fewer propagation steps are required, and when the branches on their connecting path constitute relatively large components of the incident degrees of the intermediate nodes. The *kinetic* aspect of koinetic therefore converts a local node-level difference into a phylogenetically diffused signal that can extend through nearby lineages, shared ancestors, and longer evolutionary paths.

Taken together, GLaD evaluates microbial-community differences through joint phylogenetic diffusion rather than only through a sum of branch-wise changes. The weighted version injects relative-abundance differences into this diffusion geometry and can therefore be interpreted as the phylogenetic diffusion of abundance differences. The unweighted version injects presence–absence differences into the same geometry and can be interpreted as the phylogenetic diffusion of occurrence differences. When occurrence differences are concentrated among related taxa, the off-diagonal terms allow these differences to contribute collectively through the fixed phylogenetic structure. The two versions therefore share the same koinos–kinetic mechanism and differ only in the type of community signal that is propagated through the phylogenetic tree.

1.6 Benchmarking Framework and Evaluation Indices

Let $\mathbf{D} \in \mathbb{R}^{n \times n}$ denote a dissimilarity matrix computed between n biological samples, where each entry D_{ij} reflects the dissimilarity between samples i and j. To assess the practical utility of such dissimilarities in microbiome studies, we develop a rigorous and extensible benchmarking framework to evaluate their performance across multiple real-world datasets.

This framework comprises two principal categories of evaluation metrics: geometry-based indices and clustering-based indices, each capturing a complementary aspect of dissimilarity utility in downstream analysis. Geometry-based indices assess how well the dissimilarity matrix preserves the intrinsic geometry of the data when embedded into a low-dimensional space. Specifically, we consider the Fraction of Negative Inertia (FNI), trustworthiness, continuity, Mantel correlation, and stress. Clustering-based

indices evaluate the extent to which a dissimilarity supports biologically meaningful sample partitioning, using a standard unsupervised metric, the silhouette score.

To integrate these heterogeneous metrics into a unified selection tool, we further propose a simple, rank-based Dissimilarity Recommendation Score (DRS). This composite score summarizes the relative standing of each dissimilarity measure across all evaluation metrics, offering a scalable and interpretable criterion for selecting optimal dissimilarities tailored to specific datasets.

1.6.1 Geometry-based Indices

We begin with the Fraction of Negative Inertia (FNI) (3), a global diagnostic that quantifies the degree to which a dissimilarity matrix violates Euclidean assumptions. The Gram matrix is computed as

$$G = -\frac{1}{2} J D^{(2)} J, \quad \text{where } J = I_N - \frac{1}{N}\mathbf{1}\mathbf{1}^\top.$$

If the dissimilarity matrix D is Euclidean, then $D^{(2)}$ is conditionally negative definite (CND), and the resulting Gram matrix G is guaranteed to be positive semi-definite (PSD). However, when D is non-Euclidean, G may possess negative eigenvalues, indicating curvature or non-flatness in the original dissimilarity geometry.

To quantify the extent of this non-Euclidean structure, we define FNI as:

$$\text{FNI} = \frac{\sum_{i=1}^{N} |\lambda_i| \cdot \mathbb{I}(\lambda_i < 0)}{\sum_{i=1}^{N} |\lambda_i|},$$

where λ_i denotes the i-th eigenvalue of G. FNI measures the proportion of total inertia (i.e., total variance-like quantity) that cannot be attributed to Euclidean components. A higher FNI indicates that a larger fraction of the dissimilarity-induced variation cannot be faithfully embedded in a flat Euclidean space, thereby compromising not only the interpretability of PCoA, but also the validity of downstream analyses that implicitly assume Euclidean geometry, such as PERMANOVA, kernel-based association testing, and distance-based ordination. In such cases, the use of non-Euclidean dissimilarities may lead to distorted effect size estimation, biased clustering, and spurious detection of group structure due to curvature-induced artifacts.

To assess whether a low-dimensional embedding faithfully preserves the local structure of the original dissimilarity space, we compute two classical metrics from nonlinear dimensionality reduction: trustworthiness and continuity (10, 11).

Given the original dissimilarity matrix D and the Euclidean distance matrix D_{low} computed from a low-dimensional embedding (e.g., PCoA), we compare the top-k nearest neighbors of each sample under both matrices. Trustworthiness penalizes neighbors that appear in D_{low} but not in D, capturing false positives introduced by the embedding. Continuity penalizes true neighbors in D that are missing in D_{low}, capturing false negatives. Let $k = \min(50, \max(5, \lceil n/10 \rceil))$ denote the neighborhood size. For each sample i, define U_i as the set of neighbors present in D_{low} but not in D, and V_i

as the reverse. The metrics are computed as:

$$T = 1 - \frac{2}{nk(2n - 3k - 1)} \sum_{i=1}^{n} \sum_{j \in U_i} (\mathrm{rank}_D(j) - k),$$

$$C = 1 - \frac{2}{nk(2n - 3k - 1)} \sum_{i=1}^{n} \sum_{j \in V_i} (\mathrm{rank}_{D_{\mathrm{low}}}(j) - k).$$

Both T and C lie in $[0, 1]$, with higher values indicating better neighborhood preservation. Together, they quantify the geometric fidelity of ordination plots and reflect a dissimilarity's ability to balance structure preservation with outcome separation.

To evaluate the global structural agreement between the original dissimilarity matrix and its low-dimensional embedding, we compute the Mantel correlation (12) between D and D_{low}. The Mantel statistic is defined as the Pearson correlation coefficient between the vectorized upper triangular parts of the two matrices:

$$r_M = \mathrm{corr}\left(\mathrm{vec}(\mathrm{upper}(D)),\ \mathrm{vec}(\mathrm{upper}(D_{\mathrm{low}}))\right),$$

where $\mathrm{upper}(\cdot)$ extracts the upper triangular elements (excluding the diagonal) and $\mathrm{vec}(\cdot)$ flattens them into vectors.

Unlike trustworthiness and continuity, which focus on local neighborhoods, Mantel correlation captures the global geometric fidelity of the embedding. A higher Mantel correlation indicates that the relative pairwise dissimilarities are well preserved in the low-dimensional space, even if the embedding distorts neighborhood ranks. We include Mantel correlation in our evaluation system to provide a complementary, topology-aware metric that emphasizes the overall preservation of dissimilarity structure. This global perspective is particularly important in microbiome studies where long-range distances may reflect underlying gradients, batch effects, or ecological stratification that are invisible to local measures.

We additionally report the classical stress score from multidimensional scaling (MDS) as a global measure of geometric distortion. Given a dissimilarity matrix D and a low-dimensional embedding that induces Euclidean distances D_{low}, the stress is defined as:

$$\mathrm{Stress} = \sqrt{\frac{\sum_{i<j} (D_{ij} - D_{\mathrm{low},ij})^2}{\sum_{i<j} D_{ij}^2}}.$$

A lower stress value indicates that the embedding better approximates the original dissimilarities in a Euclidean space. While Mantel correlation focuses on rank consistency, stress emphasizes the preservation of absolute pairwise distances. We include it as a standard diagnostic of global embedding fidelity, widely used in ecological ordination and dissimilarity-based analysis.

The choice of these indices is motivated by the central role that dissimilarity measures play as beta diversity metrics in microbiome research. In practice, a dissimilarity matrix is rarely analyzed directly in its raw matrix form. Instead, it is typically subjected to ordination techniques to enable visualization and downstream interpretation. Among these approaches, methods that embed samples into a low-dimensional

Euclidean space—such as Principal Coordinates Analysis (PCoA)—are by far the most commonly used. The goal of ordination, however, is not merely dimensionality reduction in the generic sense of projection, but rather the preservation of pairwise dissimilarity relationships: samples that are close in the original dissimilarity space should remain close in the embedded representation, and large separations should likewise be maintained.

A particularly desirable scenario arises when the dissimilarity satisfies the Euclidean property. In that case, the matrix admits an exact embedding into a Euclidean space, and classical metric embedding theory guarantees that PCoA preserves all pairwise distances without distortion (up to numerical precision). Consequently, Euclidean dissimilarities naturally align with the geometry of ordination and provide theoretical assurance that downstream visualization and geometric interpretation are meaningful. In contemporary microbiome studies, this Euclidean property is therefore regarded as an important practical advantage, as it directly affects the stability, interpretability, and fidelity of ordination-based analyses.

For these reasons, the geometry-based indices included in our benchmarking framework are specifically chosen to quantify either (i) the extent to which a dissimilarity satisfies or approximates the Euclidean property (e.g., Fraction of Negative Inertia), or (ii) the degree to which pairwise relationships are preserved after low-dimensional embedding (e.g., stress, Mantel correlation, trustworthiness, and continuity). Together, these metrics provide a principled evaluation of whether a dissimilarity measure supports faithful geometric representation, which is a key requirement for its practical utility in microbiome beta diversity analysis.

1.6.2 Clustering-based Indices

To evaluate how well each dissimilarity measure supports biologically meaningful sample stratification, we compute the silhouette score, a widely used clustering validity index. It requires a fixed partition of samples into k non-overlapping clusters. To ensure consistency across dissimilarity measures, we adopt k-medoids (PAM) (13) as the unified clustering algorithm. Unlike k-means, which assumes a Euclidean geometry and uses abstract centroids, k-medoids operates directly on the dissimilarity matrix and selects actual samples as cluster centers (medoids), yielding interpretable and robust clusters. It is particularly well suited for non-Euclidean dissimilarities and has been widely used in ecological and compositional data analysis. The number of clusters k can be chosen in two ways depending on the context: (i) when ground truth group labels are available (e.g., treatment or disease status), we set k to match the known number of biological groups; (ii) when no prior labels exist, we determine k using internal criteria such as the gap statistic (14). In both cases, the same k is used across all dissimilarity measures to ensure comparability of clustering-based indices.

The silhouette score (15) quantifies the cohesion and separation of clusters based on pairwise dissimilarities. For each sample i, let $a(i)$ denote the average dissimilarity between i and all other samples within its assigned cluster, and let $b(i)$ denote the minimum average dissimilarity between i and any other cluster. The silhouette value

for i is then defined as:

$$s(i) = \frac{b(i) - a(i)}{\max\{a(i), b(i)\}}.$$

The silhouette score ranges from -1 to 1, with higher values indicating that samples are well matched to their own cluster and poorly matched to neighboring clusters. The overall silhouette score is computed as the average of $s(i)$ across all samples. We include the silhouette score as a primary clustering index because it captures both intra-cluster compactness and inter-cluster separability, and it is compatible with arbitrary dissimilarity matrices. In the context of microbiome analysis, a higher silhouette score reflects clearer ecological boundaries between sample groups under the given dissimilarity measure.

The Calinski–Harabasz Index (CHI) (16) measures clustering quality by comparing the between-cluster dispersion to the within-cluster dispersion, under the assumption that well-separated clusters should exhibit high inter-cluster variation and low intra-cluster variance. Although CHI is traditionally designed for centroid-based methods such as k-means, we compute it in the low-dimensional Euclidean space derived from principal coordinates (typically the first 2 or 3 PCoA axes), where cluster centroids and variances are well-defined. Let $\{C_1, \ldots, C_k\}$ denote the k clusters in this Euclidean embedding, and let $\bar{\mathbf{x}}$ be the global centroid. For each cluster C_i, let $\bar{\mathbf{x}}_i$ denote its centroid and n_i its number of points. The between-cluster and within-cluster dispersions are defined as:

$$S_B = \sum_{i=1}^{k} n_i \|\bar{\mathbf{x}}_i - \bar{\mathbf{x}}\|^2, \quad S_W = \sum_{i=1}^{k} \sum_{\mathbf{x} \in C_i} \|\mathbf{x} - \bar{\mathbf{x}}_i\|^2.$$

The CHI is then calculated as:

$$\text{CHI} = \frac{S_B/(k-1)}{S_W/(n-k)},$$

where n is the total number of samples. Higher CHI values indicate more distinct and compact clusters. Compared with silhouette and the Dunn index, CHI provides a global variance-based criterion, focusing on overall cluster separability in the embedding space rather than sample-level cohesion or worst-case overlap.

The Dunn Index (17) evaluates clustering quality by identifying the minimum inter-cluster separation relative to the maximum intra-cluster dispersion. Formally, for a given clustering $\{C_1, \ldots, C_k\}$, the Dunn Index is defined as:

$$\text{Dunn} = \frac{\min_{1 \le i < j \le k} \delta(C_i, C_j)}{\max_{1 \le \ell \le k} \Delta(C_\ell)},$$

where $\delta(C_i, C_j)$ denotes the dissimilarity between clusters C_i and C_j, and $\Delta(C_\ell)$ denotes the intra-cluster diameter of cluster C_ℓ. In our implementation, both terms are computed using the dissimilarity matrix: $\delta(C_i, C_j)$ is the minimal pairwise dissimilarity between any sample in C_i and any sample in C_j, and $\Delta(C_\ell)$ is the maximal pairwise

dissimilarity within C_ℓ. A higher Dunn Index indicates that clusters are well-separated and internally compact. Compared to CHI and DBI, which aggregate overall variance or average separation, the Dunn index adopts a conservative perspective by emphasizing the worst-case clustering configuration. This makes it particularly valuable for detecting cases where a small number of overlapping or diffuse clusters undermine the overall clustering structure.

1.6.3 Rank Aggregation for the Tournament Plots

The tournament plots summarize six criteria defined above: fraction of negative inertia, trustworthiness, continuity, Mantel correlation, stress, and average silhouette. Lower fraction of negative inertia and stress, and higher values of the other four criteria, indicate better performance. Each column displays the corresponding method ranks, with rank 1 denoting the best result.

For the aggregate Borda score, let $r_{i\ell}$ be the rank of method i among m candidate methods for criterion ℓ. The score is

$$B_i = \sum_{\ell=1}^{L} (m - r_{i\ell}),$$

where $L = 6$ for these plots and larger scores indicate better overall rankings (18, 19). The final column displays the ranking by this aggregate score. Criterion-specific ranks remain visible so that the reader can distinguish overall performance from strengths or weaknesses on individual criteria. The corresponding plots are presented in Section 3.4.

2 Simulation Study

2.1 Simulation Model and Perturbation Mechanisms

SparseDOSSA2 (20) provides a generative framework for simulating realistic microbial community count tables from a fitted template dataset. Its key idea is to separate the latent biological abundance-generating process from the observed sequencing process. For each sample i and microbial feature j, SparseDOSSA2 introduces an unobserved absolute abundance A_{ij}, which follows a zero-inflated log-normal marginal distribution. Specifically, feature j is absent with probability π_j, and conditional on being present, its log absolute abundance follows a normal distribution with mean μ_j and variance σ_j^2:

$$A_{ij} = \begin{cases} 0, & \text{with probability } \pi_j, \\ \exp(Z_{ij}), \quad Z_{ij} \sim N(\mu_j, \sigma_j^2), & \text{with probability } 1 - \pi_j. \end{cases}$$

Feature-feature dependence is modeled through a Gaussian copula, allowing the simulated microbial community to preserve correlation patterns estimated from the template data. The latent absolute abundance vector is then normalized to relative

abundance,

$$X_{ij} = \frac{A_{ij}}{\sum_{k=1}^{p} A_{ik}},$$

which enforces the compositional constraint. Finally, given a sample-specific sequencing depth D_i, the observed count vector is generated by multinomial sampling,

$$(C_{i1}, \ldots, C_{ip}) \sim \text{Multinomial}\left(D_i; X_{i1}, \ldots, X_{ip}\right).$$

Thus, the final simulated OTU table is produced through the sequence

$$A_{ij} \longrightarrow X_{ij} = A_{ij} / \sum_k A_{ik} \longrightarrow C_{ij}.$$

In our simulation study, SparseDOSSA2 is first fitted to a real template OTU table to estimate the baseline feature-specific parameters $(\pi_j, \mu_j, \sigma_j^2)$, the dependence structure among features, and the sequencing-depth distribution. This fitted model defines a realistic null microbial community. We then introduce controlled signals into a prespecified set of spike-in features. These spike-in features are chosen according to the simulation design, for example from a phylogenetically localized cluster. For each spike-in feature, SparseDOSSA2 perturbs either its prevalence component, its nonzero abundance component, or both, depending on the selected signal mode.

For an abundance signal, SparseDOSSA2 modifies the mean of the nonzero log absolute abundance distribution. Let G_i denote the group indicator or phenotype variable used to define the perturbation, and let β_{abund} be the user-specified abundance effect size. For a spike-in feature j, the mean log abundance in sample i becomes

$$\mu_{ij} = \mu_j + G_i \beta_{\text{abund}}.$$

Equivalently, conditional on being present,

$$\log A_{ij} \mid A_{ij} > 0, G_i \sim N(\mu_j + G_i \beta_{\text{abund}}, \sigma_j^2).$$

Because the perturbation is applied on the log scale, β_{abund} has the interpretation of a log fold change in the latent absolute abundance. For example, $\beta_{\text{abund}} = \log(2)$ corresponds to a two-fold increase in nonzero absolute abundance in the perturbed group.

For a prevalence signal, SparseDOSSA2 modifies the probability that a feature is present. Since π_j denotes the absence probability, the baseline presence probability is $1 - \pi_j$. Let β_{prev} denote the user-specified prevalence effect size. SparseDOSSA2 perturbs the log-odds of presence according to

$$\log\left(\frac{1 - \pi_{ij}}{\pi_{ij}}\right) = \log\left(\frac{1 - \pi_j}{\pi_j}\right) + G_i \beta_{\text{prev}}.$$

Thus, β_{prev} is interpreted as a log odds ratio for feature presence. Positive values increase the probability that the feature is present in the perturbed group, whereas negative values decrease it.

When the signal mode is set to abundance only, SparseDOSSA2 modifies μ_j for the selected spike-in features while keeping the baseline absence probability π_j unchanged. When the signal mode is set to prevalence only, SparseDOSSA2 modifies π_j through the logit-presence model while keeping the nonzero abundance distribution unchanged. When the signal mode is set to both, both components are perturbed simultaneously:

$$\mu_{ij} = \mu_j + G_i\beta_{\text{abund}},$$

and

$$\log\left(\frac{1-\pi_{ij}}{\pi_{ij}}\right) = \log\left(\frac{1-\pi_j}{\pi_j}\right) + G_i\beta_{\text{prev}}.$$

SparseDOSSA2 permits separate effect sizes to be specified for the abundance and prevalence components. In our simulations, however, we used a single nominal effect size and mapped it to the two components according to the selected signal mode, as described below.

In our simulation study, a single nominal effect size $\beta \in \{0, 0.6, 0.8, 1.2, 1.6, 2.0\}$ was specified for each simulation setting. Its mapping to the two component-specific perturbation parameters depended on the signal mode. Specifically,

$$(\beta_{\text{abund}}, \beta_{\text{prev}}) = \begin{cases} (\beta, 0), & \text{under the abundance-only mode,} \\ (0, \beta), & \text{under the prevalence-only mode,} \\ (\beta, \beta), & \text{under the combined abundance-and-prevalence mode.} \end{cases}$$

Accordingly, under the abundance-only mode, the selected features differed between groups only in their nonzero abundance distributions; under the prevalence-only mode, they differed only in their probabilities of occurrence; and under the combined mode, the same nominal effect size was applied simultaneously to both components. When $\beta = 0$, both component-specific perturbation coefficients were zero under all three modes, so that no group-dependent signal was introduced.

A useful feature of the SparseDOSSA2 spike-in mechanism is that perturbations are introduced on the latent absolute-abundance scale before compositional normalization. This is important for microbiome benchmarking because it provides a clear definition of true signal features. The selected spike-in features are the taxa whose latent biological prevalence and/or abundance parameters are directly modified. Other taxa are not directly perturbed, although their observed relative abundances may change indirectly after normalization because all features must sum to one within each sample. Therefore, SparseDOSSA2 distinguishes between true biological perturbations and secondary compositional changes induced by normalization.

After the prevalence and/or abundance parameters have been modified for the spike-in features, SparseDOSSA2 generates a new absolute abundance matrix from the perturbed zero-inflated log-normal model. The simulated absolute abundances are then normalized to relative abundances, and sequencing counts are generated by multinomial sampling using simulated or user-specified sequencing depths. The resulting OTU table therefore retains realistic sparsity, marginal abundance distributions, feature dependence, compositionality, and sequencing-depth variation inherited from

the template dataset, while containing controlled ground-truth signals in the selected spike-in features.

2.2 Signal Clusters and Power Summaries

To evaluate how different dissimilarity measures respond to phylogenetically localized signals, we constructed signal clusters using the reference phylogenetic tree from the throat microbiome dataset. Specifically, we first computed the patristic distance matrix among OTUs, where each entry represents the total branch length separating two OTUs on the phylogenetic tree. We then applied partitioning around medoids (PAM) to this distance matrix, with the number of clusters set to $K \in \{2, 4, 6, 8, 10, 12\}$. This procedure groups OTUs according to their evolutionary dissimilarity, so that OTUs assigned to the same cluster are more phylogenetically similar to each other than to OTUs in other clusters. The parameter K controls the granularity of the phylogenetic signal: smaller values of K produce larger and coarser signal clusters, whereas larger values of K produce smaller and more localized clusters. For each fixed K, we iteratively selected the kth cluster, $k = 1, \ldots, K$, as the signal cluster and treated all OTUs in that cluster as spike features in the simulation. This design avoids placing signals in a single preselected clade and instead allows us to systematically assess how detection performance depends on both the phylogenetic location and the granularity of the perturbation.

Because PAM clustering based on patristic distances can produce clusters with substantial heterogeneity in size, abundance, and phylogenetic configuration, we summarized power in three complementary ways. First, for each K, we reported the empirical power obtained when each individual cluster $k = 1, \ldots, K$ was used as the signal cluster. This cluster-specific summary is the most direct and objective representation of the simulation results, because it preserves the heterogeneity induced by the reference tree and does not average over biologically or phylogenetically distinct signal locations. It also allows us to examine whether a method is uniformly sensitive across the tree or whether its performance depends strongly on where the signal is introduced.

Second, for each K, we computed an abundance-weighted average of the cluster-specific powers. Let $\widehat{\text{Power}}_{Kk}$ denote the empirical power when the kth cluster under granularity K is used as the signal cluster, and let w_{Kk} denote the relative abundance weight of that cluster, defined by its total abundance in the reference dataset normalized across all K clusters. The abundance-weighted power is then

$$\widehat{\text{Power}}_K^{\text{wavg}} = \sum_{k=1}^{K} w_{Kk} \widehat{\text{Power}}_{Kk}, \qquad \sum_{k=1}^{K} w_{Kk} = 1.$$

This summary gives greater influence to clusters that represent more abundant portions of the empirical community and therefore may better reflect the expected performance of a method under signals arising from dominant or commonly observed lineages. However, this quantity should be interpreted as a weighted descriptive summary rather than a replacement for the cluster-specific results. In particular,

abundance weighting may obscure poor performance on rare but phylogenetically meaningful clusters, and it implicitly prioritizes signals in high-abundance regions of the tree.

Third, for each K, we reported the empirical power when the largest cluster, defined as the cluster containing the greatest number of OTUs, was used as the signal cluster. This summary is motivated by the fact that not every PAM-derived cluster necessarily corresponds to a biologically interpretable or stable phylogenetic region, especially when K is large and some clusters may contain very few OTUs. The largest cluster typically provides a more robust and well-populated signal set, making it a useful representative scenario for assessing power under a clear and sufficiently broad phylogenetic perturbation. This analysis provides a stable and interpretable benchmark for evaluating method performance under a well-defined phylogenetic signal.

Together, these three summaries provide a more complete picture of method performance than any single power estimate. The cluster-specific powers characterize location-dependent sensitivity, the abundance-weighted average provides an empirically weighted overall summary, and the largest-cluster analysis offers a stable representative scenario based on a well-populated phylogenetic signal region.

2.3 Additional Results of Simulation Study

k	signal_mode	Unweighted GLaD (rho = 0.5)	Weighted GLaD (rho = 0.5)	Unweighted GLaD (rho = 1)	Weighted GLaD (rho = 1)	Unweighted UniFrac	Weighted UniFrac	GUniFrac	Aitchison
2	abundance	0.06	0.07	0.03	0.08	0.03	0.08	0.06	0.09
2	both	0.02	0	0.06	0.01	0.03	0.01	0.02	0.04
2	prevalence	0.06	0.05	0.06	0.05	0.06	0.03	0.03	0.03
4	abundance	0.05	0.03	0.05	0.01	0.06	0.03	0.03	0.02
4	both	0.07	0.05	0.04	0.06	0.06	0.05	0.07	0.1
4	prevalence	0.06	0.07	0.02	0.07	0.02	0.05	0.07	0.1
6	abundance	0.07	0.1	0.02	0.09	0.07	0.11	0.11	0.05
6	both	0.04	0.09	0.06	0.09	0.05	0.08	0.08	0.03
6	prevalence	0.06	0.06	0.06	0.05	0.09	0.07	0.06	0.06

Table S1: Empirical type I error based on the largest null cluster under each simulation setting. For each value of k and signal mode under the null scenario, the cluster containing the largest number of OTUs was selected, and type I error was computed as the false rejection rate within that selected cluster. The table summarizes method-specific type I error across simulation settings, with values closer to the nominal significance level indicating better error control.

Figures S2–S5 summarize the empirical power patterns across cluster configurations with $K = 2, 4, 6$, and 8, respectively. For each value of K, the corresponding figure contains three heatmaps representing the abundance-only, prevalence-only, and combined abundance–prevalence signal settings. These heatmaps show how detection power changes with both the number of clusters and the type of signal introduced. The corresponding cluster-level summaries are reported in Tables S3–S6. For each K, these tables report the signal cluster index, number of OTUs, overall abundance, and mean within-cluster cophenetic distance (COP). Together, the figures and tables connect the observed power patterns with the underlying cluster structure, providing context for interpreting method performance under different signal mechanisms and cluster configurations.

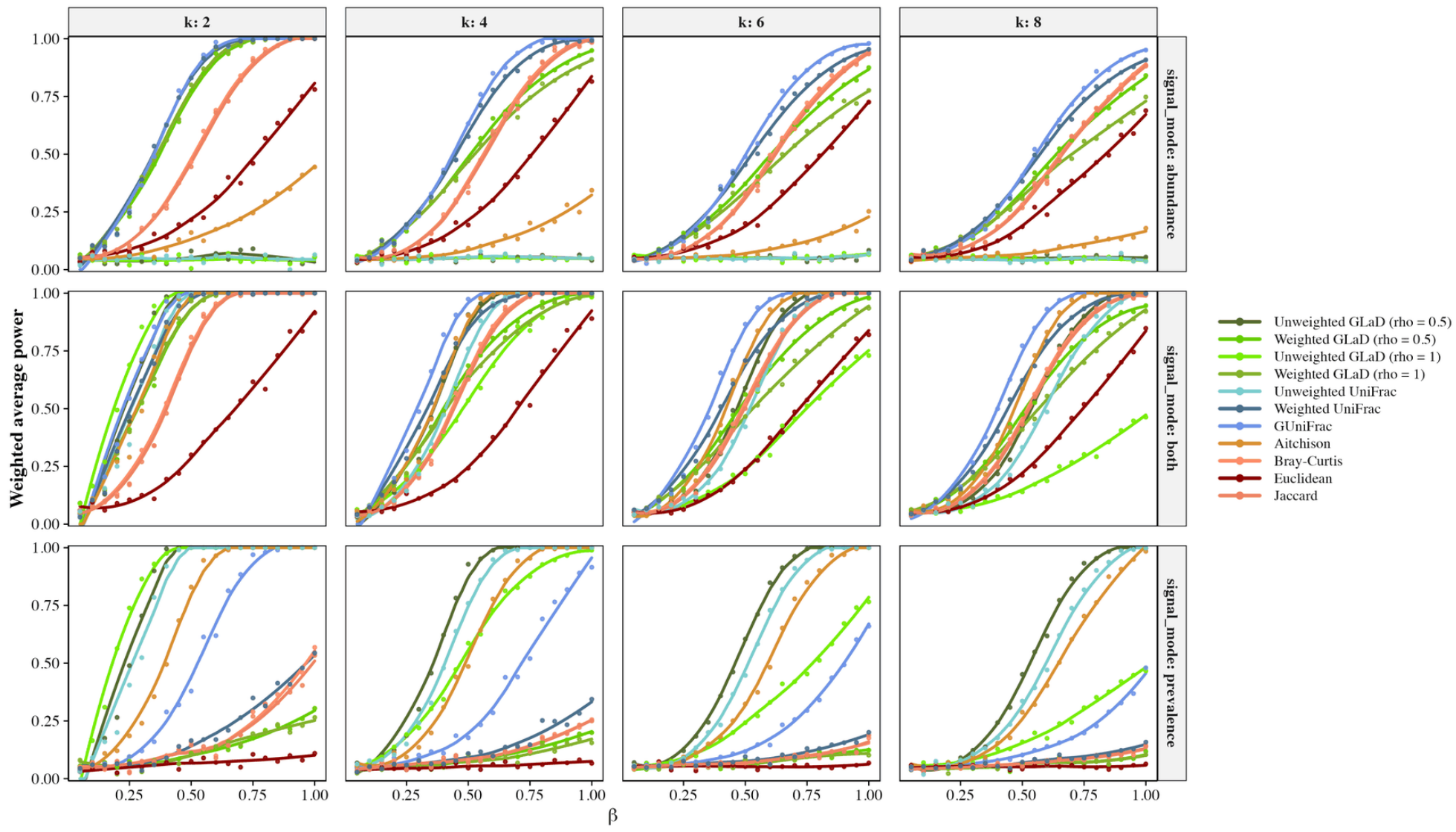

Fig. S1: Weighted average empirical power across signal clusters, where each cluster was weighted according to its overall abundance contribution. For each value of k, signal mode, and effect size β, power was summarized as the weighted average rejection rate across signal clusters. Points represent empirical weighted power values, while solid curves show smoothed trends across β. The smoothed curves were constrained to remain within $[0, 1]$, and once a curve reached power equal to 1, subsequent values were fixed at 1. Panels correspond to different combinations of k and signal mode.

k	signal_mode	Unweighted GLaD (rho = 0.5)	Weighted GLaD (rho = 0.5)	Unweighted GLaD (rho = 1)	Weighted GLaD (rho = 1)	Unweighted UniFrac	Weighted UniFrac	GUniFrac	Aitchison
2	abundance	0.06	0.0602	0.03	0.08	0.0398	0.0604	0.0502	0.0605
2	both	0.0396	0.0344	0.06	0.0296	0.0496	0.0444	0.0544	0.0449
2	prevalence	0.0502	0.0353	0.0453	0.0304	0.0698	0.0251	0.0398	0.0398
4	abundance	0.0549	0.034	0.0566	0.0254	0.0593	0.0348	0.0413	0.0341
4	both	0.0435	0.0683	0.0382	0.0765	0.0486	0.0592	0.0621	0.0686
4	prevalence	0.0538	0.0676	0.0345	0.0614	0.0287	0.0619	0.0556	0.0664
6	abundance	0.0461	0.0709	0.0342	0.0679	0.0523	0.0814	0.0698	0.0584
6	both	0.0421	0.0743	0.0564	0.0668	0.0433	0.0605	0.0596	0.0332
6	prevalence	0.0653	0.0558	0.0482	0.0465	0.077	0.0607	0.0579	0.0613

Table S2: Weighted average empirical type I error across null clusters, where each cluster was weighted according to both its overall abundance contribution and its mean relative abundance. For each value of k and signal mode under the null scenario, type I error was summarized as the weighted average false rejection rate across clusters. The table summarizes method-specific type I error across simulation settings, with values closer to the nominal significance level indicating better error control.

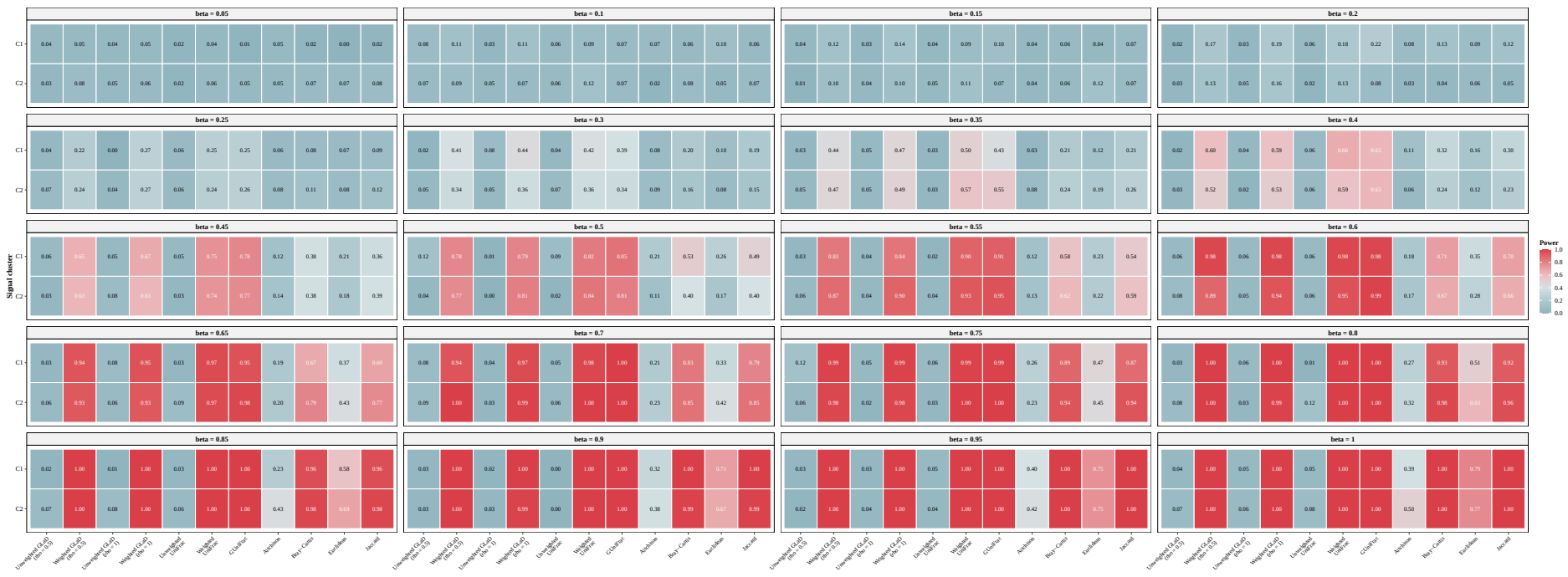

(a) Abundance signal

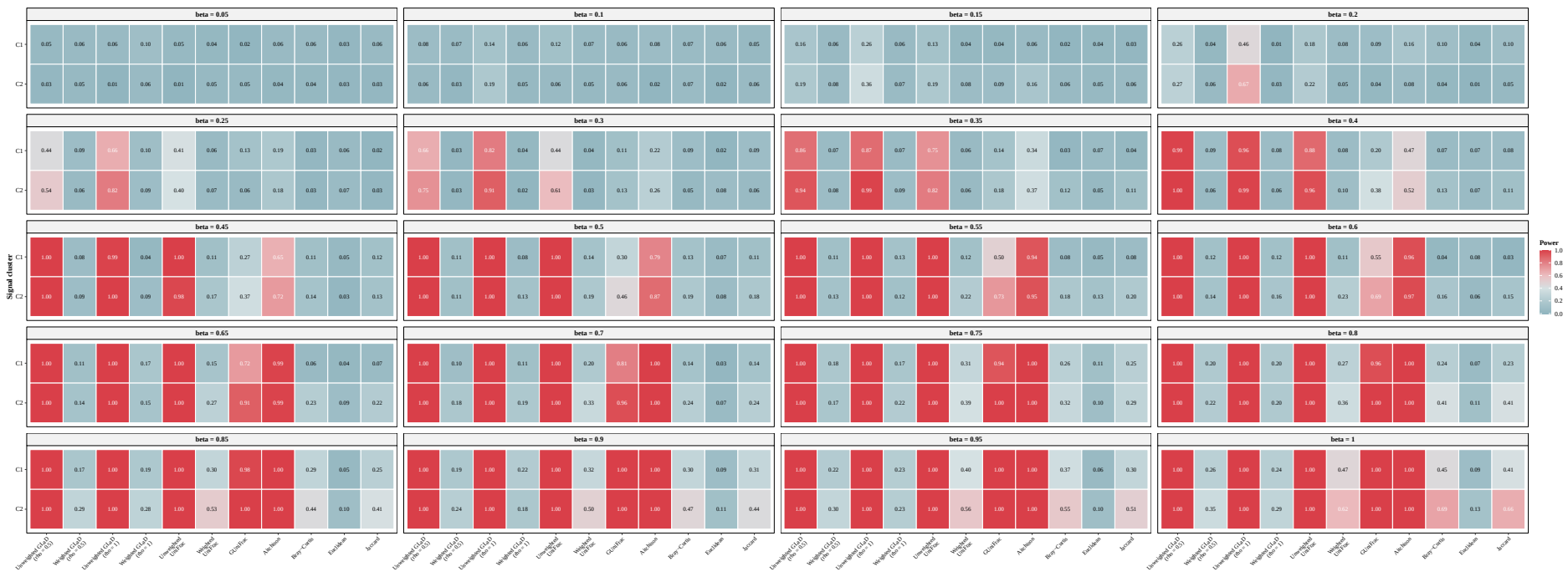

(b) Prevalence signal

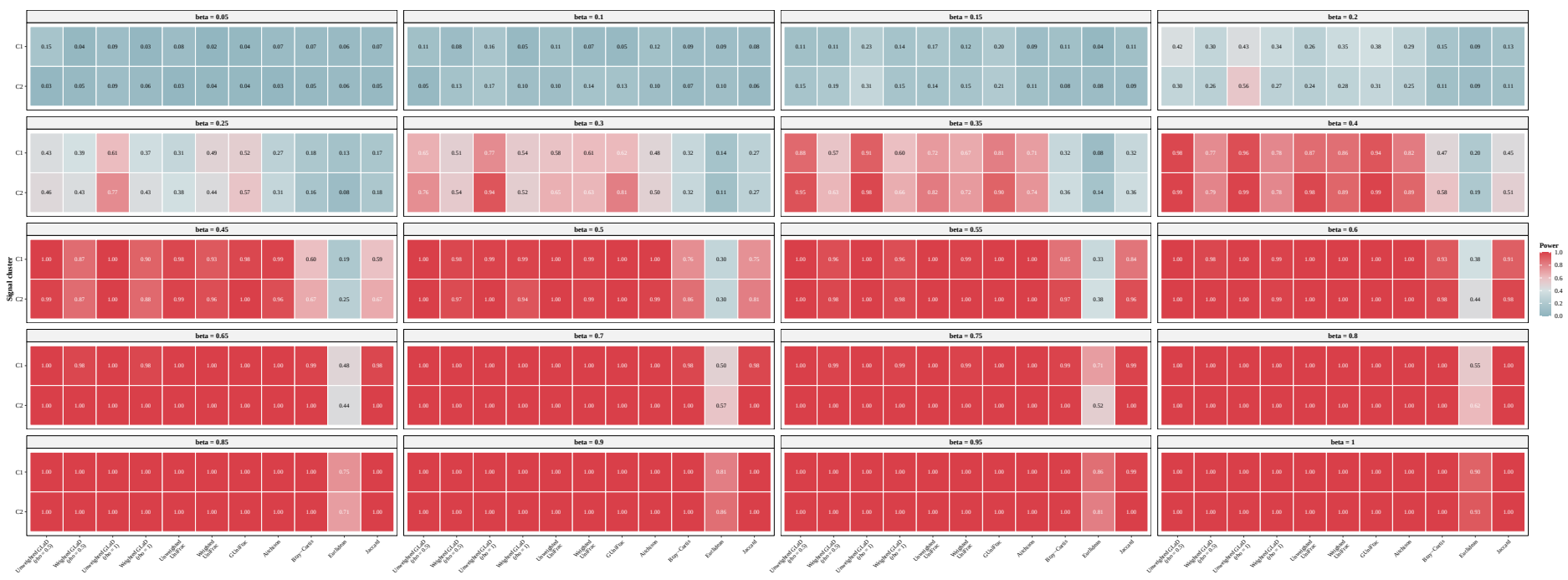

(c) Combined abundance and prevalence signal

Fig. S2: Power heatmaps for the simulation setting with $K = 2$. The three panels summarize power patterns under abundance, prevalence, and combined abundance–prevalence signal modes.

K	k	Number of OTUs in cluster	Overall abundance	Within cluster COP
2	1	404	563.222169	0.3593
2	2	212	311.495304	0.3719

Table S3: Cluster-level characteristics for the simulation setting with $K = 2$. For each cluster k, the table reports the number of OTUs assigned to the cluster, the overall abundance, and the mean within-cluster COP. Rows are ordered by the number of OTUs in each cluster from largest to smallest.

K	k	Number of OTUs in cluster	Overall abundance	Within cluster COP
4	2	257	323.056731	0.3305
4	4	212	311.495304	0.3719
4	1	93	133.450353	0.2895
4	3	54	106.715084	0.2426

Table S4: Cluster-level characteristics for the simulation setting with $K = 4$. For each cluster k, the table reports the number of OTUs assigned to the cluster, the overall abundance, and the mean within-cluster COP. Rows are ordered by the number of OTUs in each cluster from largest to smallest.

K	k	Number of OTUs in cluster	Overall abundance	Within cluster COP
6	6	212	311.495304	0.3719
6	3	109	113.329926	0.2787
6	1	93	133.450353	0.2895
6	2	82	76.682568	0.3118
6	4	66	133.044237	0.2469
6	5	54	106.715084	0.2426

Table S5: Cluster-level characteristics for the simulation setting with $K = 6$. For each cluster k, the table reports the number of OTUs assigned to the cluster, the overall abundance, and the mean within-cluster COP. Rows are ordered by the number of OTUs in each cluster from largest to smallest.

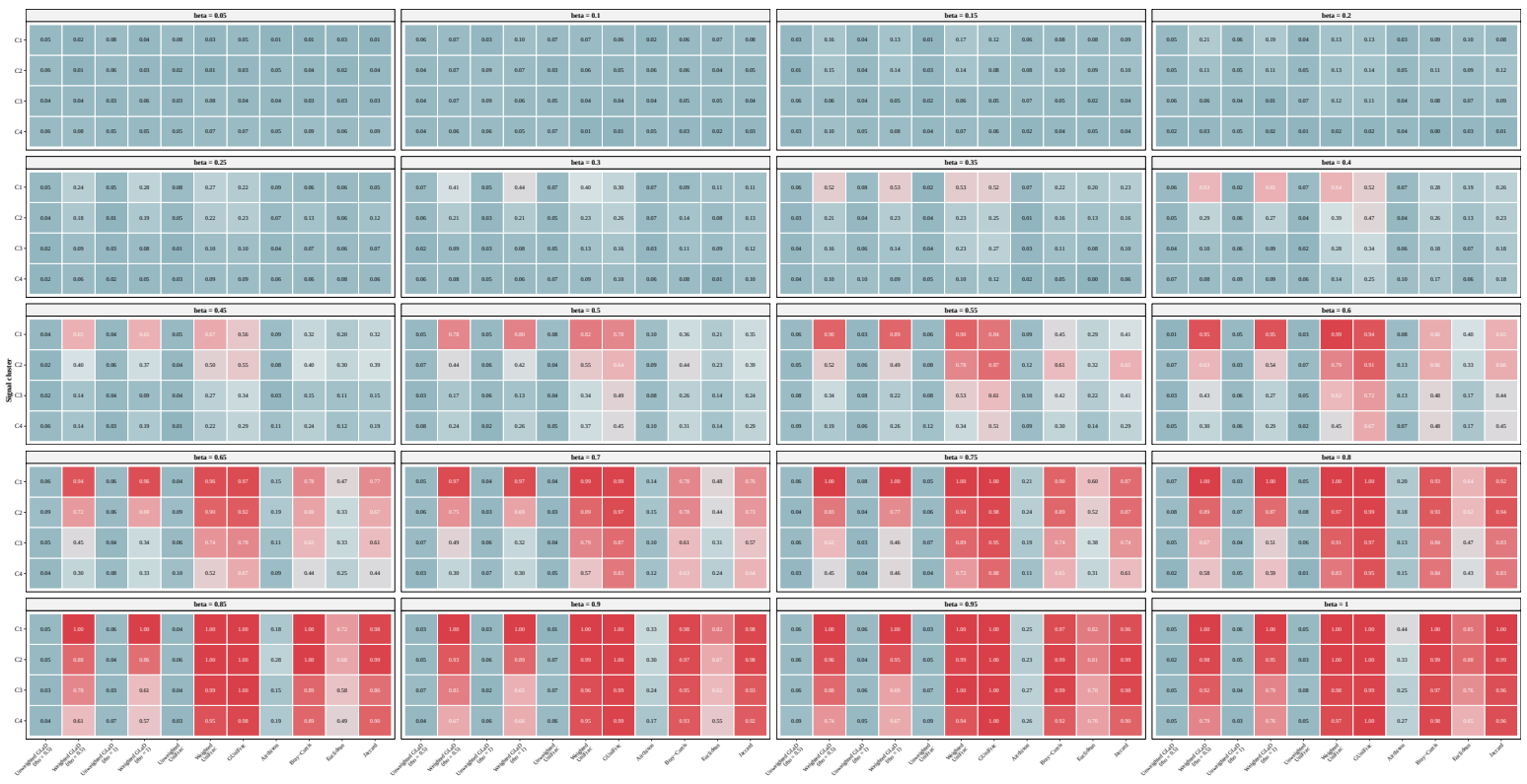

(a) Abundance signal

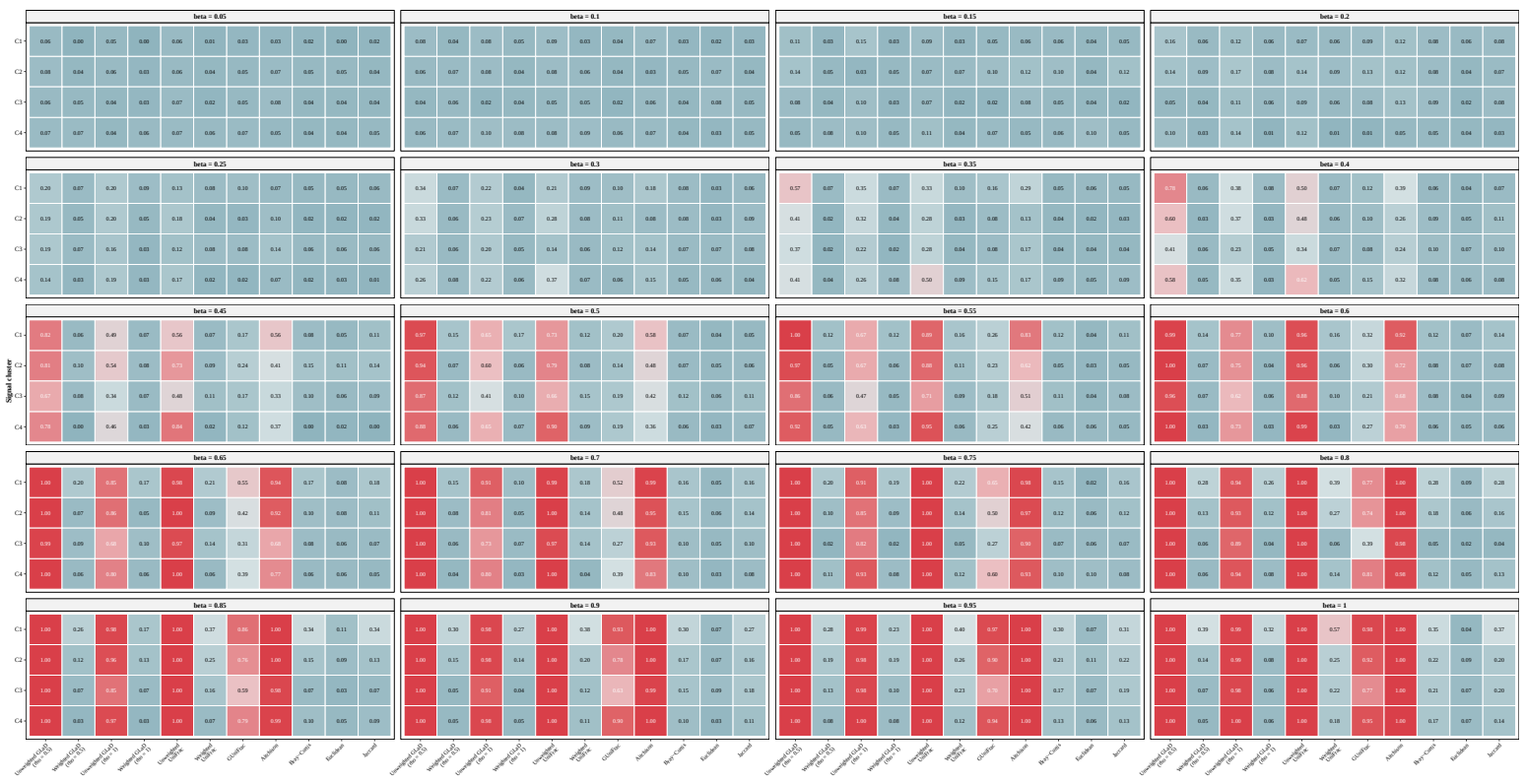

(b) Prevalence signal

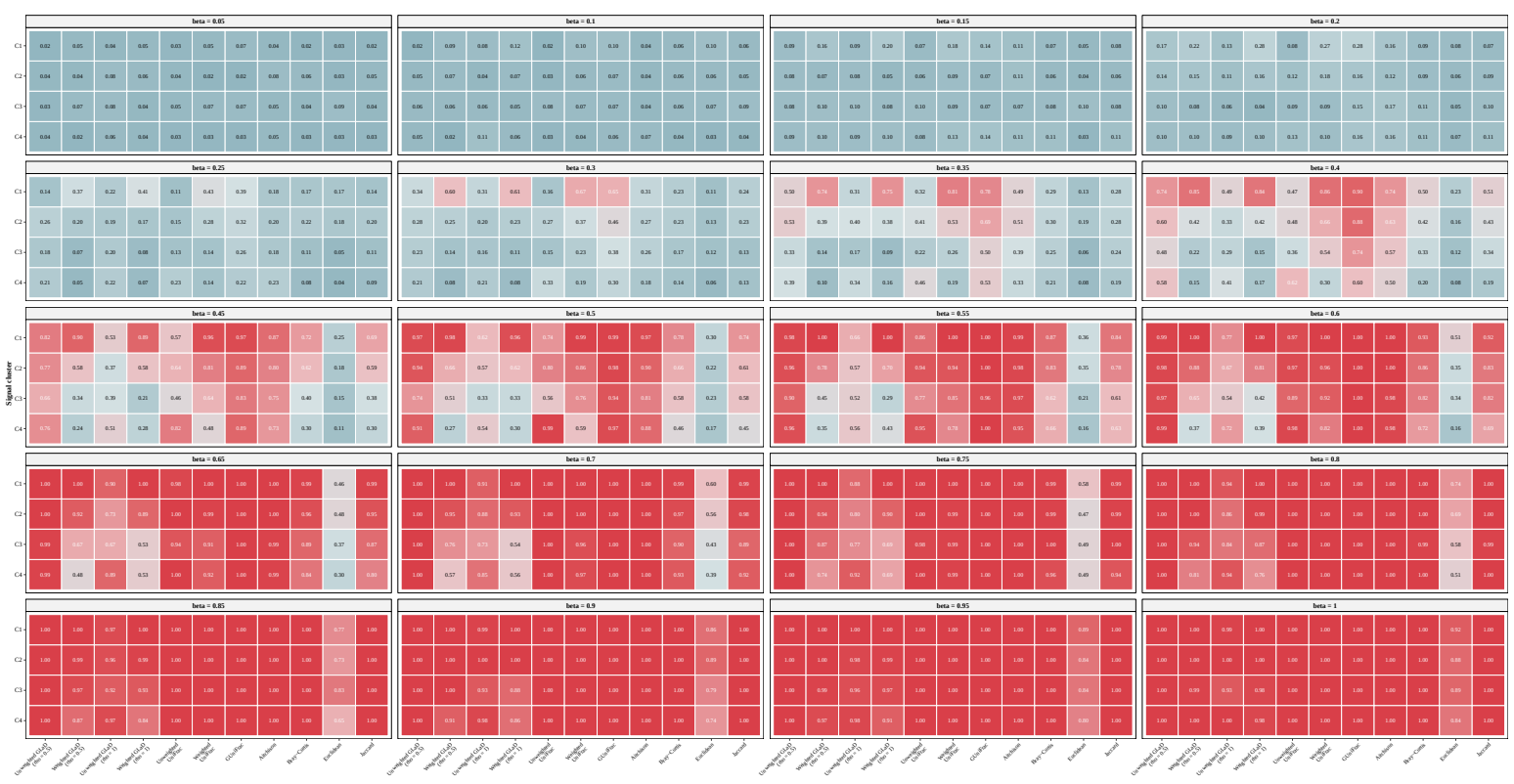

(c) Combined abundance and prevalence signal

Fig. S3: Power heatmaps for the simulation setting with $K = 4$. The three panels summarize power patterns under abundance, prevalence, and combined abundance–prevalence signal modes.

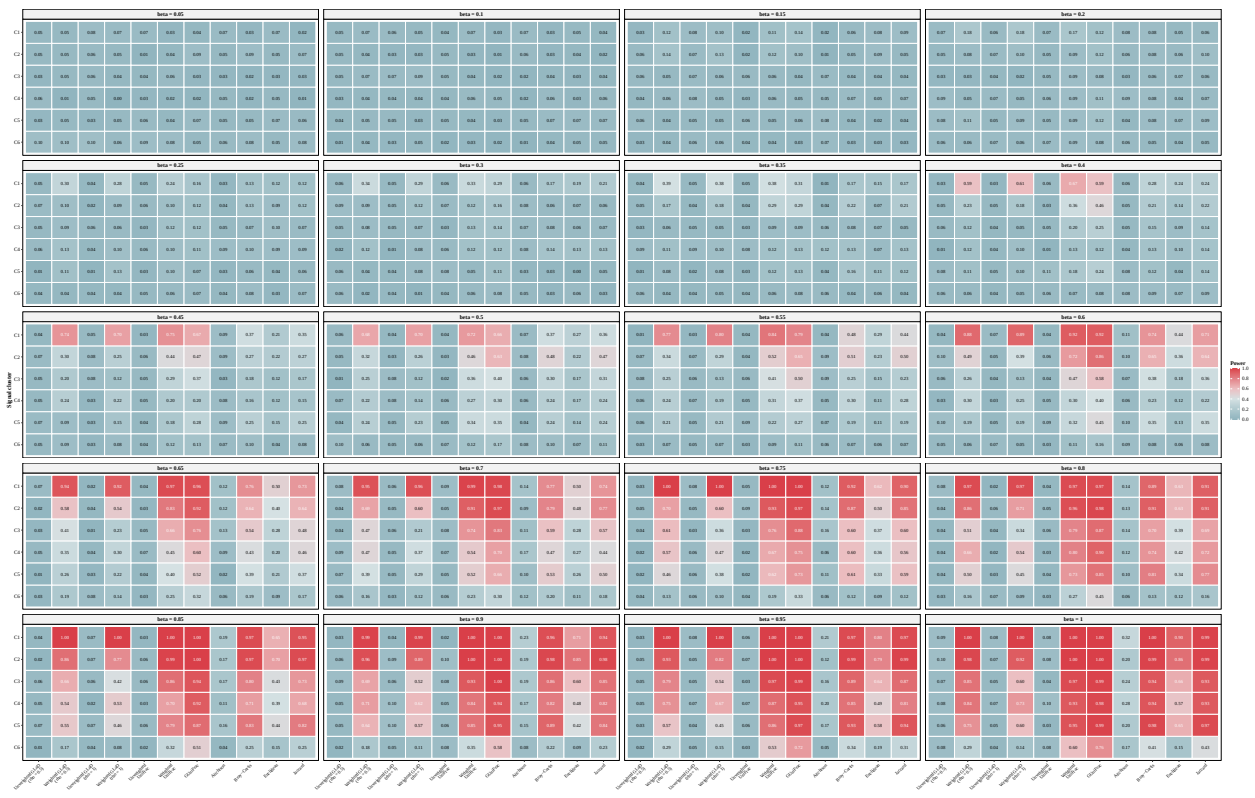

(a) Abundance signal

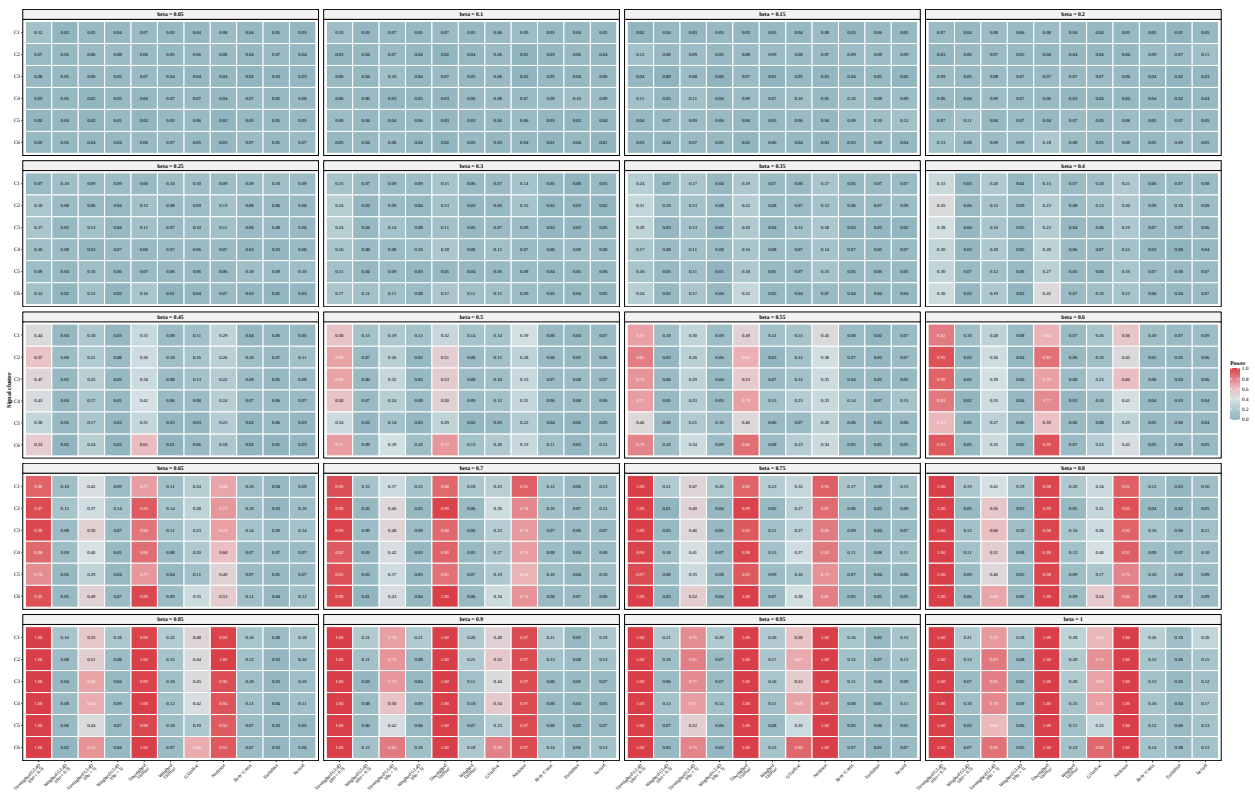

(b) Prevalence signal

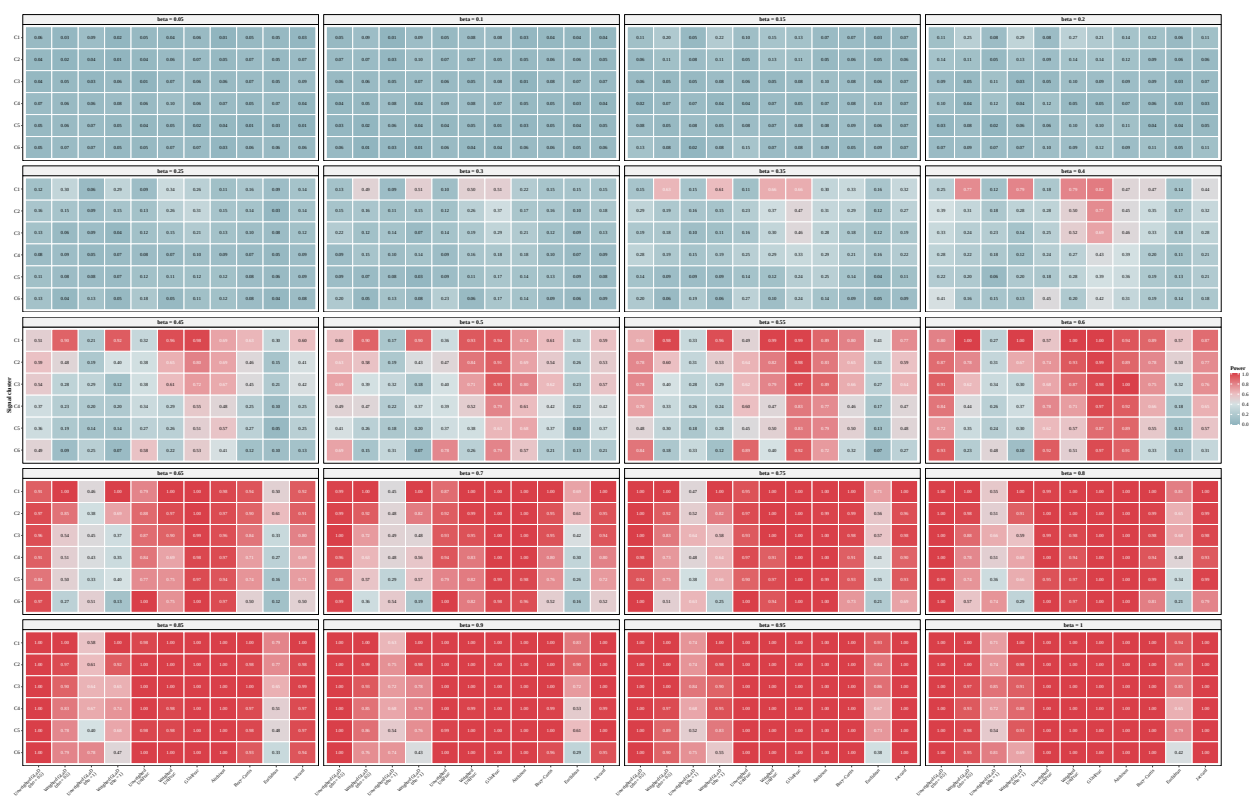

(c) Combined abundance and prevalence signal

Fig. S4: Power heatmaps for the simulation setting with $K = 6$. The three panels summarize power patterns under abundance, prevalence, and combined abundance–prevalence signal modes.

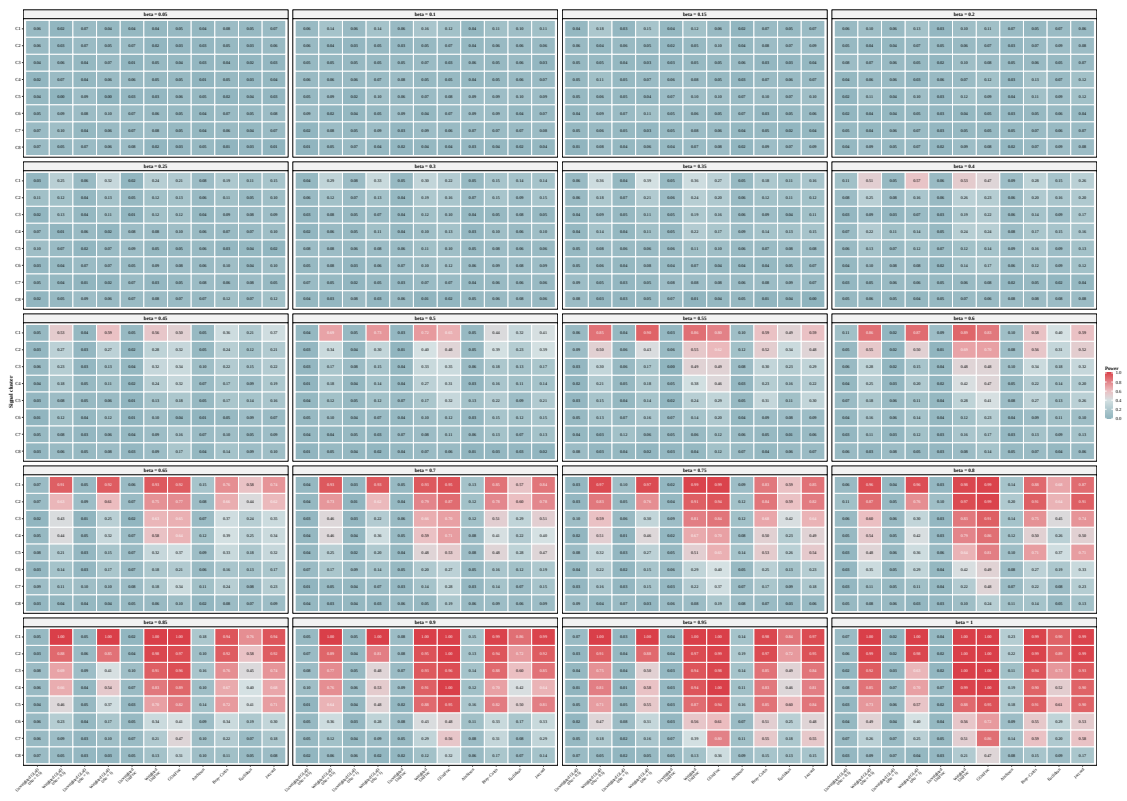

(a) Abundance signal

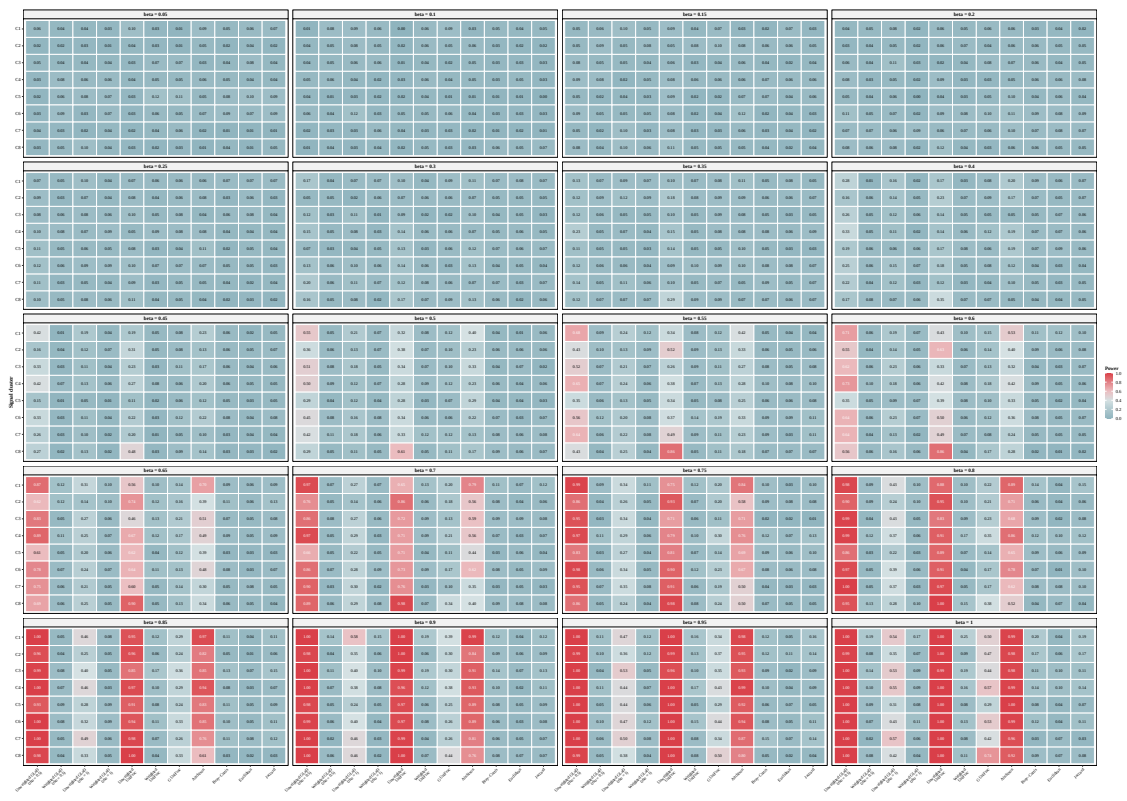

(b) Prevalence signal

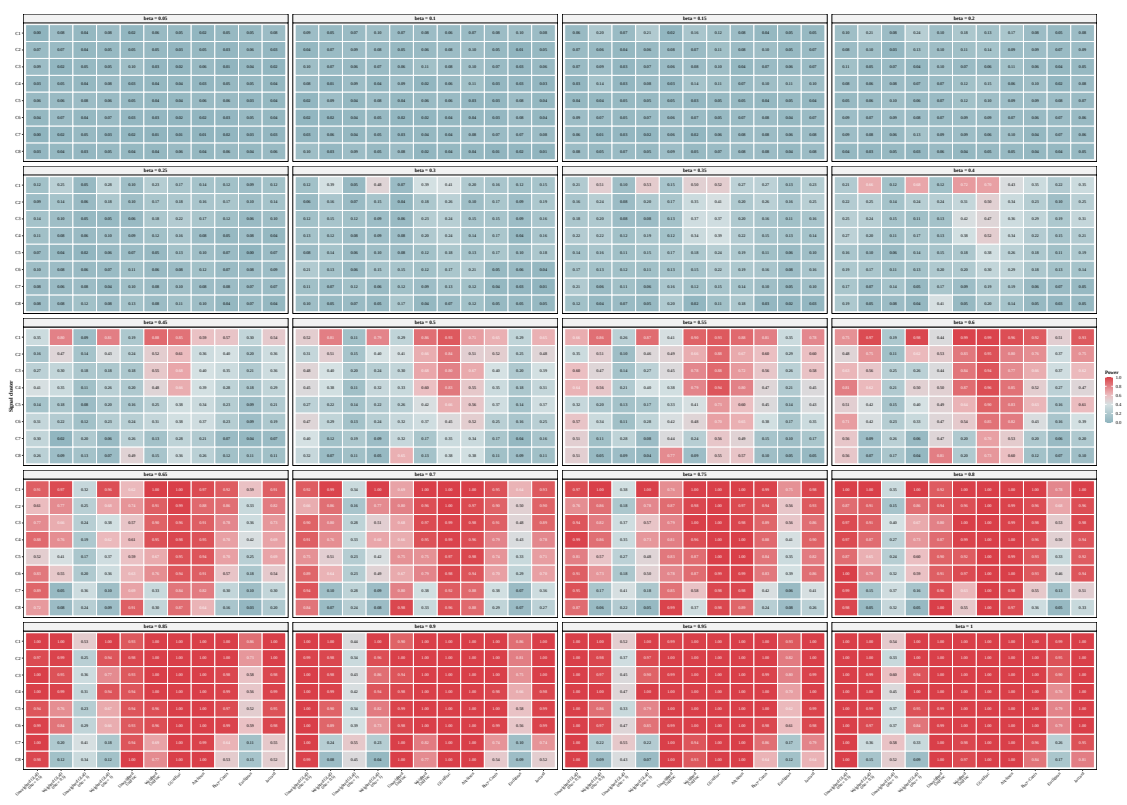

(c) Combined abundance and prevalence signal

Fig. S5: Power heatmaps for the simulation setting with $K = 8$. The three panels summarize power patterns under abundance, prevalence, and combined abundance–prevalence signal modes.

K	k	Number of OTUs in cluster	Overall abundance	Within cluster COP
8	8	171	304.252016	0.2661
8	1	93	133.450353	0.2895
8	2	90	84.970399	0.4777
8	3	68	38.404505	0.2534
8	4	66	133.044237	0.2469
8	7	54	106.715084	0.2426
8	5	53	68.784537	0.2421
8	6	21	5.096341	0.1945

Table S6: Cluster-level characteristics for the simulation setting with $K = 8$. For each cluster k, the table reports the number of OTUs assigned to the cluster, the overall abundance, and the mean within-cluster COP. Rows are ordered by the number of OTUs in each cluster from largest to smallest.

3 Real Data and Additional Analyses

3.1 Datasets

- **ORIGINS Oral Microbiome**: The *Oral Infections, Glucose Intolerance and Insulin Resistance* (ORIGINS) study is an occupation-based cohort established at Columbia University (2011–2013) to clarify how subgingival dysbiosis relates to early metabolic dysfunction. It enrolled 300 diabetes-free adults (20–55 years), performed detailed periodontal examinations, and collected subgingival plaque from the most posterior tooth in each quadrant. All 1,188 plaque specimens were screened for 11 sentinel periodontal pathogens using checkerboard DNA–DNA hybridization, while a nested subset of 152 participants (304 samples) was profiled with the Human Oral Microbe Identification Microarray (HOMIM), capturing relative abundances for 379 bacterial taxa. Parallel measurements of fasting glucose, insulin, and systemic inflammatory biomarkers make this a richly annotated benchmark for linking oral community composition to continuous host traits (21, 22). The original dataset is available on Qiita under study 11808.
- **COVID-Gut Microbiome**: This cross-sectional stool dataset comprises 60 adults—20 with acute SARS-CoV-2 infection, 20 recovered ($\geq$ 14 days post-clearance), and 20 uninfected controls—recruited at Robert Wood Johnson University Hospital (New Brunswick, NJ) between May 2020 and January 2021. Fecal DNA was extracted, the V4 region of the bacterial 16S rRNA gene was amplified in triplicate, and libraries were sequenced on an Illumina MiSeq. The resulting profiles, together with rich clinical metadata (age, comorbidities, antibiotic exposure, GI symptoms, etc.), enable interrogation of gut-microbiota shifts across the infection–recovery continuum (23). The original dataset is available on Qiita under study 14812.
- **HIV-Gut Microbiome**: This dataset comprises stool 16S-rRNA profiles from 113 Denver-area men—22 heterosexual controls, 32 HIV-negative MSM, 14 untreated HIV-positive MSM, and 45 ART-treated HIV-positive MSM (25 with lipodystrophy). Self-collected fecal swabs were processed with the PowerSoil kit, the V4 region (515F/806R) was sequenced on an Illumina MiSeq, and extensive clinical metadata (lipids, glucose, cytokines, diet, metabolomics) accompany the microbiome reads, facilitating multi-omic analyses of gut dysbiosis and metabolic risk in HIV infection (24). The original dataset is available on Qiita under study 13338.
- **IBD Dataset**: This stool-based resource integrates five complementary 'omics layers—16S-rRNA amplicons, shotgun metagenomics, metaproteomics, metabolomics and host SNP arrays—from 210 participants (117 Crohn's disease, 73 ulcerative colitis, 20 non-IBD controls) enrolled at a single IBD center. Detailed clinical metadata (endoscopic scores, histology, medications, diet) permit location-specific analyses of ileal versus colonic Crohn's disease and ulcerative colitis severity (25, 26). In the present study, we analyze both the 16S amplicon and whole-genome shotgun (WGS) sequencing components of this dataset. The original dataset is available on Qiita under study 12675.
- **Pig Gut Microbiome**: This longitudinal dataset follows 30 juvenile female Göttingen minipigs that were fed ad libitum for 20 weeks on either a high-fat

"high-risk" diet containing 20% fructose (n = 15) or an isocaloric "lower-risk" diet in which the same carbohydrate load was supplied as Hi-Maize resistant starch (n = 15). Fecal samples collected at weeks 4, 12, and 20 were subjected to 16S rRNA amplicon sequencing, with matched plasma, urine, and fecal metabolomics, providing a controlled model of diet-induced shifts in gut microbiota and host metabolism (27). The original dataset is available on Qiita under study 13396.

3.2 Bioinformatics Processing

The 16S rRNA reads were processed with QIIME 2 (v2024.10) (28) to generate amplicon sequence variant (ASV) counts, taxonomic assignments, and a phylogenetic tree. Quality control and denoising were performed with DADA2 (29), guided by quality score profiles obtained in QIIME 2. Taxonomy was assigned against SILVA (v138.2) (30), and the ASV phylogeny was inferred with FastTree (31). For shotgun metagenomes, operational genomic unit (OGU) profiles were generated with Woltka (32) using the Web of Life (WoL) reference database and phylogeny (33). The phylogenetic tree was obtained by pruning the WoL tree to observed genomes with the `ape` package in R (34).

3.3 Results of Evaluation Indices

The evaluation criteria and rank aggregation are defined in Section 1.6. This subsection reports the numerical diagnostics; the radar and tournament summaries follow in Section 3.4.

As summarized in Table S7, we benchmark how well common β-diversity measures admit faithful two-dimensional PCoA representations across multiple microbiome datasets. Reported diagnostics are the fraction of negative inertia (FNI), trustworthiness, continuity, the Mantel correlation r with its permutation p-value, and Kruskal stress; lower FNI/stress and higher trustworthiness/continuity/Mantel r indicate better structure preservation.

As summarized in Table S8, we assess the quality of k-medoids clustering on the same two-dimensional PCoA embeddings. Reported indices are the Rand index and adjusted Rand index (pairwise agreement), average silhouette width, the Davies–Bouldin index (DBI), the Calinski–Harabasz score (CH), and the Dunn index. Larger RI/ARI/silhouette/CH/Dunn and smaller DBI indicate better-separated clusters.

data name	method	FNI	trustworthiness	continuity	mantel_r	mantel_p	stress
Covid_gut(14812)	wunifrac	0.05304	0.675	0.76156	0.96041	0.001	0.36617
Covid_gut(14812)	unifrac	0	0.45375	0.57563	0.65947	0.001	0.71772
Covid_gut(14812)	gunifrac	0	0.65094	0.73594	0.7856	0.001	0.56385
Covid_gut(14812)	glad	0	0.77438	0.89312	0.91028	0.001	0.34191
Covid_gut(14812)	glad0	0	0.4475	0.63438	0.80771	0.001	0.58066
Covid_gut(14812)	euc	0	0.52062	0.70406	0.80139	0.001	0.58061
Covid_gut(14812)	aitchison	0	0.38875	0.48281	0.63531	0.001	0.70308
Covid_gut(14812)	bray	9E-05	0.55031	0.65219	0.66245	0.001	0.5983
Covid_gut(14812)	jaccard	0	0.52406	0.59844	0.54519	0.001	0.71719
HIV_gut(13338)	wunifrac	0.10897	0.52644	0.68	0.76357	0.001	0.61112
HIV_gut(13338)	unifrac	0.06026	0.4557	0.55173	0.55126	0.001	0.68938
HIV_gut(13338)	gunifrac	0.08783	0.62197	0.73998	0.70951	0.001	0.59822
HIV_gut(13338)	glad	0	0.73273	0.86147	0.9171	0.001	0.35205
HIV_gut(13338)	glad0	0	0.61968	0.69949	0.92204	0.001	0.427
HIV_gut(13338)	euc	0	0.57823	0.74313	0.96867	0.001	0.28253
HIV_gut(13338)	aitchison	0	0.58976	0.74902	0.9368	0.001	0.36764
HIV_gut(13338)	bray	0.03113	0.55097	0.63283	0.45375	0.001	0.66854
HIV_gut(13338)	jaccard	0	0.52091	0.58048	0.33042	0.001	0.79732
IBD_16s_data_V4	wunifrac	0.17273	0.45692	0.30665	0.93366	0.001	0.32986
IBD_16s_data_V4	unifrac	0	0.52751	0.55829	0.37551	0.001	0.73186
IBD_16s_data_V4	gunifrac	0.03598	0.72208	0.81883	0.72185	0.001	0.54888
IBD_16s_data_V4	glad	0	0.76571	0.87839	0.89806	0.001	0.33971
IBD_16s_data_V4	glad0	0	0.54061	0.73181	0.71011	0.001	0.56656
IBD_16s_data_V4	euc	0	0.53836	0.72996	0.72077	0.001	0.56079
IBD_16s_data_V4	aitchison	0	0.3582	0.34629	0.51703	0.001	0.69548
IBD_16s_data_V4	bray	0.045	0.53329	0.61942	0.40157	0.001	0.63519
IBD_16s_data_V4	jaccard	0	0.50841	0.52854	0.27925	0.001	0.77032
IBD_wgs_data_V4	wunifrac	0.10546	0.47989	0.51393	0.82875	0.001	0.60913
IBD_wgs_data_V4	unifrac	0	0.67865	0.78393	0.89116	0.001	0.60352
IBD_wgs_data_V4	gunifrac	0.01643	0.76116	0.86311	0.76126	0.001	0.50894
IBD_wgs_data_V4	glad	0	0.83724	0.93191	0.80539	0.001	0.32234
IBD_wgs_data_V4	glad0	0	0.47131	0.65035	0.57871	0.001	0.67799
IBD_wgs_data_V4	euc	0	0.40193	0.67699	0.79917	0.001	0.64162
IBD_wgs_data_V4	aitchison	0	0.72034	0.81322	0.85245	0.001	0.56643
IBD_wgs_data_V4	bray	0.04002	0.56757	0.71453	0.46501	0.001	0.61295
IBD_wgs_data_V4	jaccard	0	0.55667	0.67571	0.37283	0.001	0.75527
physeq_tumor	wunifrac	0.03422	0.62406	0.78277	0.83072	0.001	0.48763
physeq_tumor	unifrac	0	0.76382	0.77908	0.73918	0.001	0.60303
physeq_tumor	gunifrac	0	0.65414	0.77716	0.71321	0.001	0.60306
physeq_tumor	glad	0	0.56317	0.75416	0.91007	0.001	0.42767
physeq_tumor	glad0	0	0.38221	0.51194	0.74721	0.001	0.71321
physeq_tumor	euc	0	0.31107	0.47501	0.8763	0.001	0.5841
physeq_tumor	aitchison	0	0.28697	0.54666	0.71559	0.001	0.71473
physeq_tumor	bray	0	0.51209	0.66704	0.3947	0.001	0.69488
physeq_tumor	jaccard	0	0.50413	0.62465	0.33734	0.001	0.79605
Pig_gut(13396)	wunifrac	0.05267	0.79935	0.89126	0.87602	0.001	0.39789
Pig_gut(13396)	unifrac	0	0.76888	0.80848	0.55109	0.001	0.66722
Pig_gut(13396)	gunifrac	0	0.72635	0.84003	0.67258	0.001	0.56419
Pig_gut(13396)	glad	0	0.68463	0.80952	0.9285	0.001	0.44594
Pig_gut(13396)	glad0	0	0.48999	0.68099	0.87495	0.001	0.6577
Pig_gut(13396)	euc	0	0.37586	0.62245	0.83758	0.001	0.62724
Pig_gut(13396)	aitchison	0	0.64366	0.7153	0.59592	0.001	0.68997
Pig_gut(13396)	bray	2E-04	0.59818	0.75104	0.56904	0.001	0.63696
Pig_gut(13396)	jaccard	0	0.58619	0.73915	0.51612	0.001	0.76501

Table S7: Structure-preservation statistics for two-dimensional PCoA embeddings computed from each beta-diversity measure ("method") on the listed microbiome datasets (dataset name followed by sample size): fraction of negative inertia (FNI), trustworthiness, continuity, Mantel correlation coefficient r with permutation p-value, and Kruskal stress.

data_name	method	rand_index	adjusted_rand_index	avg_silhouette	dbi	chi	dunn
Covid_gut(14812)	wunifrac	0.48718	-0.02524	0.19703	1.09579	15.47119	0.0248
Covid_gut(14812)	unifrac	0.48846	-0.01249	0.02593	0.65423	19.68569	0.10058
Covid_gut(14812)	gunifrac	0.49231	-0.01605	0.08453	1.10164	21.01508	0.00353
Covid_gut(14812)	glad	0.49231	-0.01579	0.19657	0.99563	23.73002	0.02621
Covid_gut(14812)	glad0	0.51923	0.0384	0.08283	1.27235	11.50731	0.01049
Covid_gut(14812)	euc	0.53333	0.0663	0.08723	0.98169	16.10409	0.01716
Covid_gut(14812)	aitchison	0.51923	0.03789	0.02753	1.34829	12.52303	0.01796
Covid_gut(14812)	bray	0.49872	-0.00263	0.08721	0.93208	32.31962	0.1058
Covid_gut(14812)	jaccard	0.49872	-0.00263	0.02849	0.99188	28.97241	0.13505
HIV_gut(13338)	wunifrac	0.50723	-0.01048	-0.02462	3.26339	11.74324	0.00012
HIV_gut(13338)	unifrac	0.56989	0.13723	0.0205	6.47316	6.57593	0.00371
HIV_gut(13338)	gunifrac	0.58345	0.15997	0.0774	1.08063	157.75257	0.00307
HIV_gut(13338)	glad	0.5105	-0.03832	0.50336	0.48521	554.40828	0.08461
HIV_gut(13338)	glad0	0.50964	-0.03738	0.52683	0.40991	889.8354	0.02841
HIV_gut(13338)	euc	0.51862	-0.04127	0.7776	0.40389	812.78894	0.02316
HIV_gut(13338)	aitchison	0.5088	-0.04382	0.62674	0.5252	752.84633	0.02363
HIV_gut(13338)	bray	0.49989	-0.00886	0.04565	1.55581	117.04479	0.00152
HIV_gut(13338)	jaccard	0.5164	-0.03754	0.02458	0.4317	238.73245	0.0155
IBD_16s_data_V4	wunifrac	0.51828	-0.00937	0.78704	0.43049	289.73688	0.18065
IBD_16s_data_V4	unifrac	0.50102	0.00159	0.02835	1.4951	70.22947	0.00291
IBD_16s_data_V4	gunifrac	0.49753	-0.01913	0.1157	1.08714	83.06246	0.01261
IBD_16s_data_V4	glad	0.49736	-0.02838	0.39921	0.6754	168.39699	0.04528
IBD_16s_data_V4	glad0	0.50015	-0.02752	0.33231	0.47538	324.27231	0.00891
IBD_16s_data_V4	euc	0.50102	-0.02833	0.41039	0.48056	298.53138	0.06116
IBD_16s_data_V4	aitchison	0.50869	-0.00751	0.09961	1.0304	56.97075	0.00075
IBD_16s_data_V4	bray	0.51037	0.01988	0.07918	1.19389	105.62038	0.00232
IBD_16s_data_V4	jaccard	0.51037	0.01578	0.03315	1.06572	127.63443	0.00368
IBD_wgs_data_V4	wunifrac	0.50376	0.00135	0.07159	4.2819	3.34045	0.00086
IBD_wgs_data_V4	unifrac	0.52444	0.04706	0.092	1.15945	82.87718	0.00349
IBD_wgs_data_V4	gunifrac	0.50376	0.00013	0.17465	0.83116	165.46538	0.04319
IBD_wgs_data_V4	glad	0.50259	-0.00762	0.34251	0.69479	171.44444	0.05337
IBD_wgs_data_V4	glad0	0.498	-0.02528	0.20272	0.75884	170.81411	0.02562
IBD_wgs_data_V4	euc	0.50646	-0.02398	0.33142	0.61554	21.31483	0.00467
IBD_wgs_data_V4	aitchison	0.49847	-0.01381	0.1608	0.81328	201.86465	0.0486
IBD_wgs_data_V4	bray	0.50646	0.0108	0.11779	0.90767	167.73488	0.00211
IBD_wgs_data_V4	jaccard	0.49976	-0.01171	0.03928	0.89675	126.29715	0.00026
physeq_tumor	wunifrac	0.55575	0.1149	0.18808	1.08838	33.40134	0.05113
physeq_tumor	unifrac	0.55575	0.11139	0.07142	1.07053	48.44519	0.0517
physeq_tumor	gunifrac	0.6194	0.23887	0.05	1.53309	21.57186	0.03036
physeq_tumor	glad	0.52063	0.04355	0.21612	0.93177	29.75925	0.00659
physeq_tumor	glad0	0.49956	0.00912	0.51167	0.33714	61.22909	0.36242
physeq_tumor	euc	0.49298	-0.00327	0.85001	0.01853	186.95445	1.57587
physeq_tumor	aitchison	0.50351	0.01633	0.19541	0.36789	29.61553	0.28387
physeq_tumor	bray	0.53644	0.07364	0.03626	1.32645	24.04504	0.01512
physeq_tumor	jaccard	0.50351	0.01406	0.02081	0.8066	36.54715	0.0497
Pig_gut(13396)	wunifrac	0.85493	0.70984	0.22622	0.78208	93.8715	0.01383
Pig_gut(13396)	unifrac	0.57528	0.1505	0.04557	1.24945	43.63204	0.01122
Pig_gut(13396)	gunifrac	0.93483	0.86965	0.13485	0.74303	115.29834	0.11005
Pig_gut(13396)	glad	0.80025	0.60059	0.20249	0.7877	38.51621	0.00347
Pig_gut(13396)	glad0	0.49663	0.003	0.50192	0.35774	61.23118	0.06125
Pig_gut(13396)	euc	0.50062	0.01001	0.45608	0.51217	61.79068	0.12855
Pig_gut(13396)	aitchison	0.61523	0.23192	0.04709	1.1221	35.27153	0.00839
Pig_gut(13396)	bray	0.80025	0.60053	0.07977	0.96726	71.26443	0.02962
Pig_gut(13396)	jaccard	0.80025	0.60053	0.02604	1.00307	65.92149	0.00385

Table S8: Clustering-quality indices for k-medoids applied to the same embeddings: Rand index, adjusted Rand index, average silhouette width, Davies–Bouldin index, Calinski–Harabasz score, and Dunn index for each beta-diversity measure and dataset.

	FNI	trustworthiness	continuity	mantel_r	mantel_p	stress	avg_silhouette	dbi	chi	dunn
wunifrac	0.11145	0.9394	0.95476	0.95355	0.001	0.20978	0.28959	0.93168	63.38944	0.09192
unifrac	0.01801	0.7322	0.8157	0.79707	0.001	0.52447	0.11343	1.00585	49.60956	0.05142
gunifrac	0.02537	0.78859	0.88502	0.87954	0.001	0.4144	0.20394	0.80802	85.52546	0.06063
glad	0	0.94846	0.96354	0.98403	0.001	0.13701	0.34509	0.90912	68.22812	0.10141
glad0	0	0.91816	0.93096	0.9529	0.001	0.25915	0.3118	0.82568	67.38087	0.04844
euc	0	0.95427	0.96001	0.9727	0.001	0.20902	0.36631	0.85319	56.03228	0.0637
aitchison	0	0.53933	0.50917	0.56359	0.001	0.66681	0.07457	0.70732	96.42857	0.03072
bray	0.02742	0.82352	0.86849	0.81308	0.001	0.44001	0.19329	0.81536	75.72828	0.0821
jaccard	0	0.75614	0.79834	0.79076	0.001	0.65864	0.06852	0.80789	72.09637	0.03128

Table S9: **Embedding and clustering diagnostics for the mouse gut dataset.** Summary of Euclidean/embedding consistency (FNI; smaller is better), neighborhood preservation (trustworthiness, continuity), global concordance (Mantel r and p), ordination fit (stress), and clustering quality metrics (average silhouette, Davies–Bouldin index, χ statistic, and Dunn index) across the compared dissimilarity measures.

3.4 Radar and Tournament Summaries

The radar plot in Fig. S6 summarizes the Euclidean-property assessment across the same eight microbiome datasets reported in Table 1 of the main text. Each radial count is obtained directly by counting the datasets marked as Euclidean for that method in the table. The tournament plots in Figs. S7–S9 display rankings across individual criteria and the Borda aggregate for six datasets.

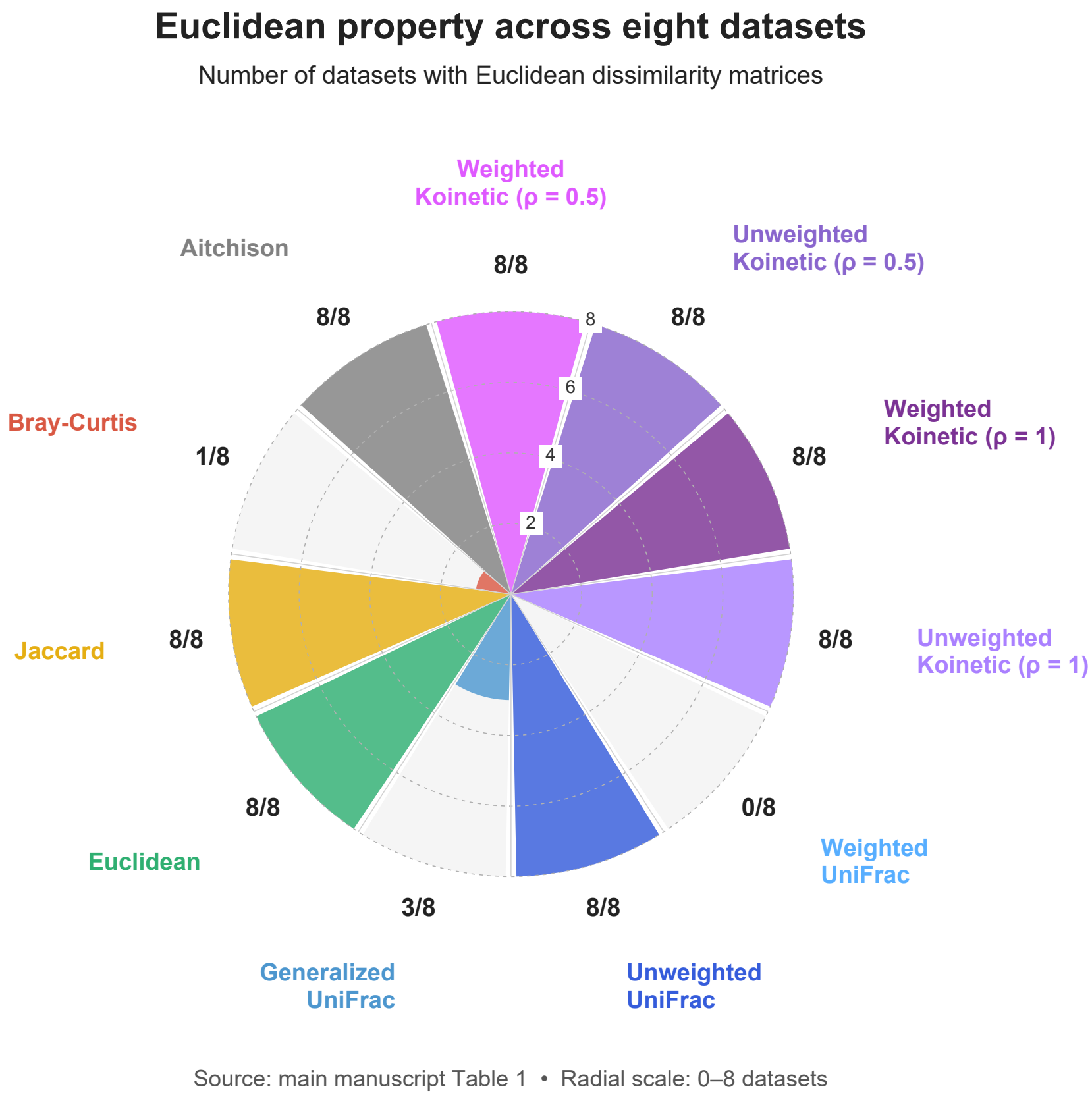

Fig. S6: **Euclidean-property assessment across eight microbiome datasets.** The radial extent of each colored sector gives the number of datasets (0–8) for which the corresponding dissimilarity matrix satisfies the Euclidean property. Counts are computed directly from Table 1 of the main text; Koinetic denotes the GLaD variants listed there. The outer radius represents eight of eight datasets. These counts describe the matrices observed in this benchmark and do not assert universal Euclidean validity for the comparison methods.

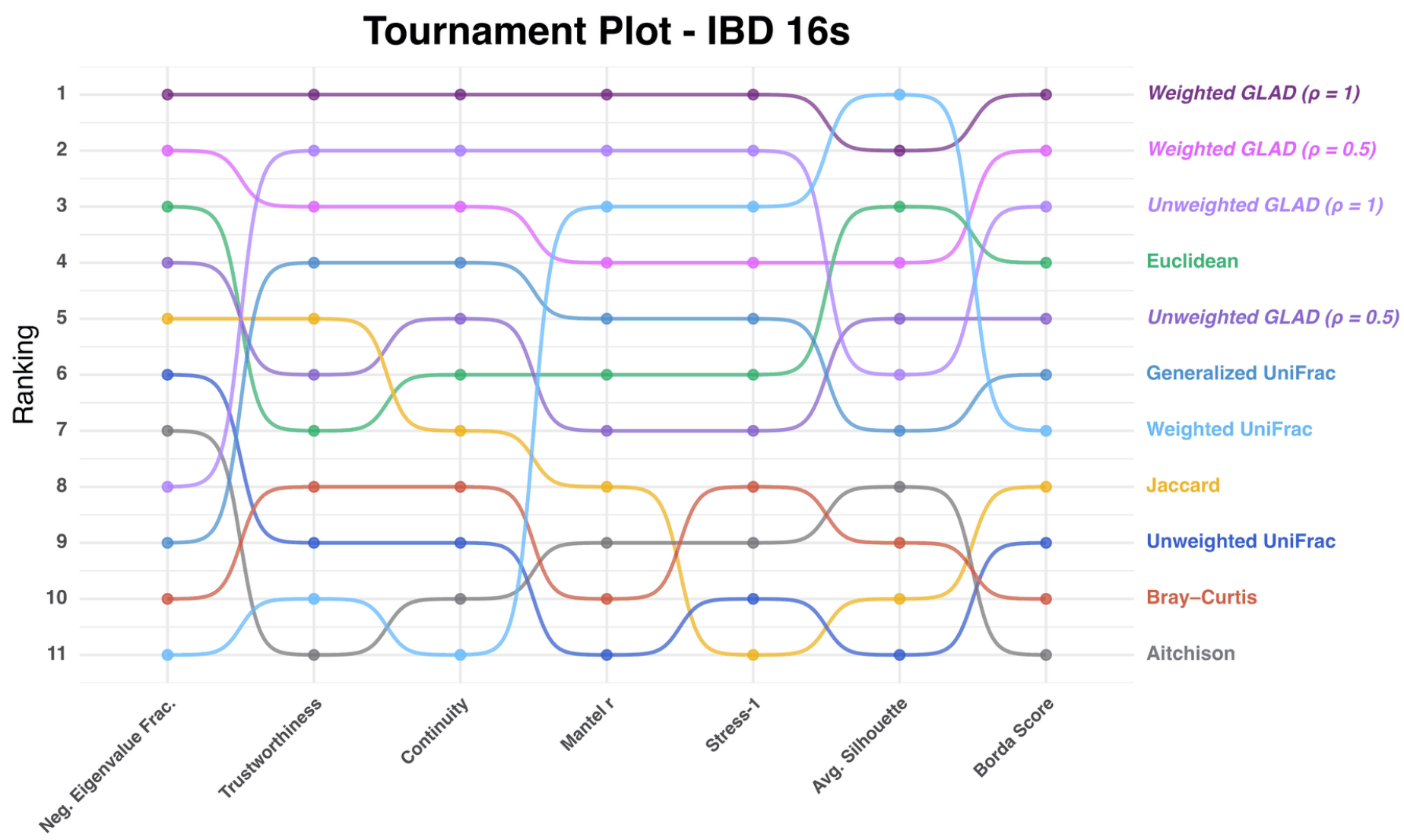

(a) IBD cohort, 16S rRNA sequencing

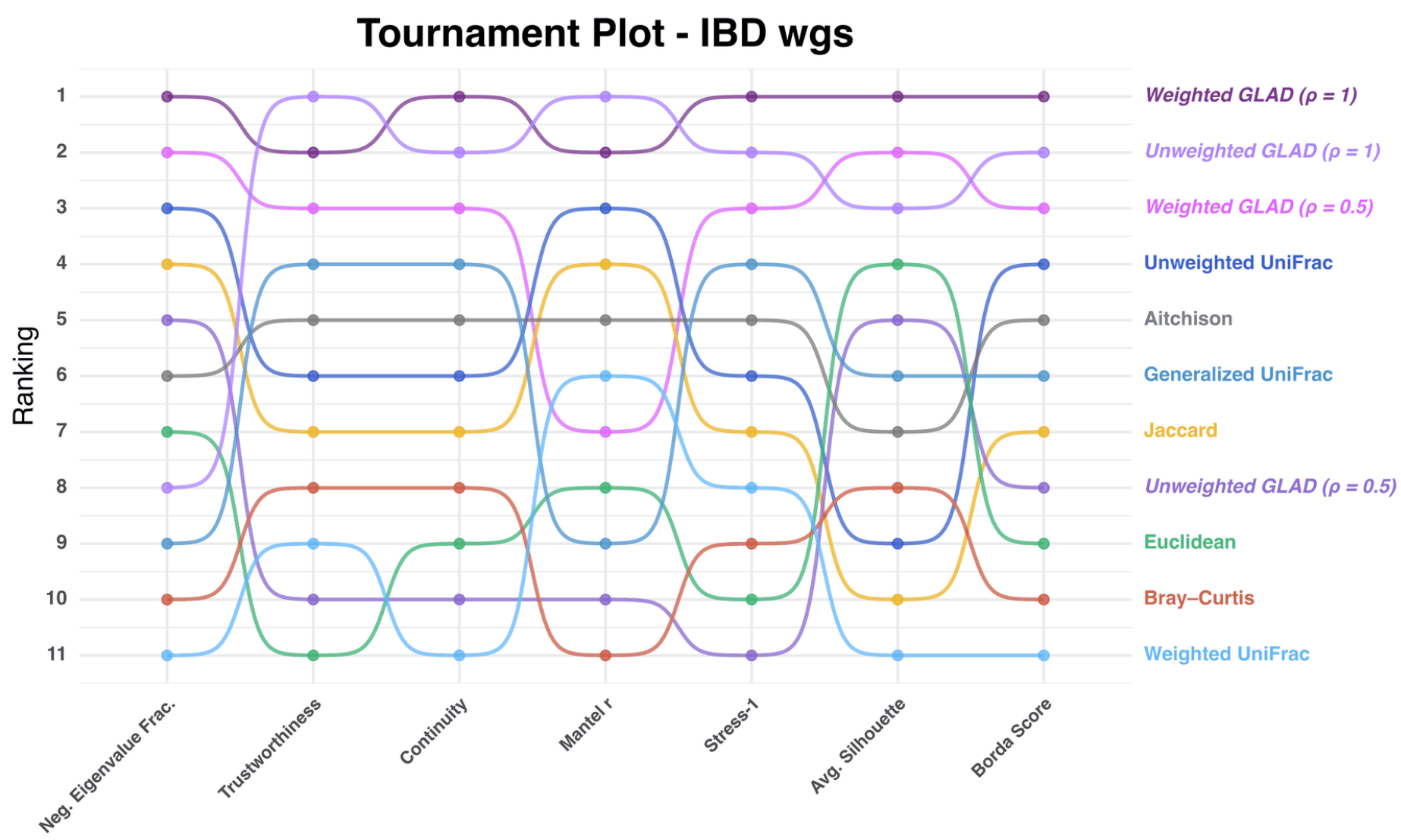

(b) IBD cohort, whole-genome shotgun sequencing

Fig. S7: **Tournament-style rank aggregation of β-diversity measures in the IBD cohort.** Colored trajectories follow the ranks of eleven dissimilarities across six evaluation criteria (negative eigenvalue fraction, trustworthiness, continuity, Mantel correlation, stress, and average silhouette) and the aggregate Borda score. Rank 1 is best for each column. Purple and pink trajectories identify the four GLaD variants ($\rho \in \{0.5, 1\}$, weighted or unweighted).

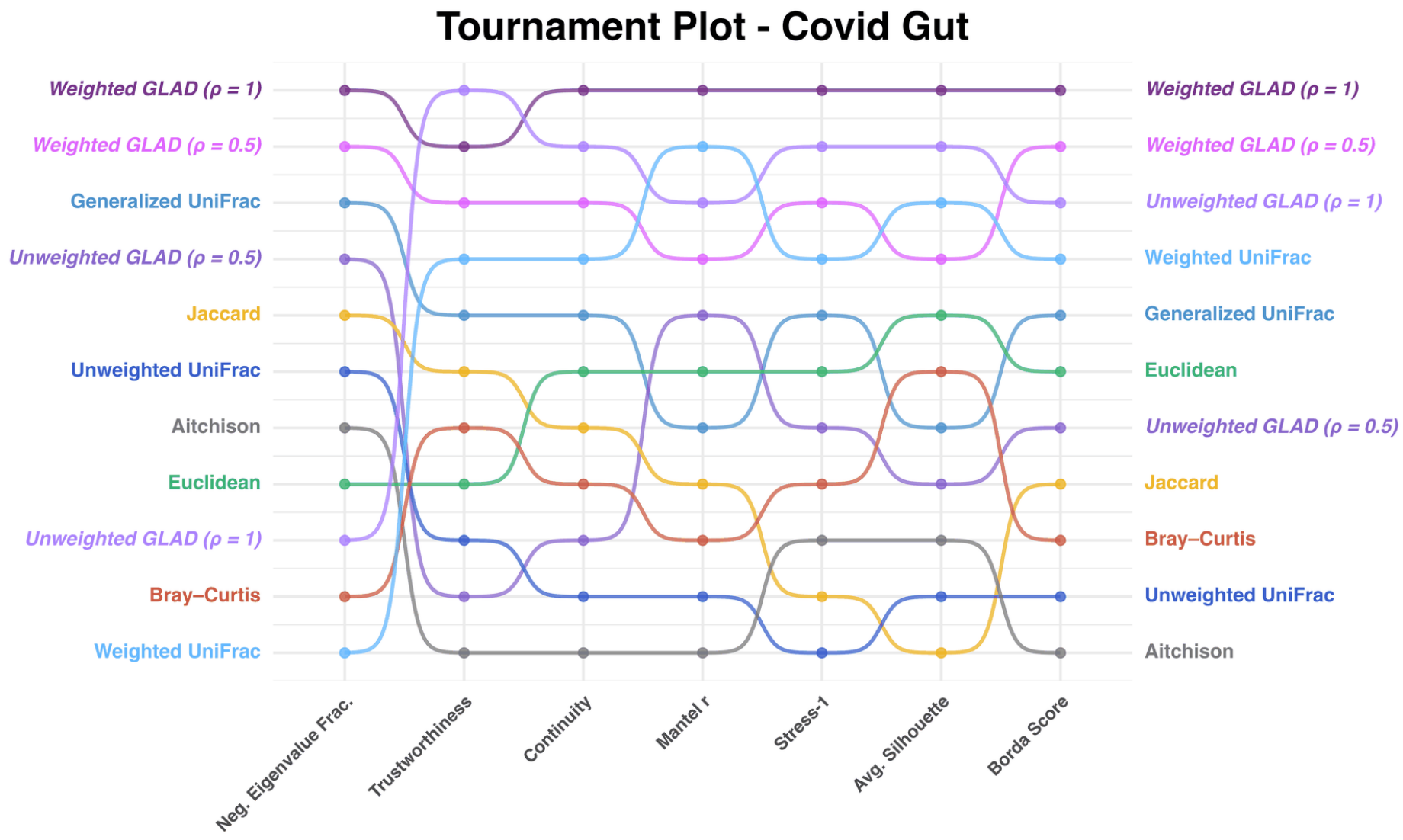

(a) COVID–Gut

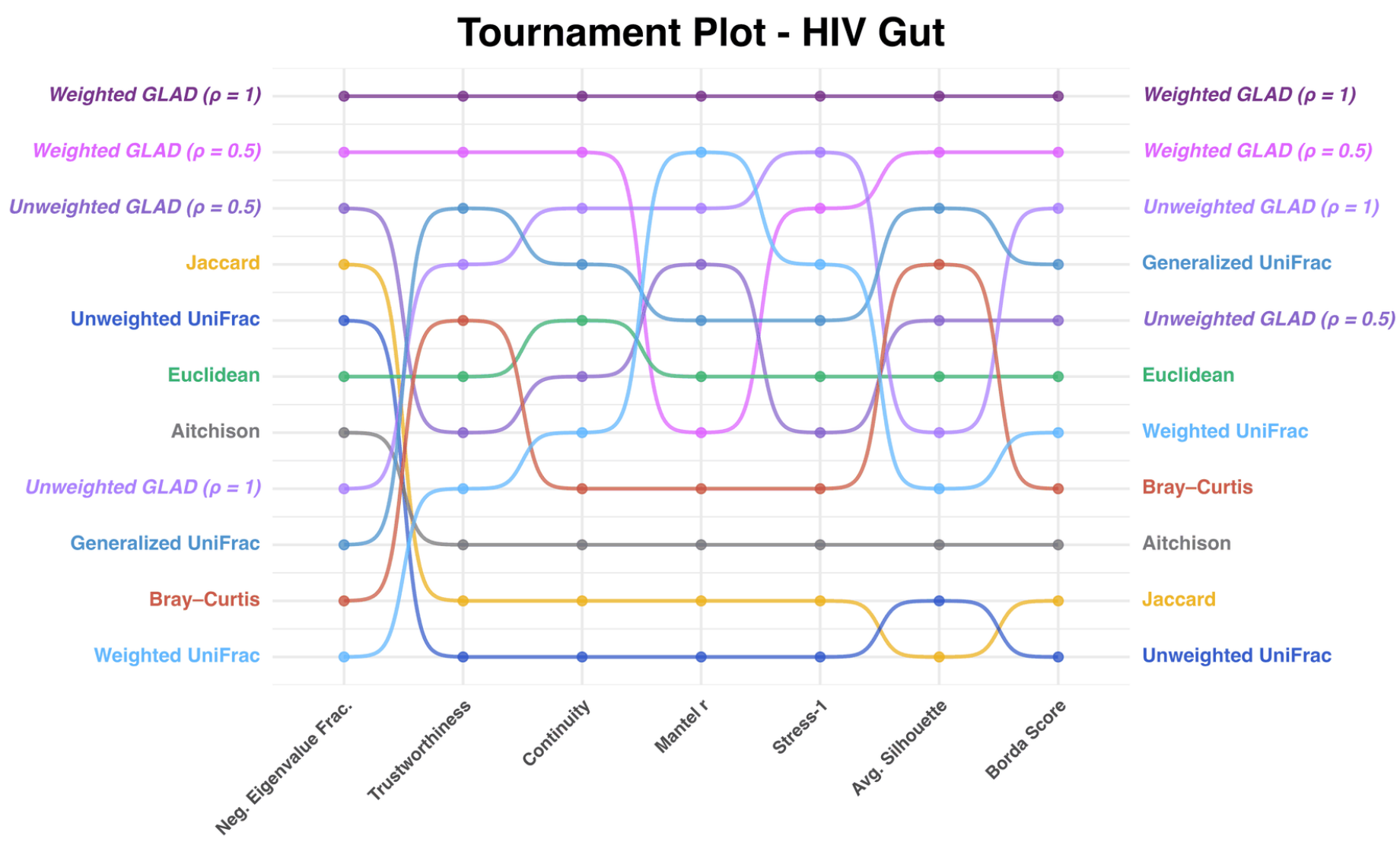

(b) HIV–Gut

Fig. S8: **Tournament-style rankings in the COVID–Gut and HIV–Gut datasets.** Trajectories rank eleven dissimilarities across the same six evaluation criteria and the aggregate Borda score shown in Fig. S7; rank 1 denotes the best result in each column. Purple and pink trajectories identify the four GLaD variants.

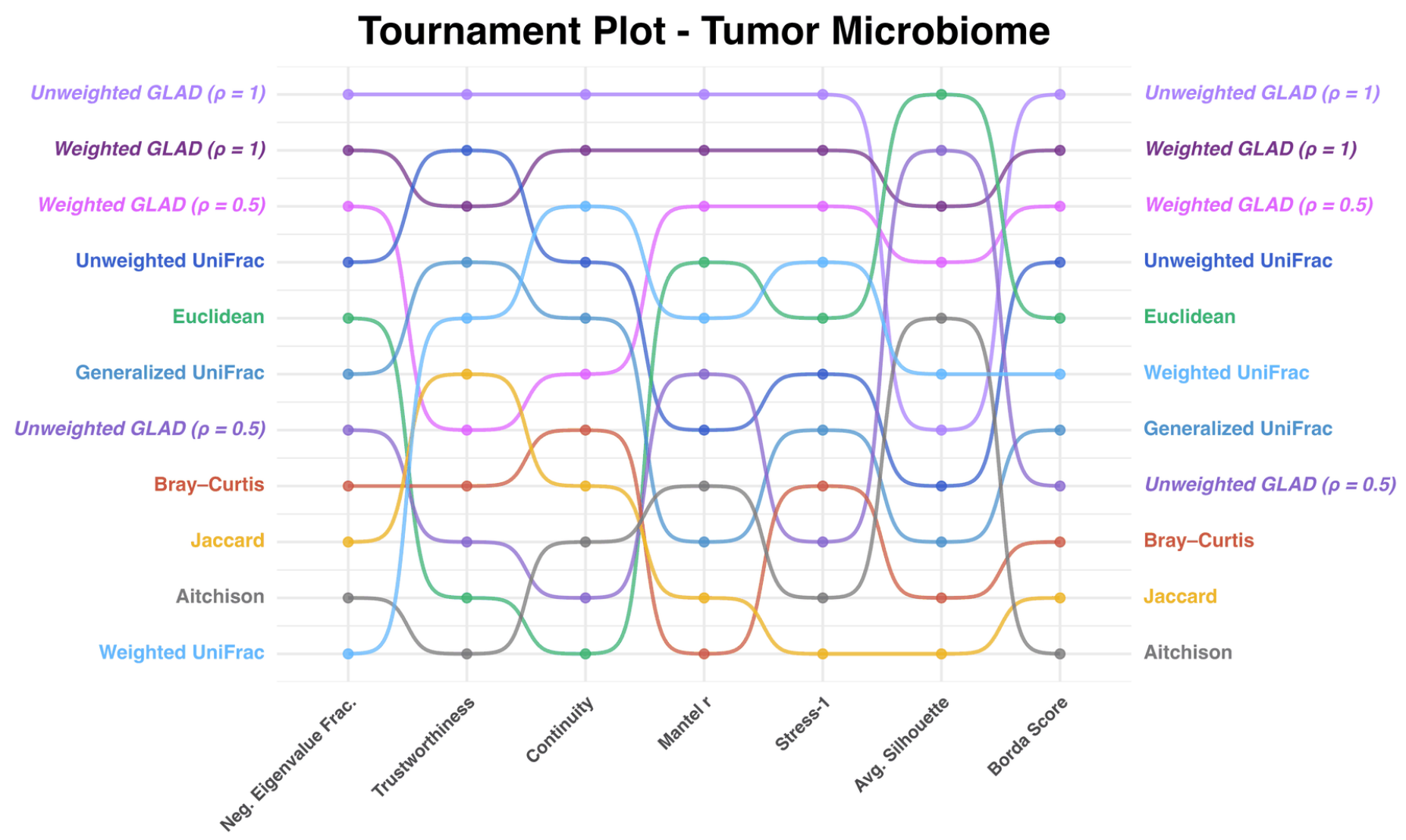

(a) Physeq–Tumor

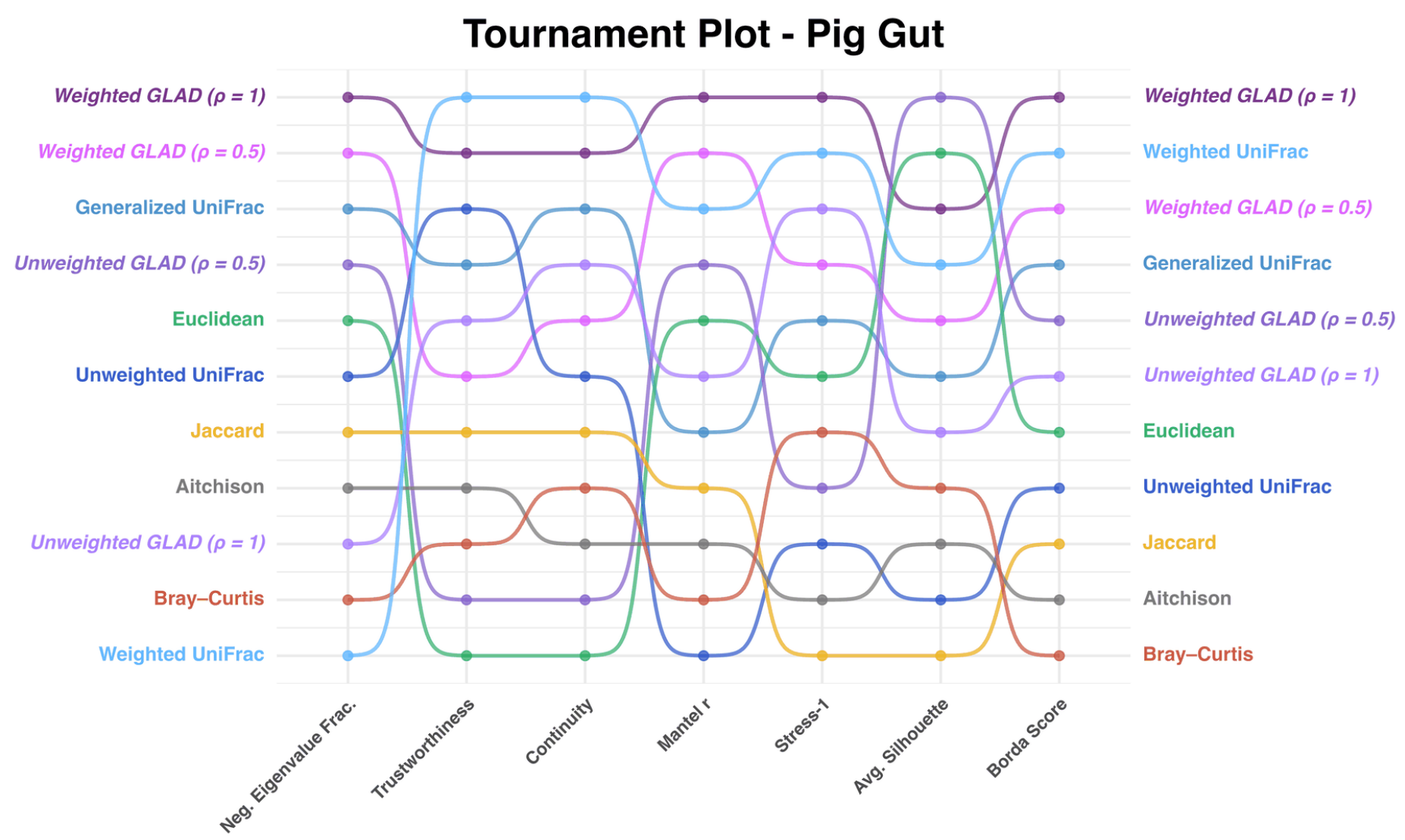

(b) Pig–Gut

Fig. S9: **Tournament-style rankings in the pancreatic tumor and pig gut datasets.** Trajectories rank eleven dissimilarities across the same six evaluation criteria and the aggregate Borda score shown in Fig. S7; rank 1 denotes the best result in each column. Purple and pink trajectories identify the four GLaD variants.

3.5 Additional Results of MiRKAT and PERMANOVA

Method	COVID-Gut Microbiome	HIV-Gut Microbiome	IBD Dataset (16S)	IBD Dataset (WGS)	ORIGINS Oral Microbiome (16S)	ORIGINS Oral Microbiome (WGS)	Pig Gut Microbiome
Unweighted GLaD (rho = 0.5)	0.0149878412073793	0.0153275649028121	0.0100373726433197	0.0113237443492472	0.00699304335447239	0.00574041749387385	0.0579711055880467
Weighted GLaD (rho = 0.5)	0.0133799365085271	0.0127070227665794	0.025747531545248	0.0360354267229295	0.0036472540721007	0.00695557881745284	0.139692985349216
Unweighted GLaD (rho = 1)	0.0097684339859425	0.0253318249855601	0.0123537989285816	0.04369381931631	0.00316583104924543	0.00613904085615933	0.115963853028217
Weighted GLaD (rho = 1)	0.0170891026480591	0.0122370716080884	0.037511130831282	0.0537616660822481	0.0021306857585626	0.00629094493000667	0.165476321214148
Unweighted UniFrac	0.0150704852400799	0.0140057531778553	0.00898916899594681	0.0113179576340612	0.00659147937513217	0.00637445998060269	0.0532625986991954
Weighted UniFrac	0.00881558500350201	0.00786297296103289	0.0103445230483557	0.00728493567361542	0.00282531310682368	0.00299883894712306	0.189482078593255
GUniFrac	0.0150474952020763	0.0135194952865358	0.0159747977852666	0.01842387311279	0.00524288189689665	0.00460396120401414	0.132296878491438
Aitchison	0.0134836176674398	0.0152451307500487	0.0090556645589528	0.0140083985538077	0.00660521329213851	0.00598022563337079	0.06719410076253
Bray-Curtis	0.0145163376426617	0.0146327988191488	0.0131367542471358	0.0129115467234165	0.00470476272305625	0.00521662515616079	0.09122671660429
Euclidean	0.0173954110556892	0.0133813815847787	0.022455198111469	0.0117672916527162	0.00276090360111132	0.00501907428543525	0.0530619843215109
Jaccard	0.0152932806938861	0.0139748097984163	0.0110845224409639	0.0106662551821342	0.00594530779998098	0.00634726752778705	0.0589204237737543

Table S10: MiRKAT R^2 values for each dissimilarity method (rows) and case-study dataset (columns). Values are taken directly from the case-study analysis output, with larger R^2 indicating stronger association in MiRKAT.

Method	COVID-Gut Microbiome	HIV-Gut Microbiome	IBD Dataset (16S)	IBD Dataset (WGS)	ORIGINS Oral Microbiome (16S)	ORIGINS Oral Microbiome (WGS)	Pig Gut Microbiome
Unweighted GLaD (rho = 0.5)	1.40768441731925	1.21959761449346	1.14173234393476	1.18426188719749	1.11262905101439	1.03280604262512	3.57663359481683
Weighted GLaD (rho = 0.5)	1.07848477649634	1.14349662149673	1.64484445268556	1.49605999861106	0.825223526393869	1.38806225099778	9.30840482318906
Unweighted GLaD (rho = 1)	1.99402087272534	1.5871060096317	1.52005376084204	1.71228922408969	1.33743398388988	1.27956952423746	6.9933325761544
Weighted GLaD (rho = 1)	0.935052637190575	1.09726422193855	1.66691854510688	1.72702693589859	0.797849265366946	1.34146332028008	11.5651310588567
Unweighted UniFrac	1.30057706684597	1.16121501190494	1.19700292224799	1.17581041136008	1.22786319216055	1.03748530959207	3.37326318214254
Weighted UniFrac	1.85404468559508	0.938813630908394	0.986425589504026	1.02117213142003	0.869753892330772	1.12127762922252	10.9242352892119
GUniFrac	1.40479442992655	1.20288244056672	1.4980548777112	1.38883768897162	1.02407519613891	1.30572206550081	7.81932207699324
Aitchison	1.57557758104972	1.13900082669769	1.21708962404184	1.30598214644855	1.0936097684846	1.18434231548726	4.15343880630297
Bray-Curtis	1.45360322026887	1.25060320754921	1.38867336885877	1.31193729858609	0.938483383241681	1.10408359633648	5.46593178996334
Euclidean	1.21405305379243	0.936583533277882	1.38656354887358	1.23797827414522	0.718003802323172	1.78078568965633	3.49775769600906
Jaccard	1.29266140669147	1.17050553592439	1.32151267182929	1.269410304135	1.0056075945645	1.1794071640278	3.54906259364164

Table S11: PERMANOVA pseudo-F statistics for each dissimilarity method (rows) and case-study dataset (columns). Values are taken directly from the case-study analysis output, with larger pseudo-F indicating stronger separation of groups under PERMANOVA.

References

[1] Gower JC, Legendre P. Metric and Euclidean properties of dissimilarity coefficients. Journal of classification. 1986;3(1):5–48.

[2] Menger K. Untersuchungen über allgemeine Metrik. Mathematische Annalen. 1928;100:75–163.

[3] Zhu Z, Zhang Y, Li W, Greenacre M, Satabdi S, Shi Y, et al. Mathematical Foundations of Beta Diversity: Why Common Metrics Fail in Microbiome Analysis. Journal of Statistical Theory and Applications. 2026;9. https://doi.org/10.1007/s44199-025-00154-7.

[4] Parks SL, Goldman N. Maximum likelihood inference of small trees in the presence of long branches. Systematic Biology. 2014;63(5):798–811.

[5] Lemoine F, Gascuel O. The Bayesian phylogenetic bootstrap and its application to short trees and branches. Molecular biology and evolution. 2024;41(11):msae238.

[6] Lozupone C, Knight R. UniFrac: a new phylogenetic method for comparing microbial communities. Applied and environmental microbiology. 2005;71(12):8228–8235.

[7] Lozupone CA, Hamady M, Kelley ST, Knight R. Quantitative and qualitative β diversity measures lead to different insights into factors that structure microbial communities. Applied and environmental microbiology. 2007;73(5):1576–1585.

[8] Pavoine S, Dufour AB, Chessel D. From dissimilarities among species to dissimilarities among communities: a double principal coordinate analysis. Journal of theoretical biology. 2004;228(4):523–537.

[9] Smola AJ, Kondor R. Kernels and regularization on graphs. In: Learning theory and kernel machines: 16th annual conference on learning theory and 7th kernel workshop, COLT/kernel 2003, Washington, DC, USA, august 24-27, 2003. Proceedings. Springer; 2003. p. 144–158.

[10] Venna J, Kaski S. Neighborhood preservation in nonlinear projection methods: An experimental study. In: International conference on artificial neural networks. Springer; 2001. p. 485–491.

[11] Venna J, Peltonen J, Nybo K, Aidos H, Kaski S. Information retrieval perspective to nonlinear dimensionality reduction for data visualization. Journal of Machine Learning Research. 2010;11(2).

[12] Mantel N. The detection of disease clustering and a generalized regression approach. Cancer research. 1967;27(2_Part_1):209–220.

[13] Kaufman L, Rousseeuw PJ. Finding groups in data: an introduction to cluster analysis. New York: John Wiley & Sons; 2009.

[14] Tibshirani R, Walther G, Hastie T. Estimating the number of clusters in a data set via the gap statistic. Journal of the royal statistical society: series b (statistical methodology). 2001;63(2):411–423.

[15] Rousseeuw PJ. Silhouettes: a graphical aid to the interpretation and validation of cluster analysis. Journal of computational and applied mathematics. 1987;20:53–65.

[16] Caliński T, Harabasz J. A dendrite method for cluster analysis. Communications in Statistics-theory and Methods. 1974;3(1):1–27.

[17] Dunn JC. Well-separated clusters and optimal fuzzy partitions. Journal of cybernetics. 1974;4(1):95–104.

[18] Borda Jd. M'emoire sur les' elections au scrutin. Histoire de l'Acad'emie Royale des Sciences. 1781;.

[19] Emerson P. The original Borda count and partial voting. Social Choice and Welfare. 2013;40(2):353–358.

[20] Ma S, Ren B, Mallick H, Moon YS, Schwager E, Maharjan S, et al. A statistical model for describing and simulating microbial community profiles. PLoS computational biology. 2021;17(9):e1008913.

[21] Demmer R, Jacobs Jr D, Singh R, Zuk A, Rosenbaum M, Papapanou P, et al. Periodontal bacteria and prediabetes prevalence in ORIGINS: the oral infections, glucose intolerance, and insulin resistance study. Journal of dental research. 2015;94(9_suppl):201S–211S.

[22] Demmer RT, Breskin A, Rosenbaum M, Zuk A, LeDuc C, Leibel R, et al. The subgingival microbiome, systemic inflammation and insulin resistance: the oral infections, glucose intolerance and insulin resistance study. Journal of clinical periodontology. 2017;44(3):255–265.

[23] Yin YS, Minacapelli CD, Parmar V, Catalano CC, Bhurwal A, Gupta K, et al. Alterations of the fecal microbiota in relation to acute COVID-19 infection and recovery. Molecular biomedicine. 2022;3(1):36.

[24] Armstrong AJ, Quinn K, Fouquier J, Li SX, Schneider JM, Nusbacher NM, et al. Systems analysis of gut microbiome influence on metabolic disease in HIV-positive and high-risk populations. MSystems. 2021;6(3):10–1128.

[25] Gonzalez CG, Mills RH, Zhu Q, Sauceda C, Knight R, Dulai PS, et al. Location-specific signatures of Crohn's disease at a multi-omics scale. Microbiome.

2022;10(1):133.

[26] Mills RH, Dulai PS, Vázquez-Baeza Y, Sauceda C, Daniel N, Gerner RR, et al. Multi-omics analyses of the ulcerative colitis gut microbiome link Bacteroides vulgatus proteases with disease severity. Nature microbiology. 2022;7(2):262–276.

[27] Curtasu MV, Tafintseva V, Bendiks ZA, Marco ML, Kohler A, Xu Y, et al. Obesity-related metabolome and gut microbiota profiles of juvenile göttingen minipigs—Long-term intake of fructose and resistant starch. Metabolites. 2020;10(11):456.

[28] Bolyen E, Rideout JR, Dillon MR, Bokulich NA, Abnet CC, Al-Ghalith GA, et al. Reproducible, interactive, scalable and extensible microbiome data science using QIIME 2. Nature biotechnology. 2019;37(8):852–857.

[29] Callahan BJ, McMurdie PJ, Rosen MJ, Han AW, Johnson AJA, Holmes SP. DADA2: High-resolution sample inference from Illumina amplicon data. Nature methods. 2016;13(7):581–583.

[30] Yilmaz P, Parfrey LW, Yarza P, Gerken J, Pruesse E, Quast C, et al. The SILVA and "all-species living tree project (LTP)" taxonomic frameworks. Nucleic acids research. 2014;42(D1):D643–D648.

[31] Price MN, Dehal PS, Arkin AP. FastTree 2–approximately maximum-likelihood trees for large alignments. PloS one. 2010;5(3):e9490.

[32] Zhu Q, Huang S, Gonzalez A, McGrath I, McDonald D, Haiminen N, et al. Phylogeny-aware analysis of metagenome community ecology based on matched reference genomes while bypassing taxonomy. Msystems. 2022;7(2):e00167–22.

[33] Zhu Q, Mai U, Pfeiffer W, Janssen S, Asnicar F, Sanders JG, et al. Phylogenomics of 10,575 genomes reveals evolutionary proximity between domains Bacteria and Archaea. Nature communications. 2019;10(1):5477.

[34] Paradis E, Schliep K. ape 5.0: an environment for modern phylogenetics and evolutionary analyses in R. Bioinformatics. 2019;35(3):526–528.
